# Separating individual and group-level cooperation in the Public Goods Game

**DOI:** 10.1093/pnasnexus/pgae200

**Published:** 2024-05-17

**Authors:** Yngwie Asbjørn Nielsen, Stefan Pfattheicher

**Affiliations:** Department of Psychology and Behavioural Sciences, Aarhus University, 8000 Aarhus, Denmark; Department of Linguistics, Cognitive Science and Semiotics, Aarhus University, 8000 Aarhus, Denmark; Department of Psychology and Behavioural Sciences, Aarhus University, 8000 Aarhus, Denmark

**Keywords:** cooperation, intraclass correlation (ICC), Public Goods Game (PGG), variance partitioning

## Abstract

Cooperation in the Public Goods Game (PGG) is determined by a mixture of individual differences (e.g. personality, social preferences) and group dynamics (e.g. reciprocation, social norms). However, to our knowledge, no thorough attempt has been made to separate individual and group levels of cooperation and to quantify the variance in cooperation that can be attributed to the group level. In an analysis of 10 open datasets (total *N* = 4,556, 1,003 groups, 7–50 rounds), we chart the trajectory of individual and group-level variance across rounds of repeated PGGs. We find that the portion of group-level variance increases initially and plateaus around the fifth round, typically at a level between 20 and 50%. In addition, we identify four factors that increase the portion of group-level variance: (i) punishment opportunities; (ii) detailed feedback including all group members' decisions; (iii) small groups (≤4 players); and (iv) groups with homogenous social preferences.

**Significance Statement**

What makes people cooperate? Scholars believe the answer to lie at the intersection of individual tendencies and group dynamics. Yet, no study to date has thoroughly quantified the relative importance of individual and group-level cooperation. Using a classic paradigm of cooperation—the Public Goods Game—we find that people converge on their group members' levels of cooperation within five rounds of interaction. This convergence accounts for at least 20 to 50% of the total cooperation and up to 70% if players are given tools to enforce group norms (e.g. feedback on each other's decisions, the option to punish each other). Cooperation thus hinges on both individual and group processes, and neglecting either results in a substantial loss of insight.

## Introduction

The Public Goods Game (PGG) (1) is a gold-standard paradigm in the study of cooperation (2). In it, each player in a group is endowed with a sum of money and decides to contribute any amount (or nothing) to a shared pool. The contributions in the shared pool are then multiplied by some factor above one, yet smaller than the group size, and shared equally. The players thus face a dilemma between contributing to benefit the collective and keeping their money to maximize their own payoff (i.e. free-riding). Despite its apparent simplicity, the PGG and its variants have been used to model complex real-life dilemmas such as migration (3), vaccination (4), and mitigating climate change (5).

Multiple theories have spawned to explain behavior in the PGG (2). Some theories stress the importance of individual differences, highlighting how personality traits (6), or social preferences (7, 8) shape the decision to cooperate. Other theories have focused on the group, highlighting how individuals with different preferences interact to create group-level patterns of cooperation. For instance, groups of strongly inequity-averse individuals may reach certain “fair” equilibria [e.g. everyone contributing the same amount (9, 10)]. Cooperation may break down, however; if a group is composed of conditional cooperators (who reciprocate both cooperation and free-riding) and one or more selfish individuals (7, 8). In summary, cooperation in the PGG depends on both individual differences and group dynamics.

Despite the interest in understanding cooperation in the PGG at both an individual and group level, there is to our knowledge no thorough attempt at separating the two levels statistically [but see Ref. (11)]. We address this gap by analyzing 10 datasets of repeated PGGs (see Table 1). Our analysis makes use of multilevel modeling—a technique commonly used to separate individual and group-level variance (e.g. in the school context, to partition the variance in academic performance attributable to the student vs. the class)—to estimate the intraclass correlation (ICC) of cooperation. The ICC, roughly speaking, is the portion of the total variance which can be explained by knowing the average decision of each group (22). It ranges from zero to one, with zero indicating no within-group dependence, and one indicating that group members always contribute an identical amount. It is, however, important to note that the ICC cannot perfectly distinguish individual and group processes, and there are group processes that lead to divergent decisions among group members. For instance, so-called hump-shaped or triangle cooperators tend to lower their contributions when the average group contribution surpasses a certain threshold (7, 8). Such diverging group processes are not captured by the ICC and may in fact reduce it. Instead, the ICC may be interpreted as a measure of *group convergence*, that is, the extent to which players tend to contribute similarly to other members of their group.

**Table 1.** Overview of datasets.

		Sample		Game parameters							
Dataset	Portion used	Participants (groups)	Setting	Group size	Endowment	Exchange rate	MPCR	Rounds	Norm enforcement	Feedback	Endgame knowledge
Burton-Chellew and Guérin (12)	All	616 (154)	Laboratory	4	20	0.025 CHF	0.4	9	None	None/detailed/ aggregate	Yes
Diederich et al. (13)	Main (*L*) treatments	860 (26)	Online	10/40/100	40	0.01 EUR	0.3	7	None	Aggregate	Yes
Grandjean et al. (14)	All	192 (64)	Laboratory	3	20	0.025 EUR	0.6	15	None	Aggregate	Yes
Gächter et al. (15)	Provision PGG	296 (74)	Laboratory	4	20	0.2 GBP	0.4	27	None/ punishment	Aggregate/ detailed	No
Nosenzo et al. (16)	All	364 (85)	Laboratory	2/3/4/8	20	0.003 GBP/ 0.0075 GBP	0.3/0.75	10	None	Detailed	Yes
Stagnaro et al. (17)	All	516 (172)	Online	3	140	0.001 USD	0.4/0.5	10	None/ Institutional punishment	Detailed	Yes
Rand et al. (18)	All	192 (48)	Laboratory	4	20	0.008 USD	0.4	50	None/ punishment/ reward/both	Detailed	No
Gross et al. (19)	Control condition	80 (20)	Online	4	20	0.001 USD	0.4	30	Yes	Detailed	Yes (but not restart)
Herrmann et al. (20)	All	1,120 (280)	Laboratory	4	20	0.03 USD	0.4	20	None/ punishment	Detailed	Yes (but not restart)
Arechar et al. (21)	All	320 (80)	Online/ laboratory	40	20	0.01 USD/ 0.02 USD	0.4	20	None/ punishment	Detailed	Yes (but not restart)

Slash marks indicate that a parameter was manipulated across experimental conditions.

MPCR, marginal per capita return.

We assumed that the individual level would dominate in the early rounds of the PGGs, as players so far had little opportunity to learn from and adapt to their group members. Then, through mutual social influence, some groups would begin to converge in their contributions. Thus, we hypothesized (cf. the pre-registration) that the ICC would start close to zero, rise over the first couple rounds, and then plateau and remain stable at a medium level (i.e. 0.20 < ICC < 0.50) as some groups eventually reached convergent equilibria. Due to the existence of strong social preferences [e.g. unconditional cooperators that contribute despite free-riding group members (7)], we expected the ICC would never reach one.

## Results

### Groups converge in the initial rounds

**Fig. 1.**
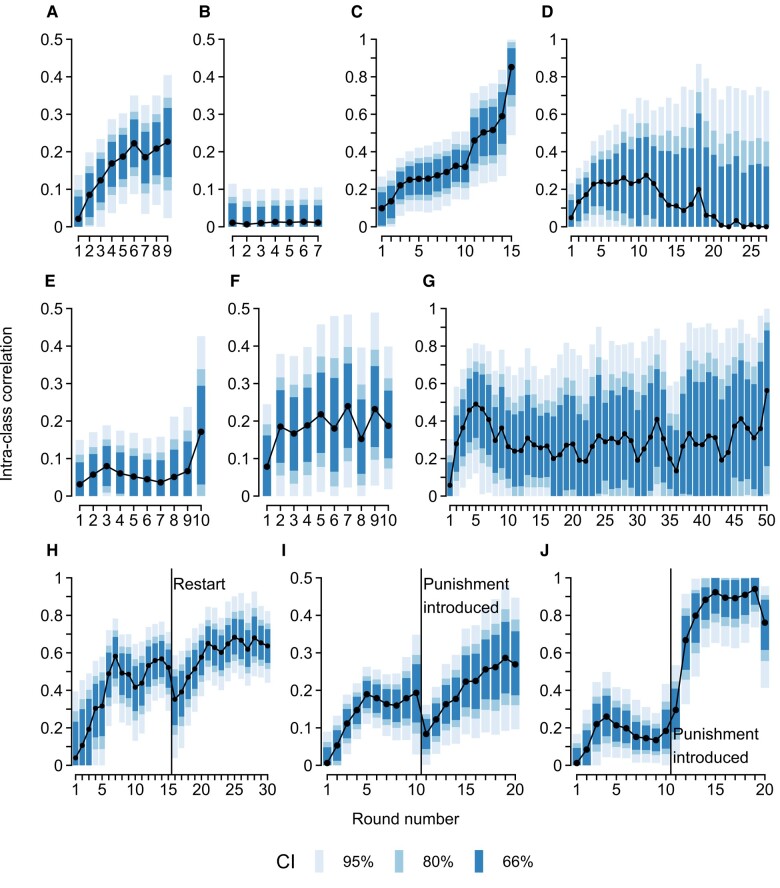
ICCs across rounds of repeated PGGs. A) Burton-Chellew and Guérin (12), excluding the condition without feedback. B) Diederich et al. (13). C) Grandjean et al. (14). D) Gächter et al. (15) excluding the condition with stranger matching. E) Nosenzo et al. (16). F) Stagnaro et al. (17). G) Rand et al. (18). H) Gross et al. (19). I) Herrmann et al. (20) excluding the condition with punishment first. J) Arechar et al. (21). CIs extending below zero are not shown. CI, credible interval.

Using a Bayesian multilevel model, we charted the ICC across the rounds of repeated PGG (see Fig. 1; for additional information and illustrations, see Supplementary Materials). The expected pattern emerged in eight of the 10 datasets, that is, the ICC started near zero and increased in the following rounds (probabilities of direction [*pd*s] ranging from 0.826 to 1.000, evidence ratios [ERs] ranging from 4.7 to 5999.0). Two exceptions were Nosenzo et al. (16), where the ICC never broke above 0.10 (*pd* = 0.708, ER = 2.4; Fig. 1E), and Diederich et al. (13), where the ICC was consistently around zero (*pd* = 0.492, ER = 1.0; Fig. 1B). Notably, these datasets were the only to consider larger group sizes (>4 players), which may explain the divergent results (see below). Overall, although unsurprising, these results serve as a sanity check that group members converge as they interact.^1^

### Group convergence plateaus after five rounds

After the initial increase, the ICC tended to reach a plateau. Generally speaking, the ICC required five rounds to stabilize and—in studies employing standard game parameters—it typically did so within the range of 0.20 to 0.50. Curiously, even after 30 rounds (Fig. 1H) or even 50 rounds of interaction (Fig. 1G), the ICC was still far from one, suggesting that some groups never fully converge [e.g. due to major differences in social preferences (7–9)]. Overall, our findings align with Gächter et al. (11), who estimated the ICC to be 0.293. Yet, we also found substantial heterogeneity in both the timing and level of the plateau: In some studies, the ICC continued changing, sometimes reaching multiple plateaus (see Fig. 1), and the level of these plateaus ranged from below 0.10 (Fig. 1E) to above 0.70 (Fig. 1H and J). Importantly, there were systematic differences in the game parameters applied in each study (see Table 1) which may underlie the observed heterogeneity. We turn to this matter next.

### Punishment and feedback strengthen group convergence

**Fig. 2.**
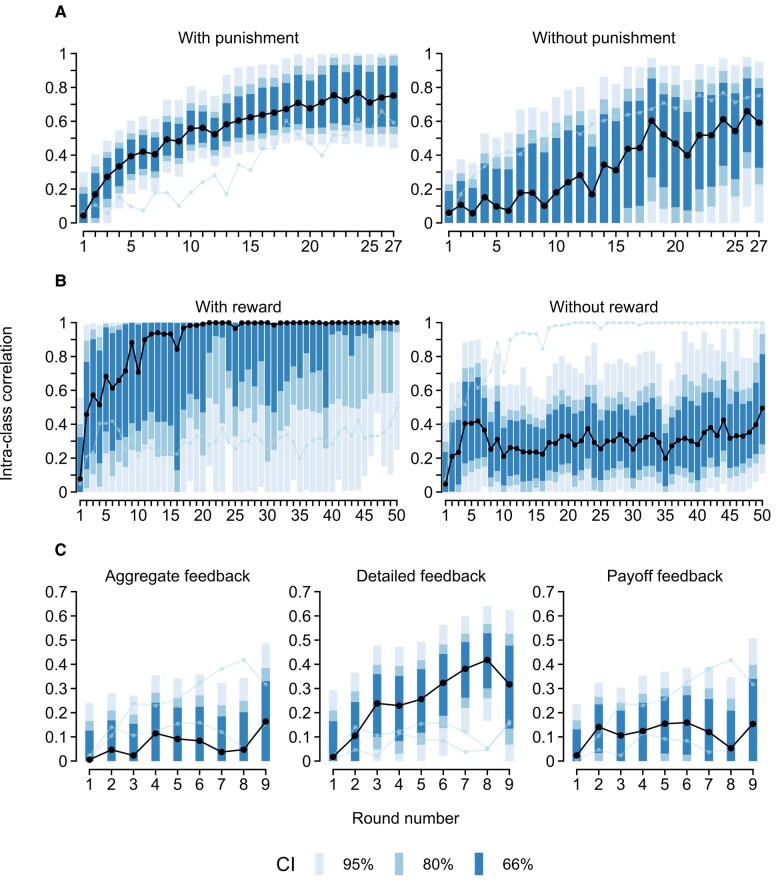
ICCs across rounds of repeated PGGs. A) Comparison across games with and without punishment in Gächter et al. (15). B) Comparison across games with and without reward in Rand et al. (18). C) Comparison across types of feedback in Burton-Chellew and Guérin (12). CIs extending below zero or above one are not shown. CI, credible interval.

What conditions facilitate convergence within groups? The analysis of Gächter et al. (15) suggests that the option to punish group members can serve to increase convergence (Fig. 2A). On average, the ICC was higher in games with punishment (0.580 [0.527; 0.631]) than in games without (0.294 [0.153; 0.410], *pd* = 1.000, ER = ∞). The same pattern is visible *within* groups in the data from Herrmann et al. (20) and Arechar et al. (21): After punishment is introduced in round 11, the ICC trajectory seemed to “restart” before settling at a higher plateau (*pd*s > 0.971, ERs > 33.9; Fig. 1I and J).^2^ The positive effect of punishment explains why the ICC was generally higher in the study by Gross et al. (19) who also employed punishment. In addition, these results are consistent with research suggesting that punishment (and other “strong situations”) can restrict individual-level variance in cooperation (11, 24) and enforce group norms (25). Curiously, the analysis of Rand et al. (18) shows that high group convergence can be enforced equally effectively via punishment (0.803 [0.701; 0.871]) or via reward (0.793 [0.630; 0.878], *pd* = 0.561, ER = 1.3; Fig. 2B).

In addition to punishment, the analysis of Burton-Chellew and Guérin (12) suggests that the type of feedback players receive in-between rounds makes a difference (Fig. 2C). The average ICC in games where players are shown the decisions of each group member (0.255 [0.173; 0.327]) was larger than in games where the group average is shown (0.106 [0.014; 0.194], *pd* = 0.992, ER = 124.0), only the player's payoff is shown (0.118 [0.003; 0.214], *pd* = 0.984, ER = 61.5), or no feedback is given (0.119 [−0.016; 0.239], *pd* = 0.971, ER = 32.9; not shown in Fig. 2C). That is, when players know the individual decisions of other group members, they more readily converge on an equilibrium in the group.^3^

### Small and homogenous groups converge more readily

**Fig. 3.**
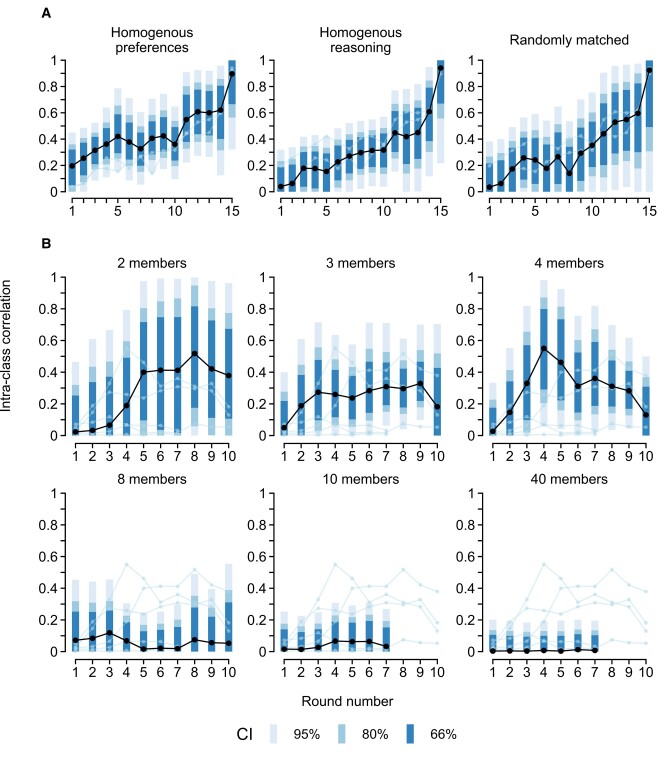
ICCs across rounds of repeated PGGs. A) Comparison across matching algorithms in Grandjean et al. (14). B) Comparison across group sizes. Panels with 2–8 members are from the high-MPCR condition (MPCR = 0.75) in Nosenzo et al. (16). Panels with 10–40 members are from Diederich et al. (13). CIs extending below zero or above one are not shown. CI, credible interval.

Which groups are most inclined to converge? The analysis of Grandjean et al. (14) suggests that group homogeneity matters (see Fig. 3A). Groups with homogenous social preferences obtained a higher average ICC (0.462 [0.376; 0.547]) than groups with homogenous reasoning ability (0.333 [0.239; 0.418], *pd* = 0.987, ER = 75.0) and randomly matched groups (0.333 [0.197; 0.449], *pd* = 0.968, ER = 30.3). This indicates that groups are better at reaching equilibria when the individual social preferences are in alignment (e.g. groups of fair-minded individuals converge on everyone contributing the same). Such an interpretation aligns with Gächter and Thöni (26), who conclude that cooperation norms are easier to uphold in groups of like-minded people. However, there is a risk that this result is an artifact of the group-matching. For instance, a group of selfish players independently choosing to free-ride would nonetheless boost the ICC. This confound is supported by weak evidence that groups with homogenous preferences already exhibited a slightly higher ICC in the first round of play (0.196 [−0.139; 0.450], *pd*s > 0.746, ERs > 2.9).

In addition to homogeneity, the size of the group appears to be a crucial determinant of convergence (see Fig. 3B). The analyses of Nosenzo et al. (16) and Diederich et al. (13) revealed that smaller groups consisting of up to four players tended to converge more than larger groups (four members: 0.326 [0.184; 0.464]; eight members: 0.047 [−0.090; 0.167], *pd* = 0.999, ER = 749.0). This result also explains the generally low ICC found in these studies and particularly in Diederich et al. (13), who only considered groups of ten or more players. Note, however, that the comparison across the studies (and hence across groups of eight or fewer players vs. groups of 10 or more players) is confounded and could stem from differences in marginal per capita return (MPCR) or the feedback players receive between rounds.

### Data synthesis

**Fig. 4.**
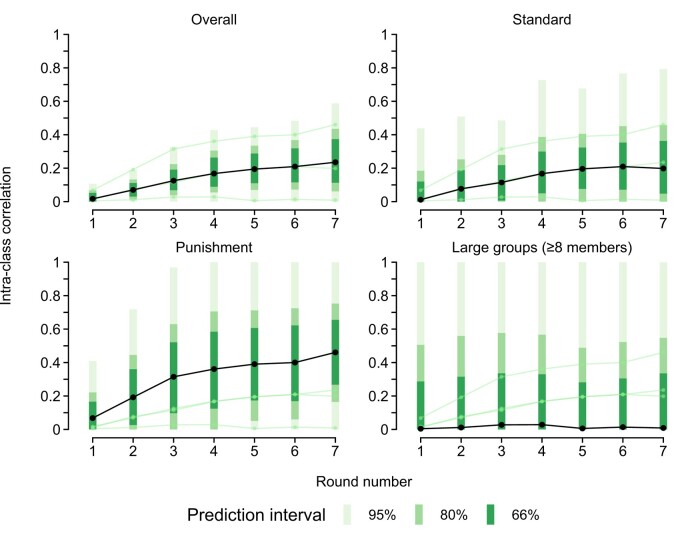
Meta-analytic estimates and prediction intervals for ICCs across rounds of repeated PGGs. Prediction intervals extending below zero or above one are not shown.

What ICCs can we expect from future research? We conducted a three-level random effects meta-analysis on the estimates from rounds one through seven (i.e. the largest round available in all datasets). Figure 4 displays prediction intervals derived from the model. Prediction intervals are estimates of the expected true value in a future (similar) study, accounting for between-study heterogeneity (27). In addition to an overall estimate, we provide results of three subgroup analyses, namely for “standard” games (i.e. four group members, an endowment of 20, and an MPCR of 0.40; *k* = 4, *N* = 1,596, 399 groups), standard games with punishment (*k* = 5, *N* = 1,292, 323 groups), and games with large group sizes (*k* = 2, *N* = 1,020, 46 groups). In the seventh round of a standard game, a future researcher can expect to see an ICC of 0.199 [−0.099; 0.487]. With punishment, the estimate increases to 0.461 [0.124; 0.789], and with large groups it drops to 0.010 [−0.677; 0.719].

## Discussion

After five rounds of repeated interaction, the portion of group-level variance in cooperation is substantial and nonignorable, lying between 20 and 50% in PGGs with typical game parameters. Under the right conditions (i.e. punishment, detailed feedback, small homogenous groups), the portion can be even higher, sometimes reaching 70%. At face value, this result affirms that cooperation in the (repeated) PGG is strongly influenced by group dynamics. Or, put differently, individuals may be nudged away from their selfish or unselfish ways when put in the right group. This result, however, does not undermine the importance of individual differences. On the contrary, individual differences (e.g. fairness concerns, conditional cooperation, selfishness) may actually be driving the convergence within groups. Indeed, group convergence is most likely the result of an interplay between individual differences and group processes (cf. conditional cooperators that attempt to match each other's contributions).

Likewise, it is important to keep in mind that a high ICC does not capture the influence of group processes per se, but rather the tendency for group members to *converge* in their decisions. Some players may actively decide to diverge from their group members [e.g. hump-shaped or triangle cooperators (7, 8)], which would reduce the ICC despite being dependent on a group-level process. Accordingly, one should be wary of “flipping the estimate” and claiming that 50 to 80% of variance is accounted for by individual differences. The residual variance in the model may additionally stem from divergent group processes and measurement error and is thus not a direct reflection of individual differences.

Finally, instances of extremely high ICCs (i.e. estimates close to one) should be interpreted with caution. Consider the final round of Grandjean et al. (14), where the ICC surpasses 0.80 (see Fig. 1C). Here, the increase in ICC coincides with a massive upturn in the proportion of groups in the free-riding equilibrium. This turns the distribution of contributions strongly multimodal (with a large peak at zero as well as peaks at various nonzero values), making it increasingly difficult to account for the data without group-level intercepts, in turn, inflating the ICC. Even so, in the games with sustained cooperation [e.g. Gross et al. (19), shown in Fig. 1H], the ICC remains high, indicating that the ICC is not driven purely by the proportion of defecting groups.

These limitations notwithstanding, our results indicate that a large portion of variance in cooperation resides at the group level. A researcher can expect group convergence to account for at least 20% of the variance in cooperation if they employ standard PGG parameters and at least 40% if they employ punishment and detailed feedback (see Fig. 4). These estimates are non-negligible in both directions; neither the individual nor the group level can be ignored without a considerable loss of insight into cooperative behavior. We thus urge researchers to adopt a balanced perspective at group cooperation, considering the role of individual differences and group dynamics in tandem.

## Materials and methods

### Data

We use 10 publicly available datasets (see Table 1). Each analyzed dataset was shared publicly by the respective authors (see Data Availability section).

### Analysis

We fitted a Bayesian ordinal beta regression (28) with random intercepts for each group and separate estimates for each round. To calculate the ICC, we followed an approach similar to the Bayesian *R*^2^ (29). First, we extracted the posterior predictions from the model, once conditioning on groups (yielding ${\hat{y}}_{1}$), once without conditioning on groups (yielding ${\hat{y}}_{0}$). The ICC was then calculated as $1-\mathrm{Var}({\hat{y}}_{1})/\mathrm{Var}({\hat{y}}_{0})$.^4^ Because the ICC is expected to be zero at game start, we discounted the first round of play in all comparisons between experimental conditions. We report posterior medians and 95% credible intervals (CIs) based on quantiles. For the meta-analysis, we aggregated the posterior ICC values into means and standard deviations and fitted a Bayesian Gaussian metaregression with random intercepts within and between studies. We included the first seven rounds of each dataset in the meta-analysis. In the subgroup analysis of punishment, the first seven rounds after the introduction of punishment were used instead. We report prediction intervals based on quantiles. The analysis script and fitted models are available on the Open Science Framework (OSF) (https://osf.io/js9eq/).

### Pre-registration

The analyses were preregistered in three waves on the OSF (https://osf.io/js9eq/registrations). We made one deviation from the first pre-registration [pertaining to Burton-Chellew and Guérin (12), Grandjean et al. (14) and Gross et al. (19)]: Instead of fitting a zero-one-inflated beta regression, we used an ordinal beta regression (28). This allowed us to fit an identically shaped distribution to the data but with a simpler parametrization.

## Supplementary Material

pgae200_Supplementary_Data

## Data Availability

The analysis script and fitted models are available on the OSF (https://osf.io/js9eq/). Each analyzed dataset was made publicly available by the respective authors. Burton-Chellew and Guérin (12): https://osf.io/t4smj/. Diederich et al. (13): https://data.mendeley.com/datasets/8s3nys36rj/1. Grandjean et al. (14): https://osf.io/dqye4/. Gächter et al. (15): https://datadryad.org/stash/dataset/doi:10.5061/dryad.8d9t2. Nosenzo et al. (16): https://reshare.ukdataservice.ac.uk/853008/. Stagnaro et al. (17): https://davidrand-cooperation.com/s/Data-and-Code-From-Good-Institutions-to-Generous-Citizens.zip. Rand et al. (18): https://davidrand-cooperation.com/s/positive-interactions-promote-public-cooperation-data.txt. Gross et al. (19): https://osf.io/em653/. Herrmann et al. (20): https://datadryad.org/stash/dataset/doi:10.5061/dryad.87301. Arechar et al. (21): https://static-content.springer.com/esm/art%3A10.1007%2Fs10683-017-9527-2/MediaObjects/10683_2017_9527_MOESM2_ESM.zip.

## Notes

1 The players in Gross et al. (19) also exhibited a *restart effect*, that is, the tendency to revert to initial (often higher) levels of cooperation after an unexpected “restart” of the game (23). In the first round after restart (i.e. round 16), the ICC dropped slightly (from 0.522 [0.291; 0.712] to 0.352 [0.038; 0.587]) with moderate evidence (*pd* = 0.842, ER = 5.3; Fig. 1H). A similar phenomenon happens in Herrmann et al. (20), where the ICC drops as soon as punishment is introduced (*pd* = 0.877, ER = 7.1; Fig. 1I).

2 Stagnaro et al. (17) simulate a norm-enforcing institution by randomly inspecting decisions and punishing deviations from full cooperation. Such a punishment scheme may also be effective at fostering group convergence. However, in the data, overall cooperation reached near-ceiling levels, yielding highly imprecise estimates of the ICC due to the low overall variance in contributions.

3 We additionally tried to compare the effect of MPCR across conditions in Nosenzo et al. (16). However, in the conditions with low MPCR (MPCR = 0.30), free-riding became rampant in the later rounds. In turn, the total variance in cooperation approached zero, making the ICC estimates highly uncertain. We thus refrain from making any conclusions on the effect of MPCR.

4 This variance ratio is technically not an ICC but can be interpreted as such and—unlike the ICC—it generalizes to non-Gaussian regression models (22).
