# Supplementary material for: Separating individual and group-level cooperation in the Public Goods Game
Source: PNAS Nexus. 2024 May 17;3(5):pgae200. doi: 10.1093/pnasnexus/pgae200 (PMC11138113; doi:10.1093/pnasnexus/pgae200)
Supplement: pgae200_Supplementary_Data [file pgae200_supplementary_data.pdf]

Supplementary Information for
Separating individual and group-level cooperation in the Public Goods Game

Yngwie Asbjørn Nielsen & Stefan Pfattheicher

Correspondence concerning this article should be addressed to Yngwie Asbjørn Nielsen, Aarhus University, Jens Chr. Skous Vej 2, 8000 Aarhus C, Denmark. E-mail: yan@cc.au.dk

This PDF file includes:

Figures S1 to S8: Supplementary Figures for Burton-Chellew and Guérin (2022)
Figures S9 to S16: Supplementary Figures for Diederich et al. (2016)
Figures S17 to S24: Supplementary Figures for Grandjean et al. (2022)
Figures S25 to S32: Supplementary Figures for Gächter et al. (2017)
Figures S33 to S40: Supplementary Figures for Nosenzo et al. (2015)
Figures S41 to S48: Supplementary Figures for Stagnaro et al. (2017)
Figures S49 to S56: Supplementary Figures for Rand et al. (2009)
Figures S57 to S60: Supplementary Figures for Gross et al. (2022)
Figures S61 to S64: Supplementary Figures for Herrmann et al. (2008)
Figures S65 to S70: Supplementary Figures for Arechar et al. (2018)

Other supplementary materials for this manuscript include the following:

Analysis scripts and additional figures and tables (available on the Open Science Framework: https://osf.io/js9eq/)

Supplementary Figures for Burton-Chellew and Guérin (2022)

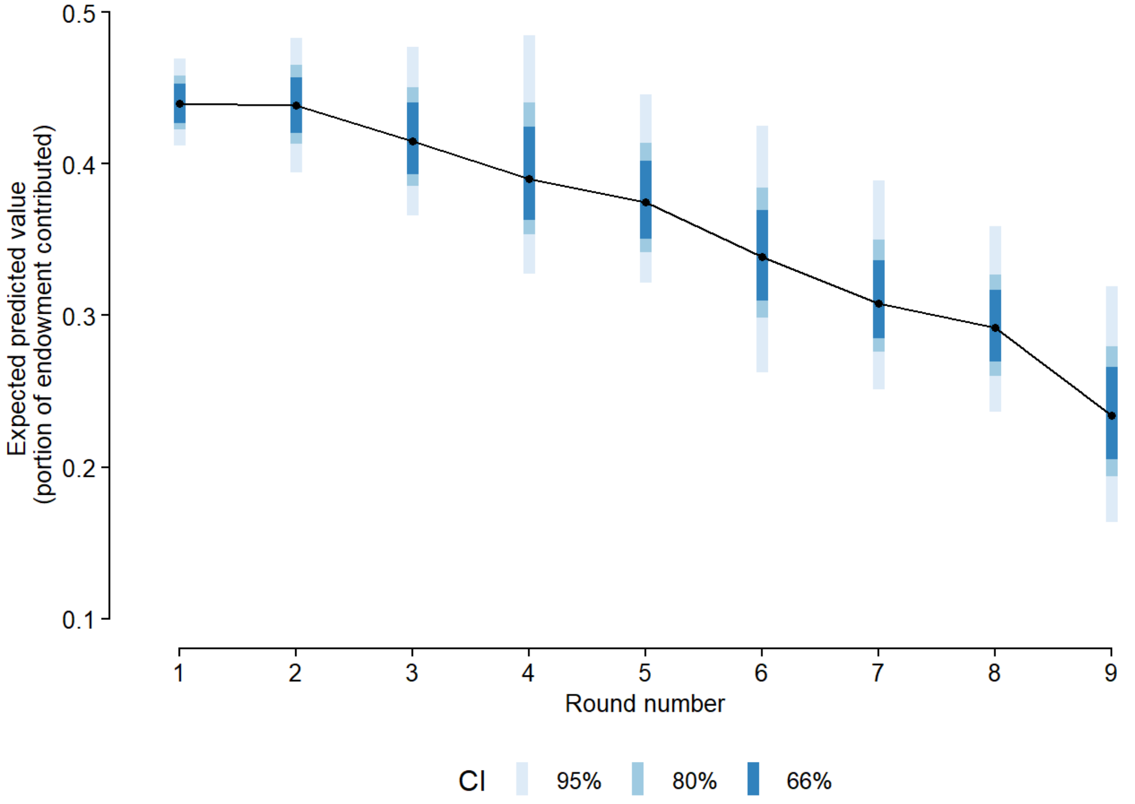

Fig. S1. Predicted level of cooperation across rounds of play.

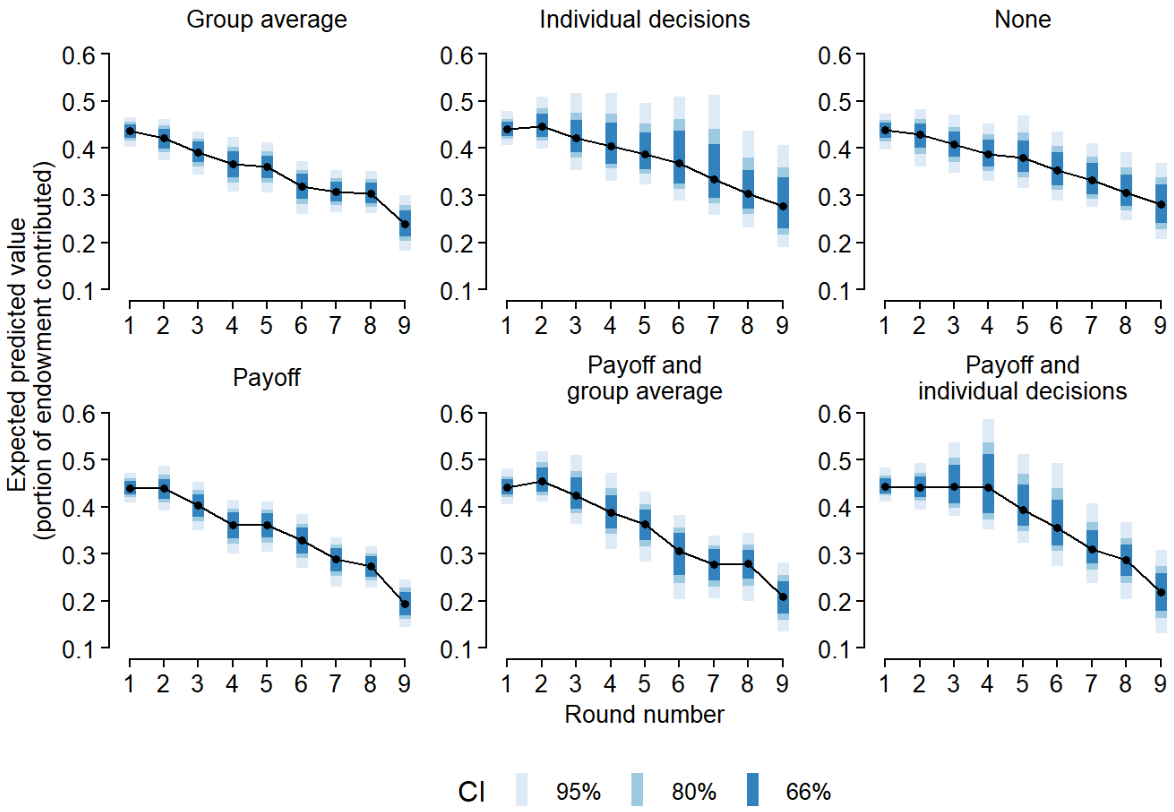

Fig. S2. Predicted level of cooperation across rounds of play in each experimental condition.

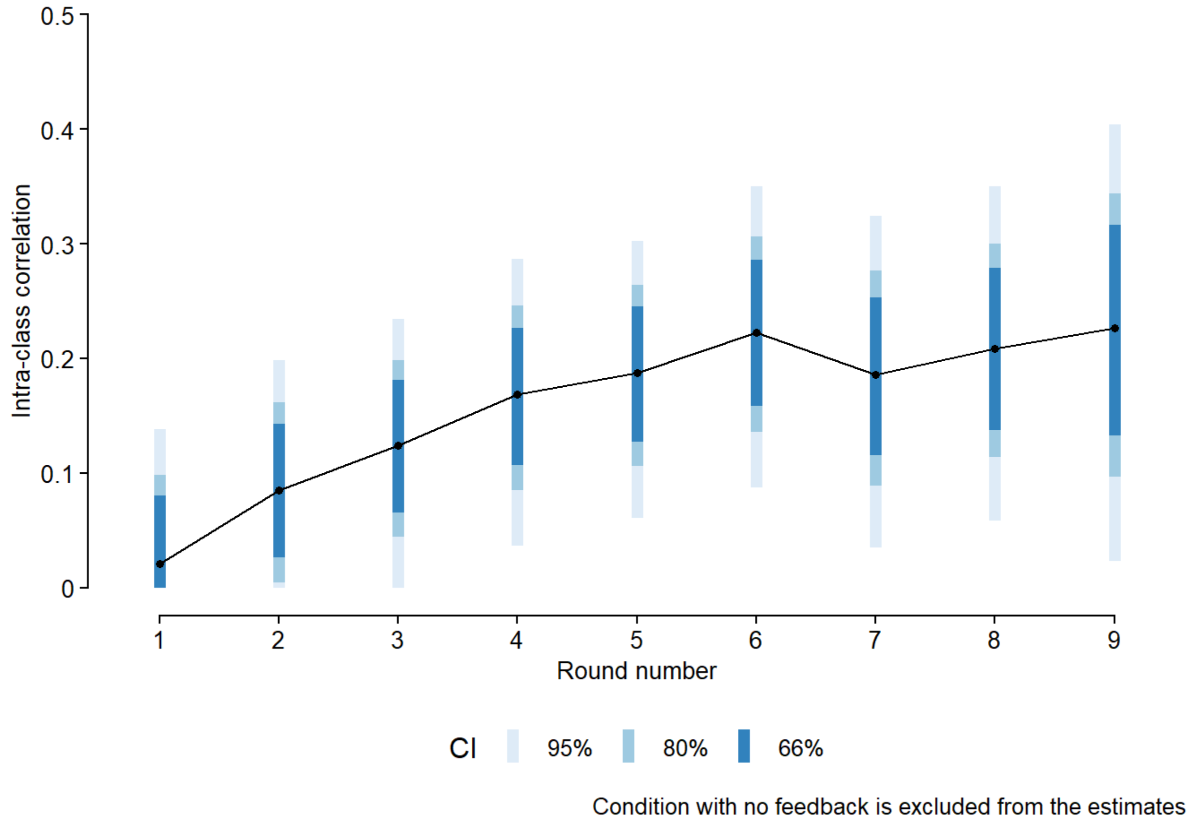

Fig. S3. Intra-class correlation across rounds of play.

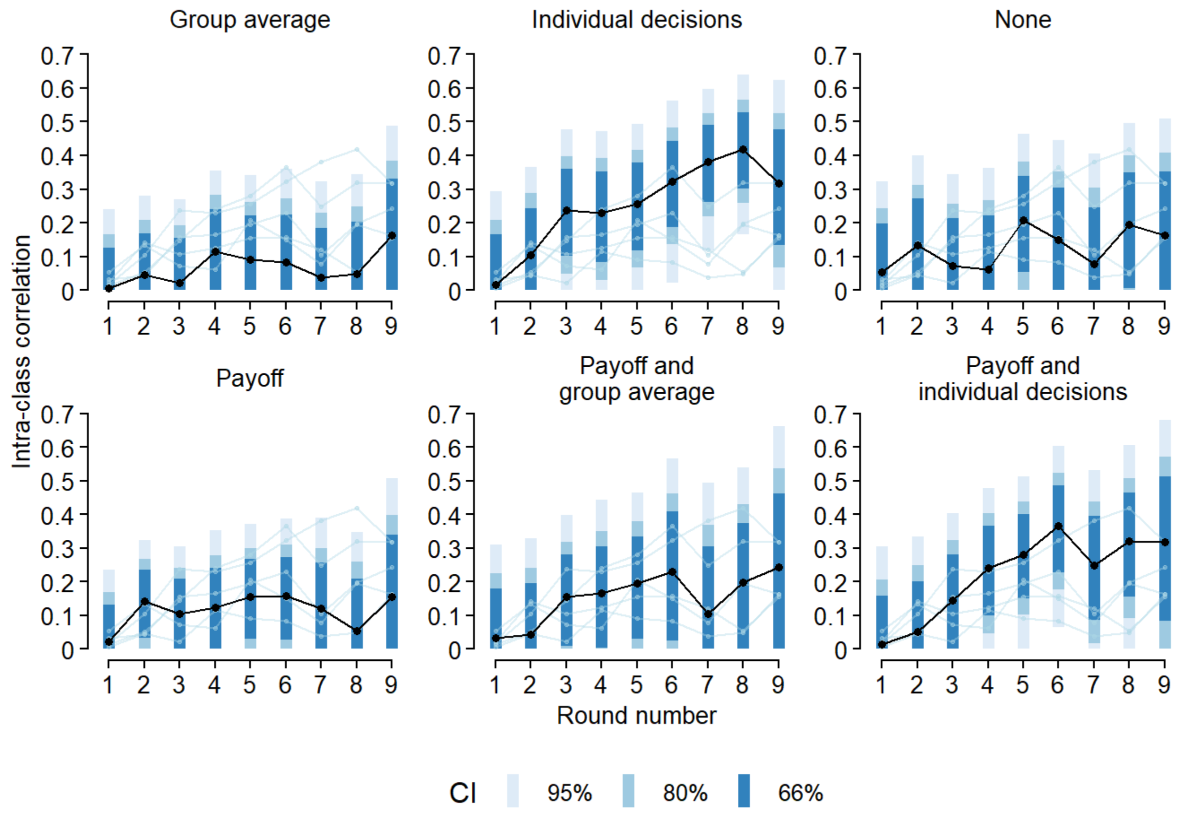

Fig. S4. Intra-class correlation across rounds of play in each experimental condition.

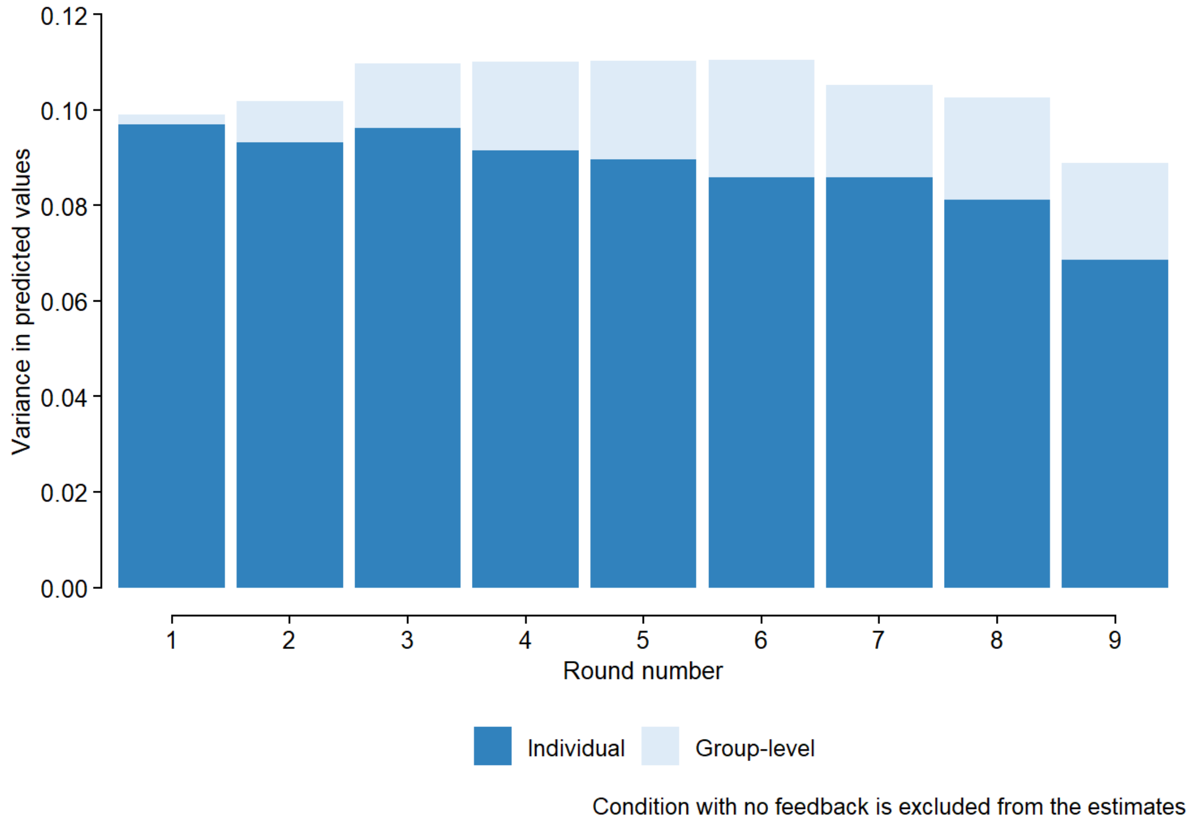

Fig. S5. Variance partitions across rounds of play.

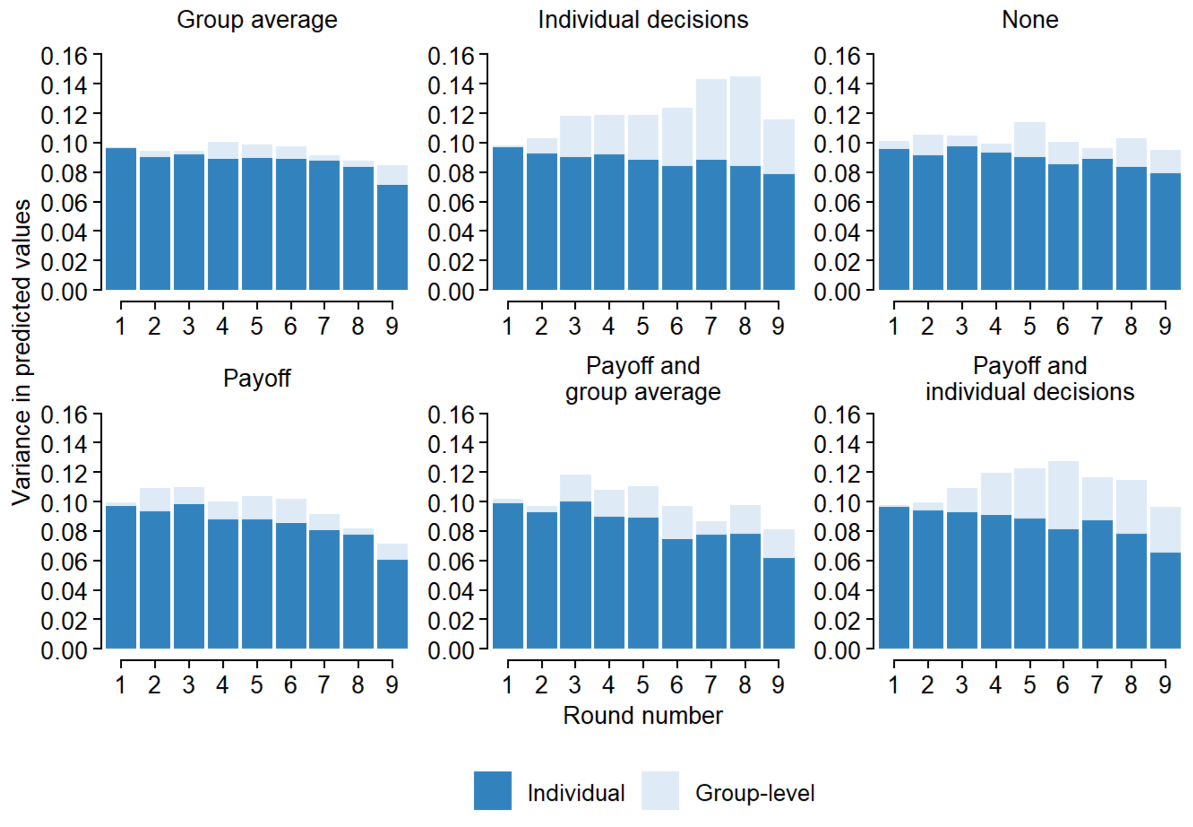

Fig. S6. Variance partitions across rounds of play in each experimental condition.

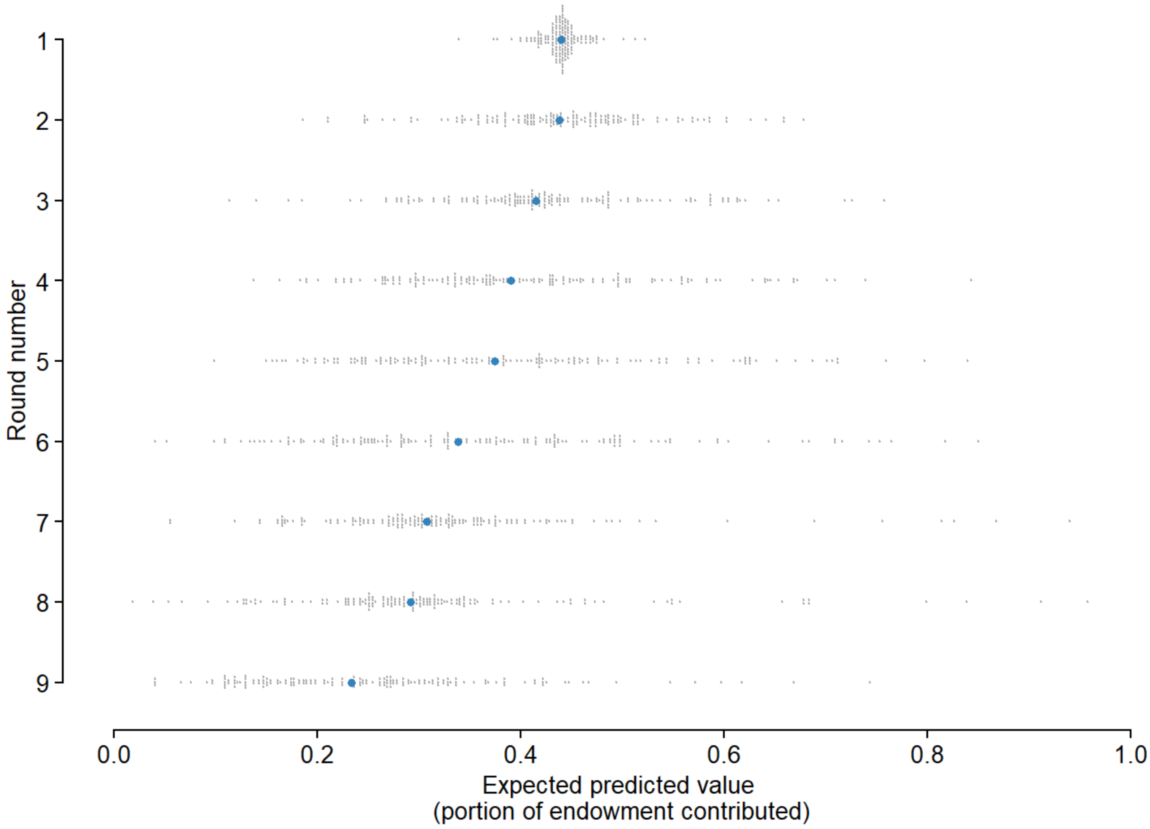

Fig. S7. Overall (blue dot) and group-level (grey dots) predictions across rounds of play.

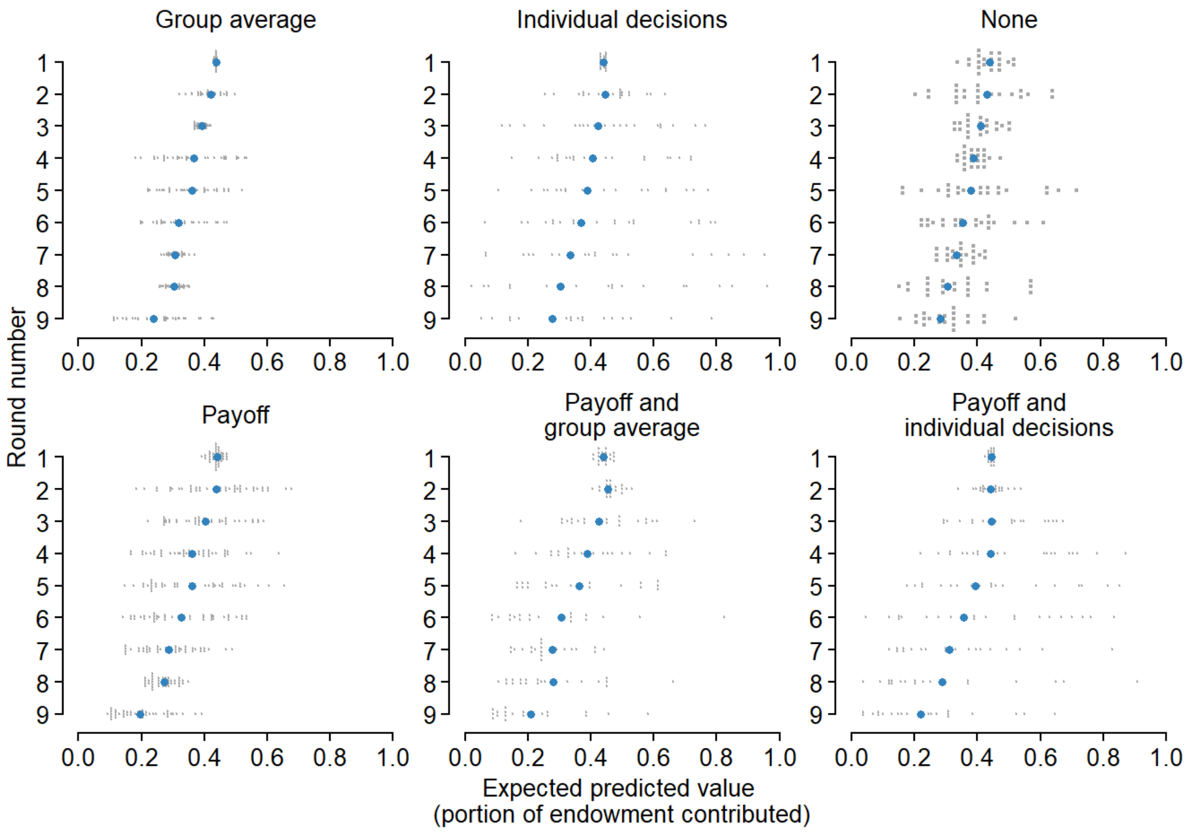

Fig. S8. Overall (blue dot) and group-level (grey dots) predictions across rounds of play in each experimental condition.

Supplementary Figures for Diederich et al. (2016)

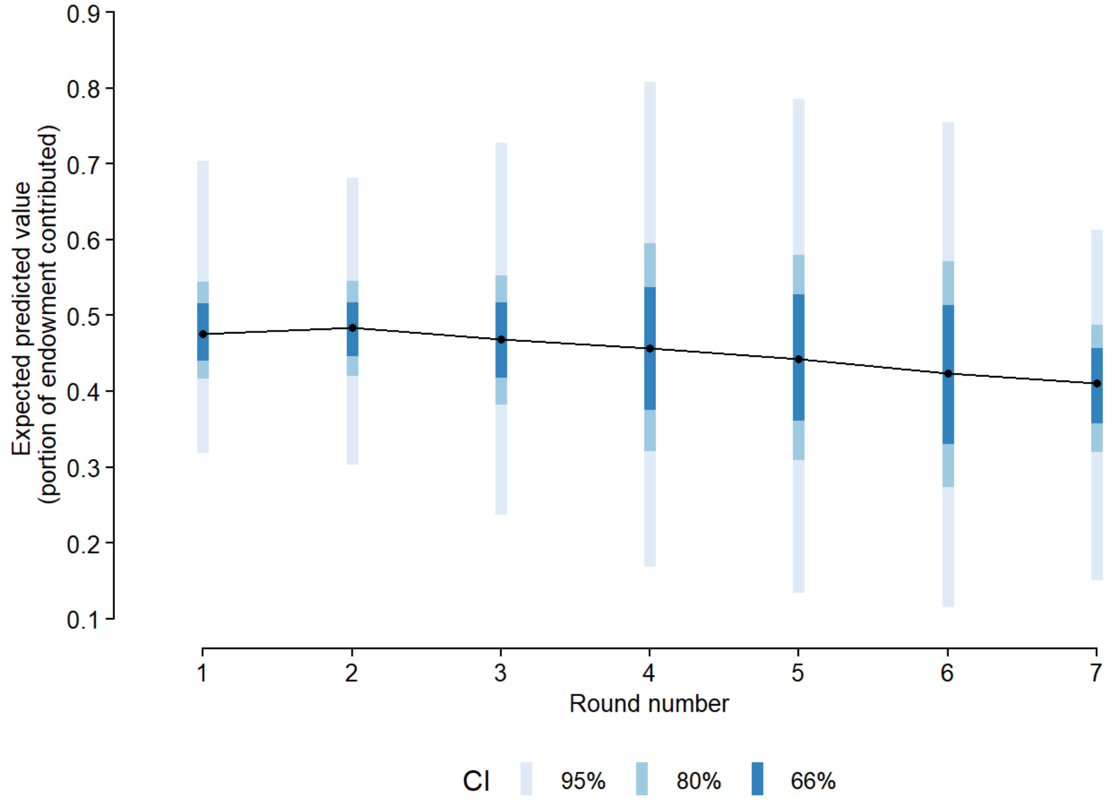

Fig. S9. Predicted level of cooperation across rounds of play.

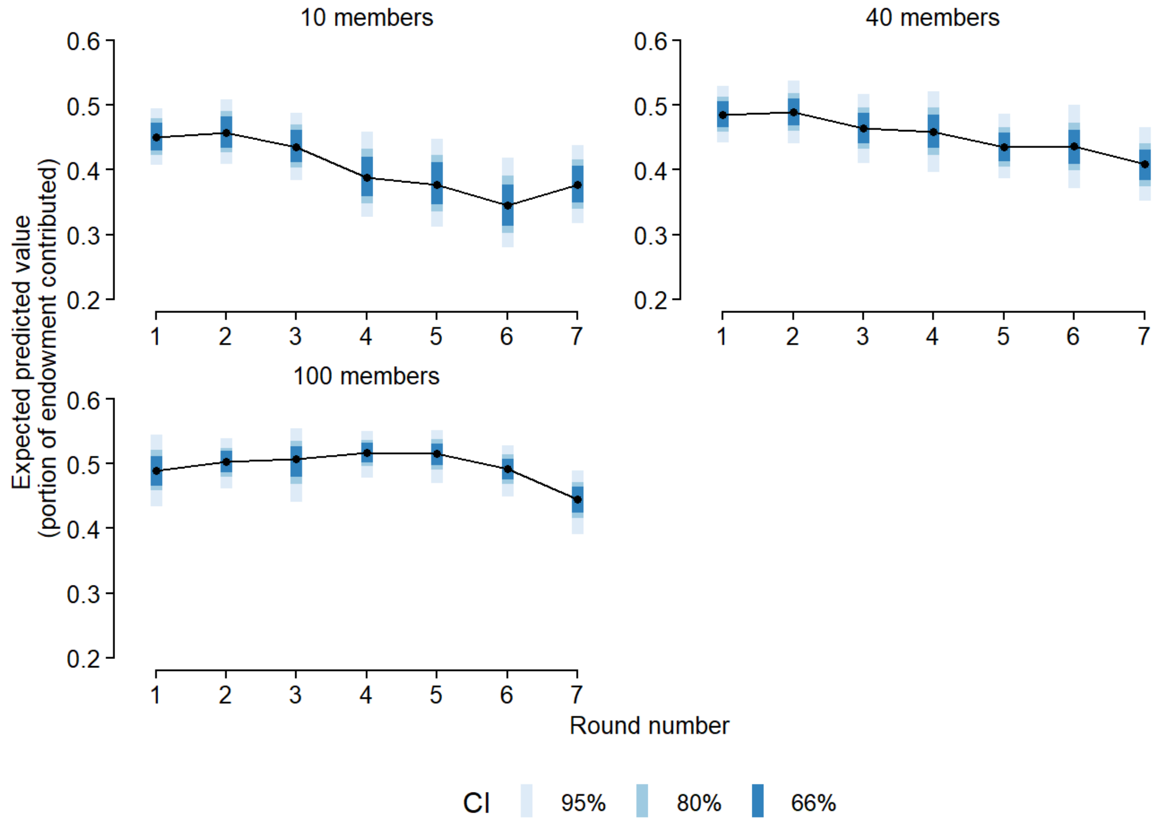

Fig. S10. Predicted level of cooperation across rounds of play in each experimental condition.

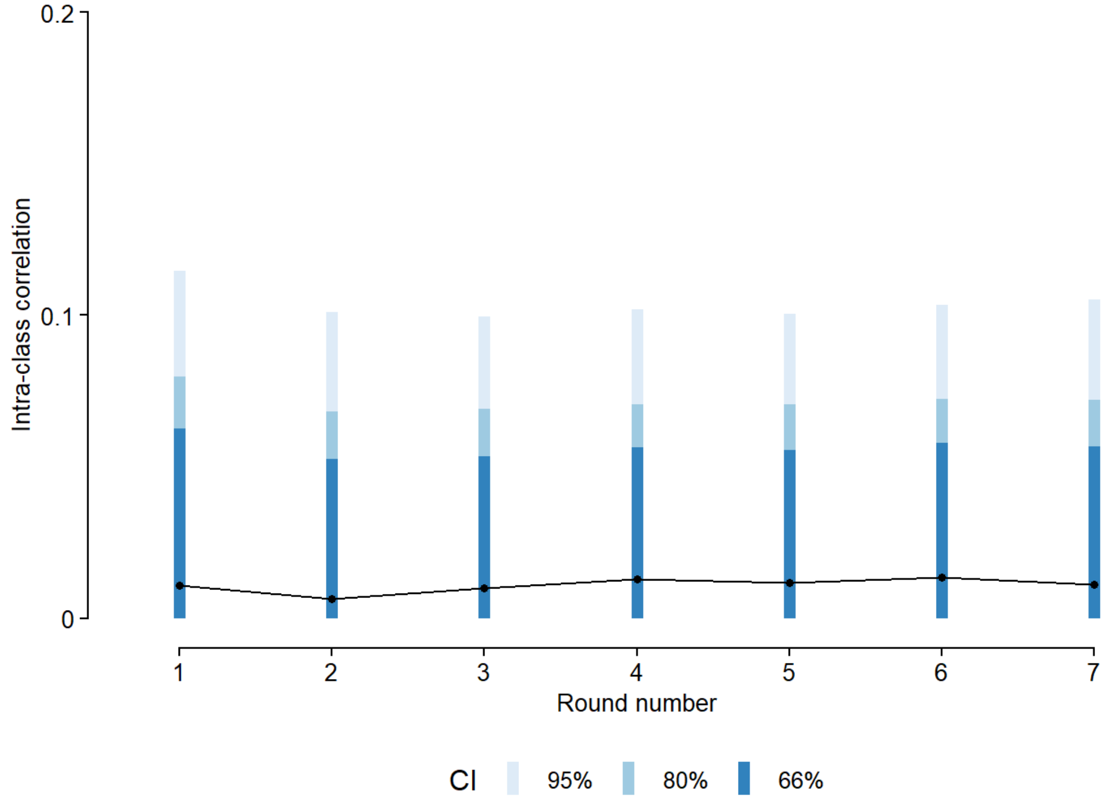

Fig. S11. Intra-class correlation across rounds of play.

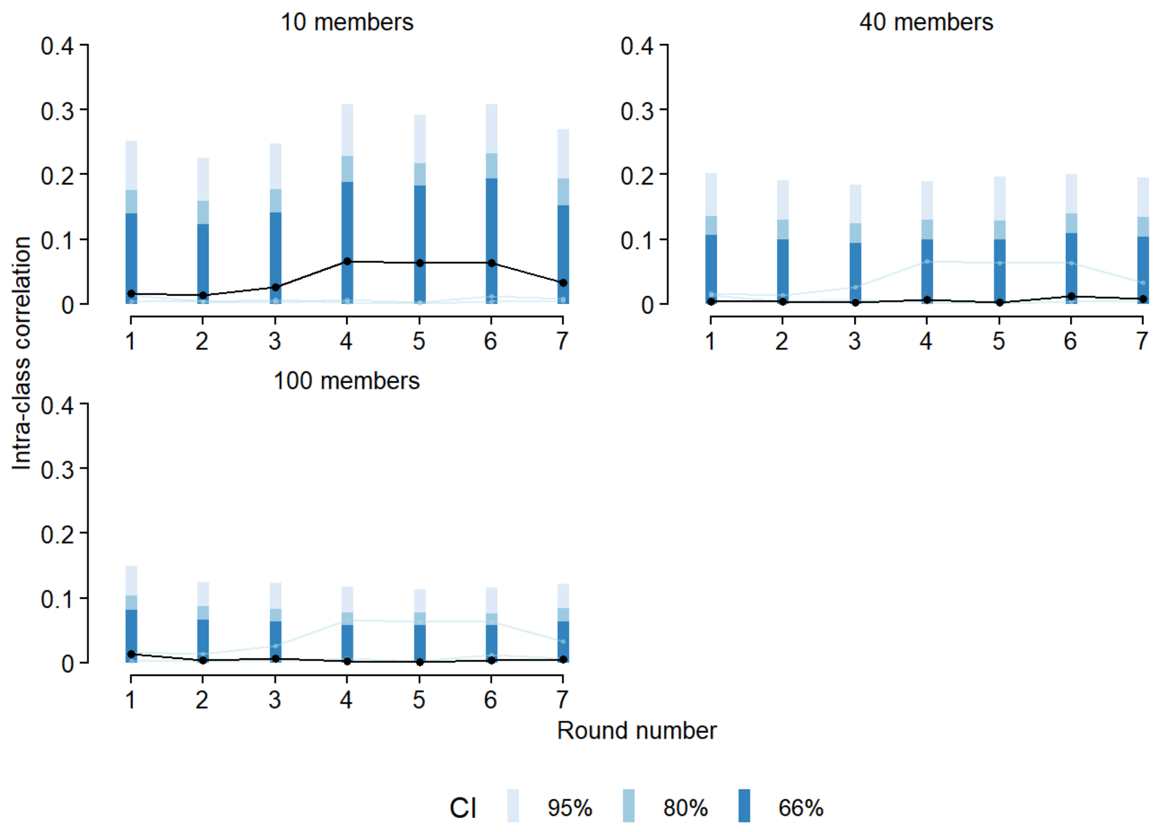

Fig. S12. Intra-class correlation across rounds of play in each experimental condition.

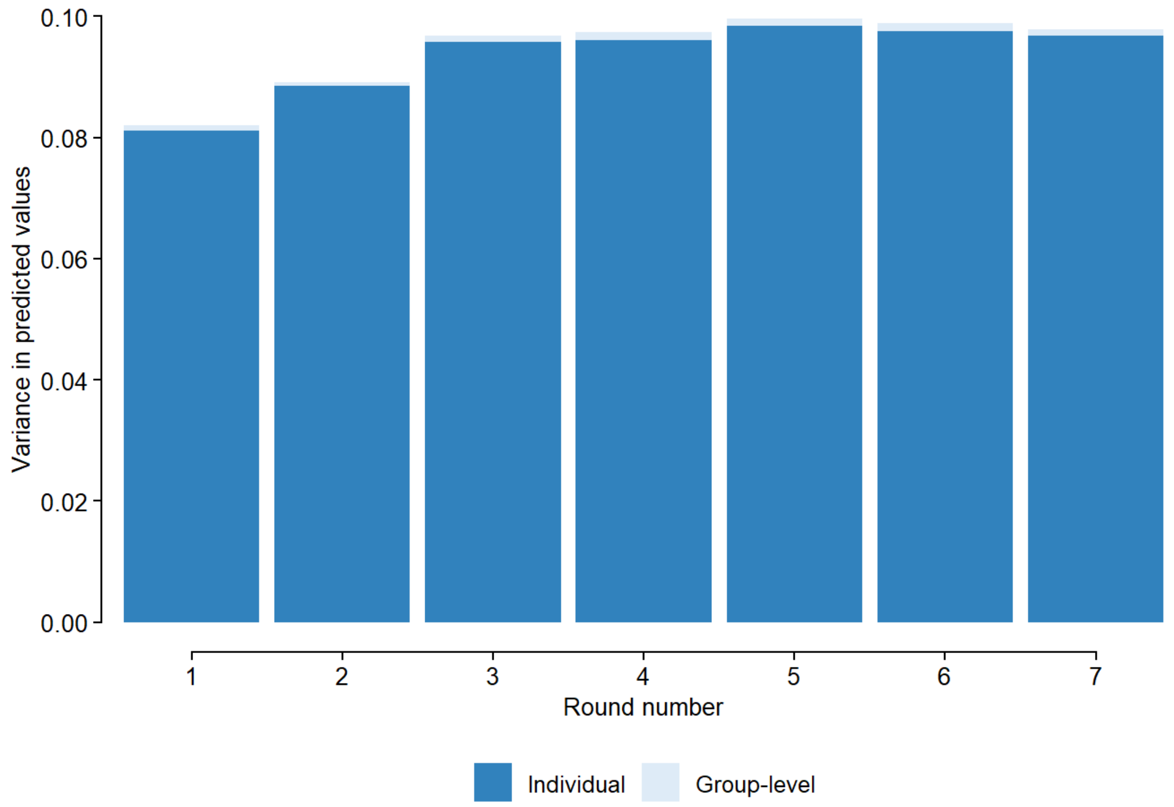

Fig. S13. Variance partitions across rounds of play.

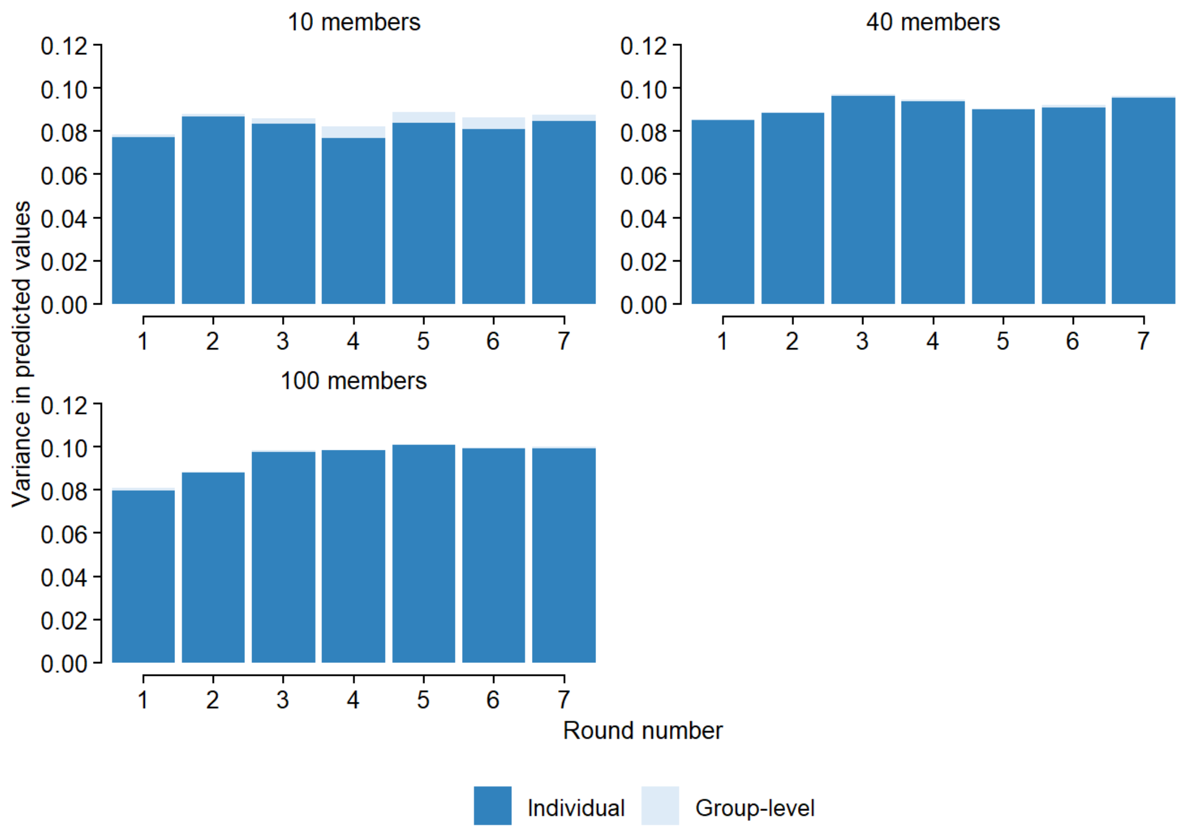

Fig. S14. Variance partitions across rounds of play in each experimental condition.

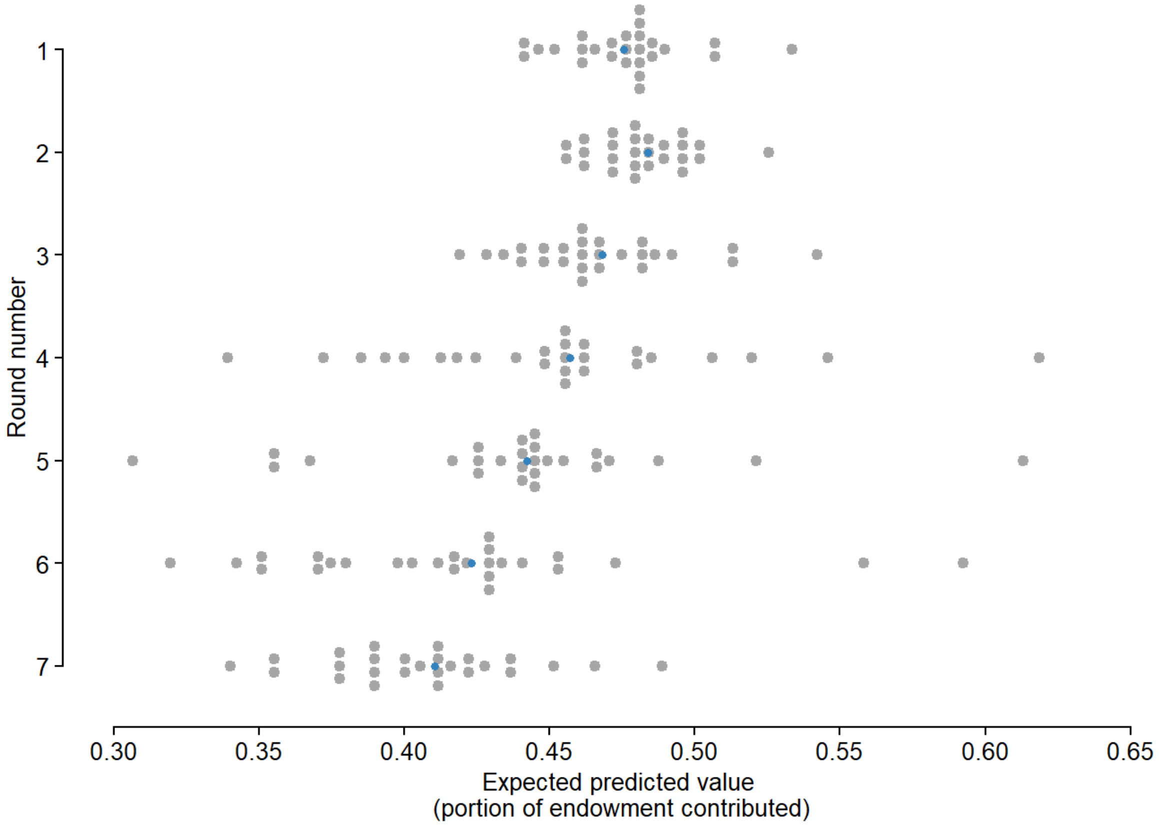

Fig. S15. Overall (blue dot) and group-level (grey dots) predictions across rounds of play.

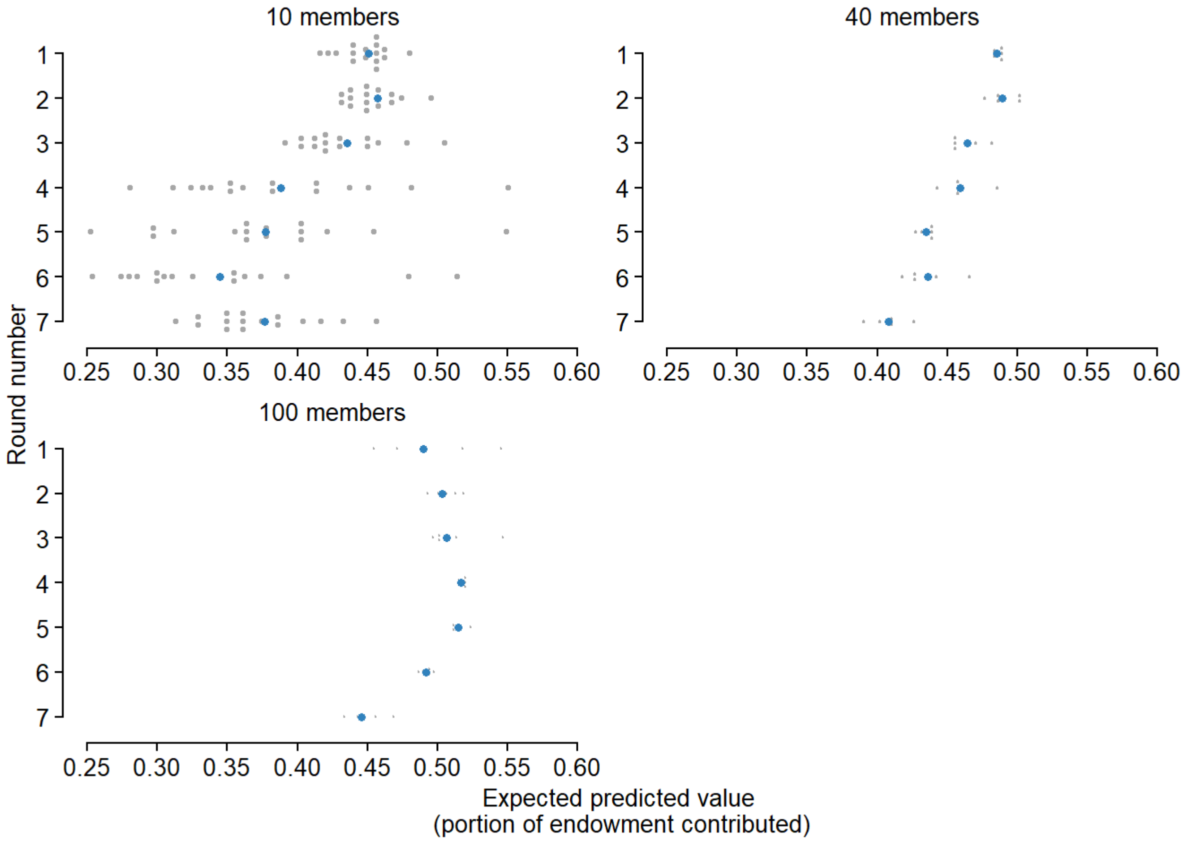

Fig. S16. Overall (blue dot) and group-level (grey dots) predictions across rounds of play in each experimental condition.

Supplementary Figures for Grandjean et al. (2022)

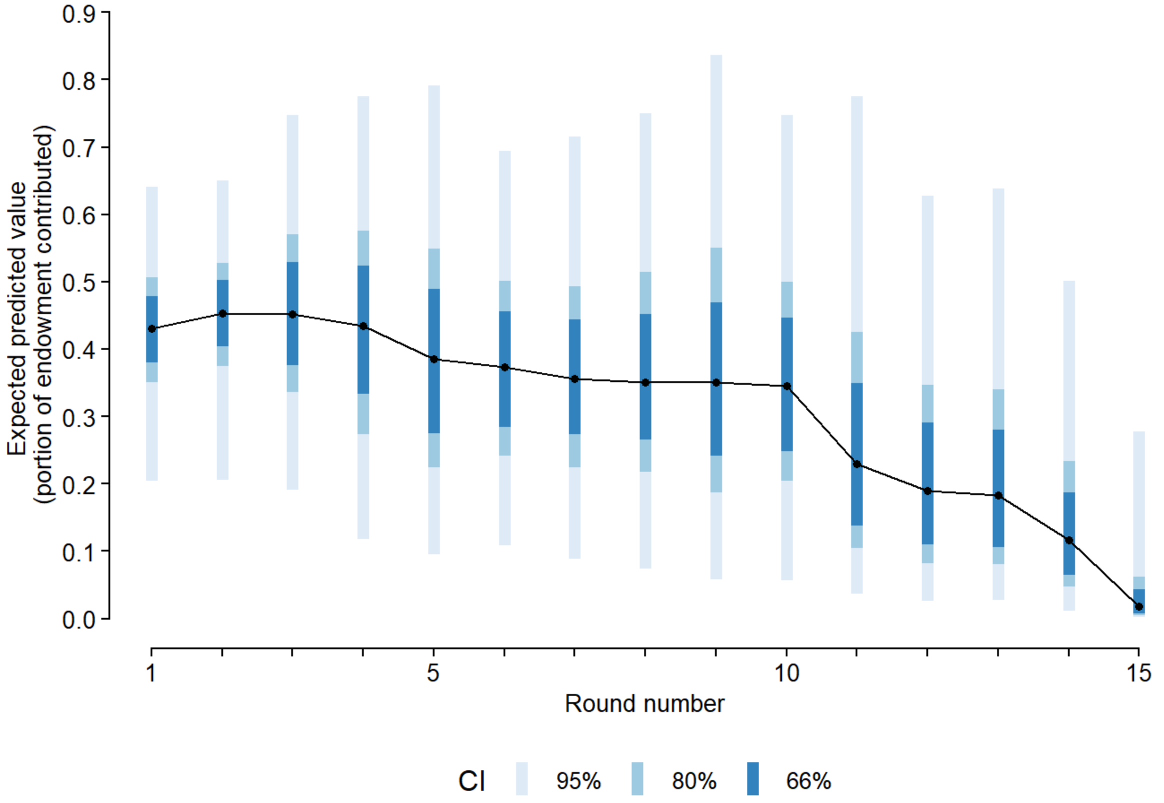

Fig. S17. Predicted level of cooperation across rounds of play.

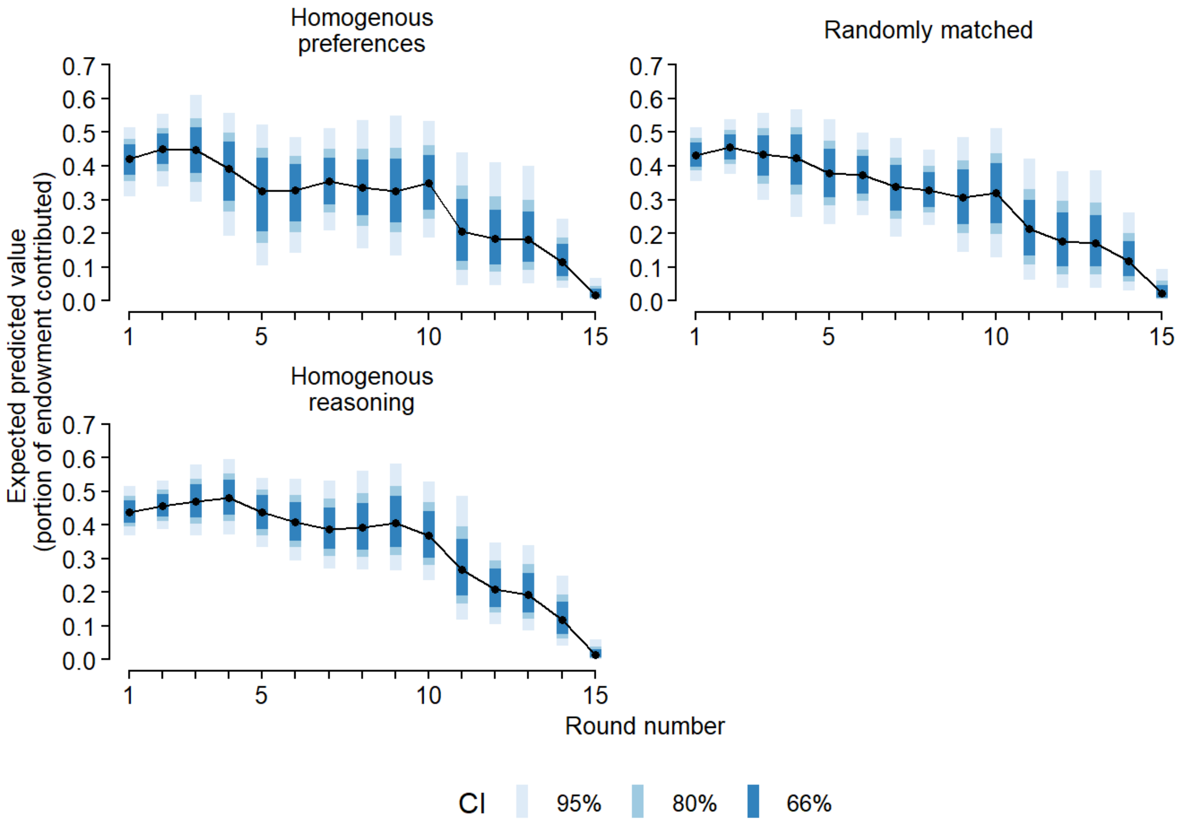

Fig. S18. Predicted level of cooperation across rounds of play in each experimental condition.

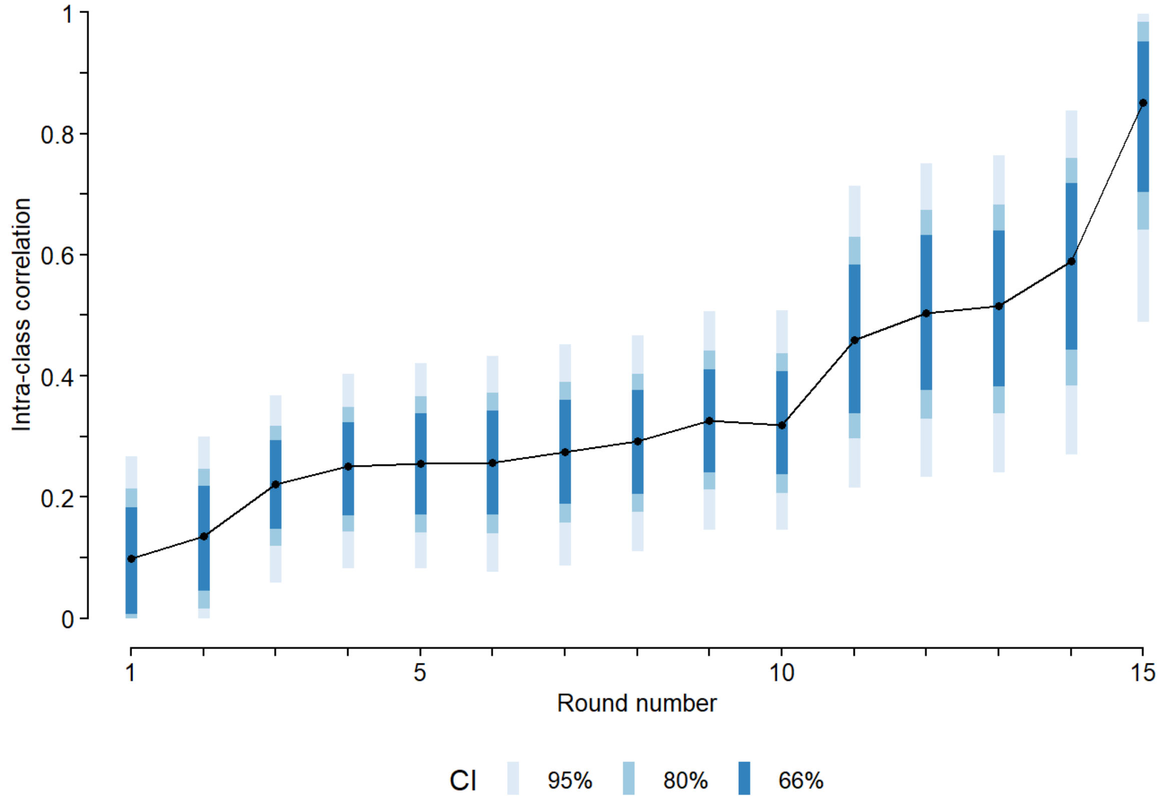

Fig. S19. Intra-class correlation across rounds of play.

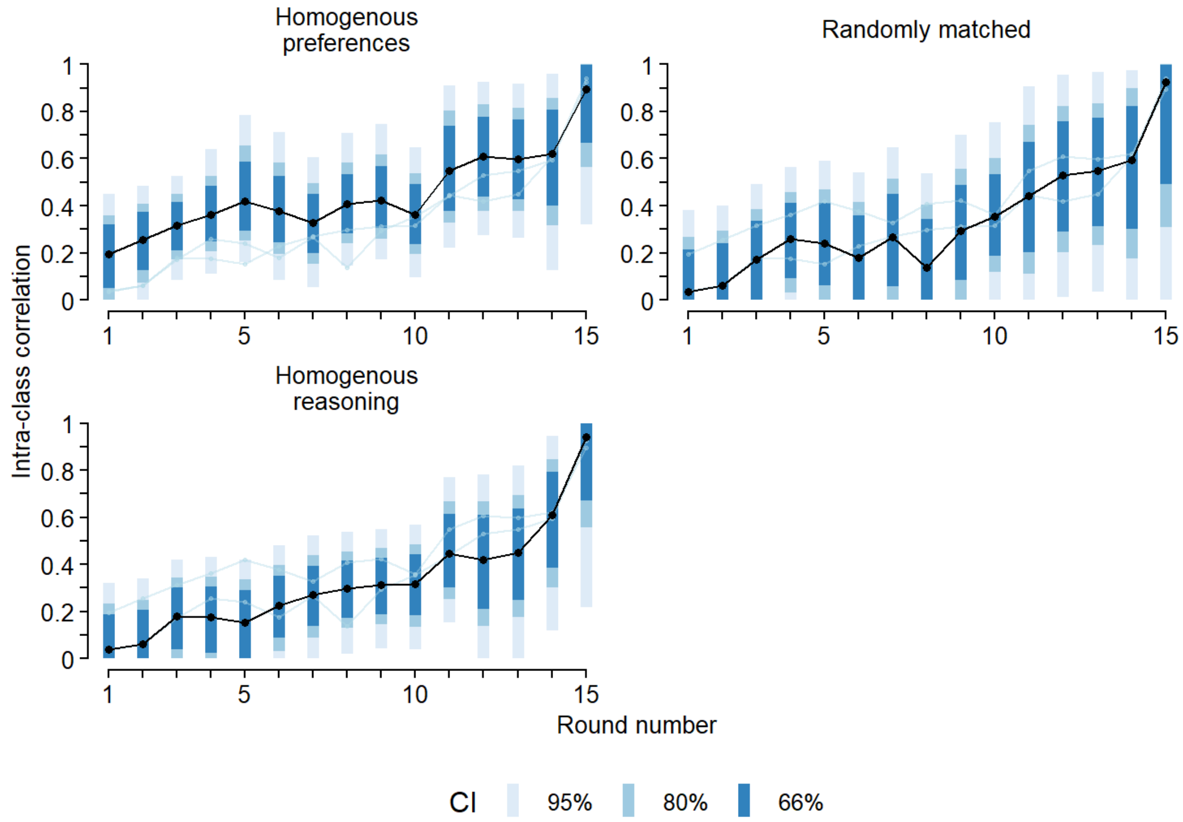

Fig. S20. Intra-class correlation across rounds of play in each experimental condition.

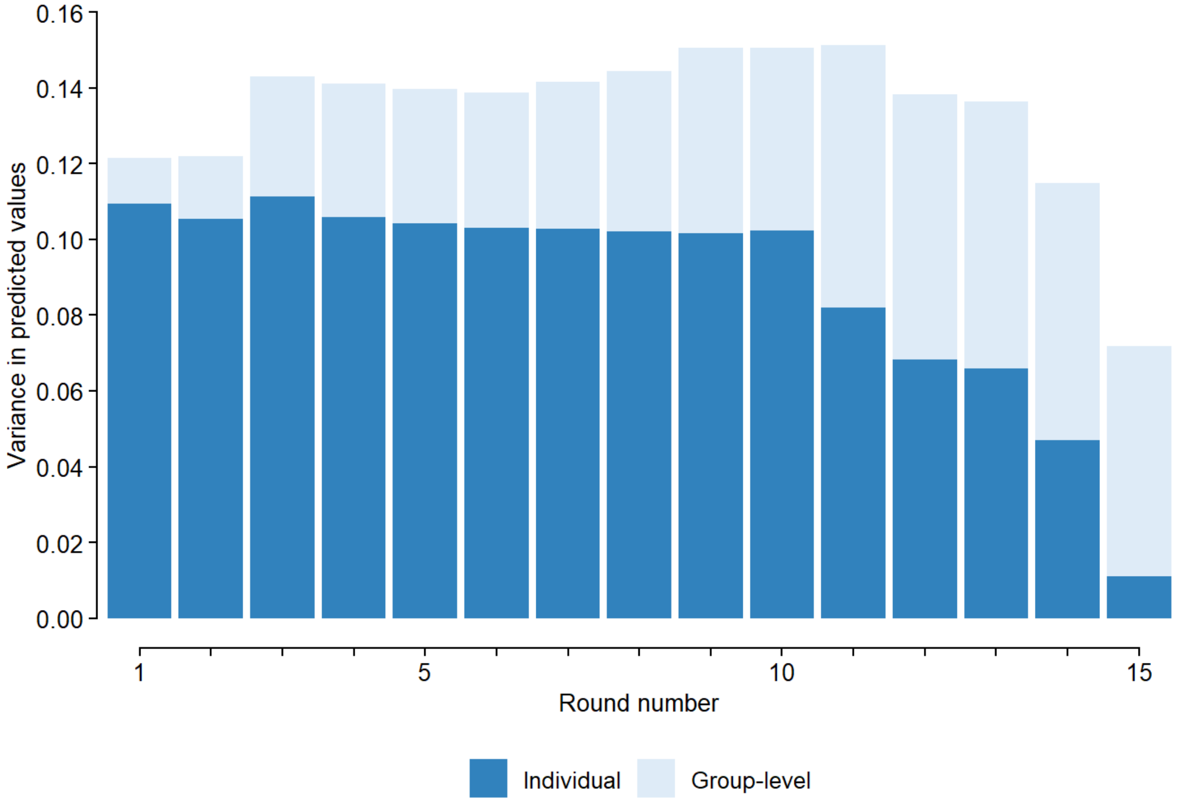

Fig. S21. Variance partitions across rounds of play.

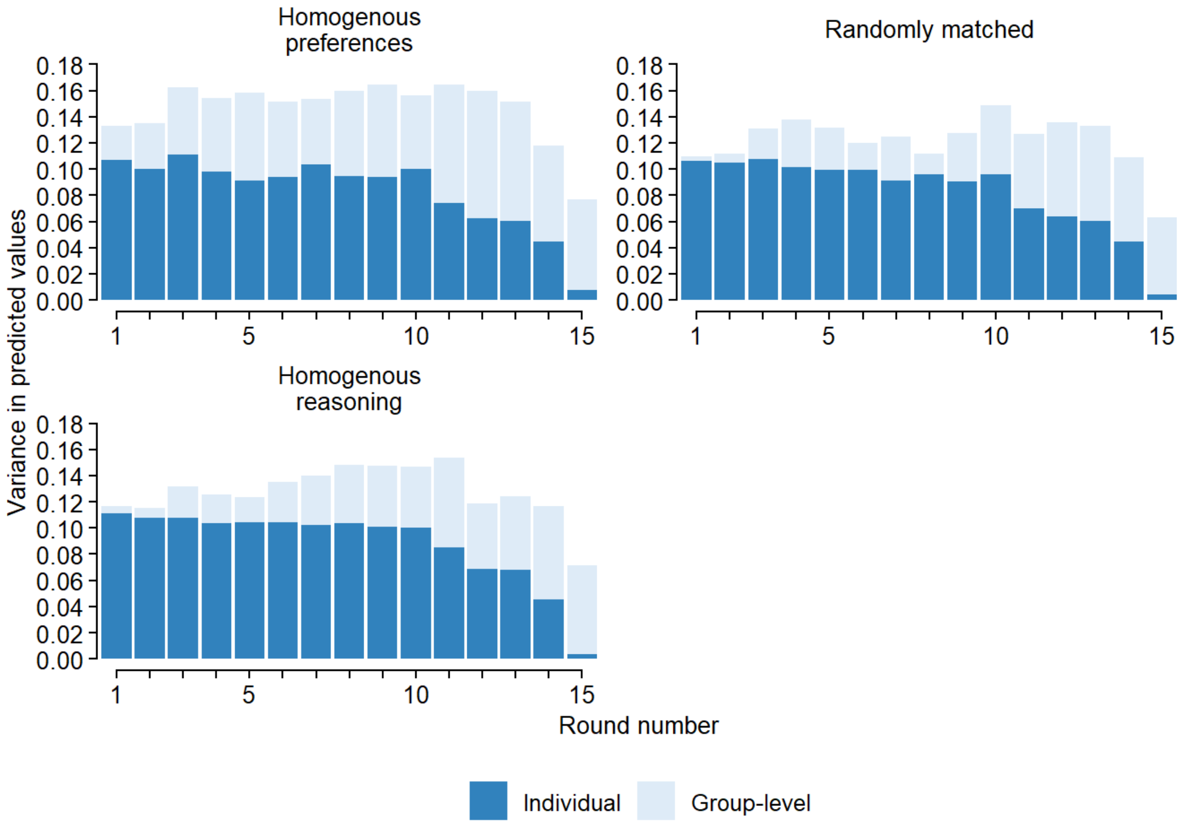

Fig. S22. Variance partitions across rounds of play in each experimental condition.

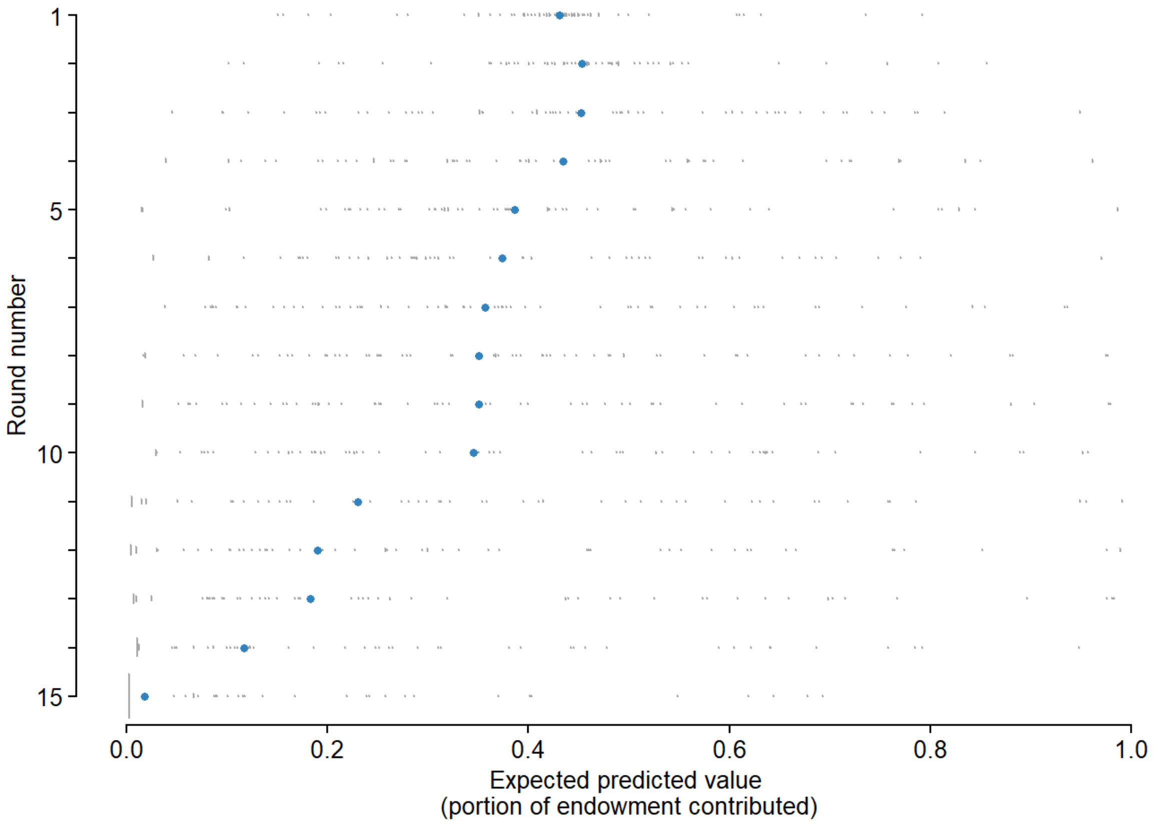

Fig. S23. Overall (blue dot) and group-level (grey dots) predictions across rounds of play.

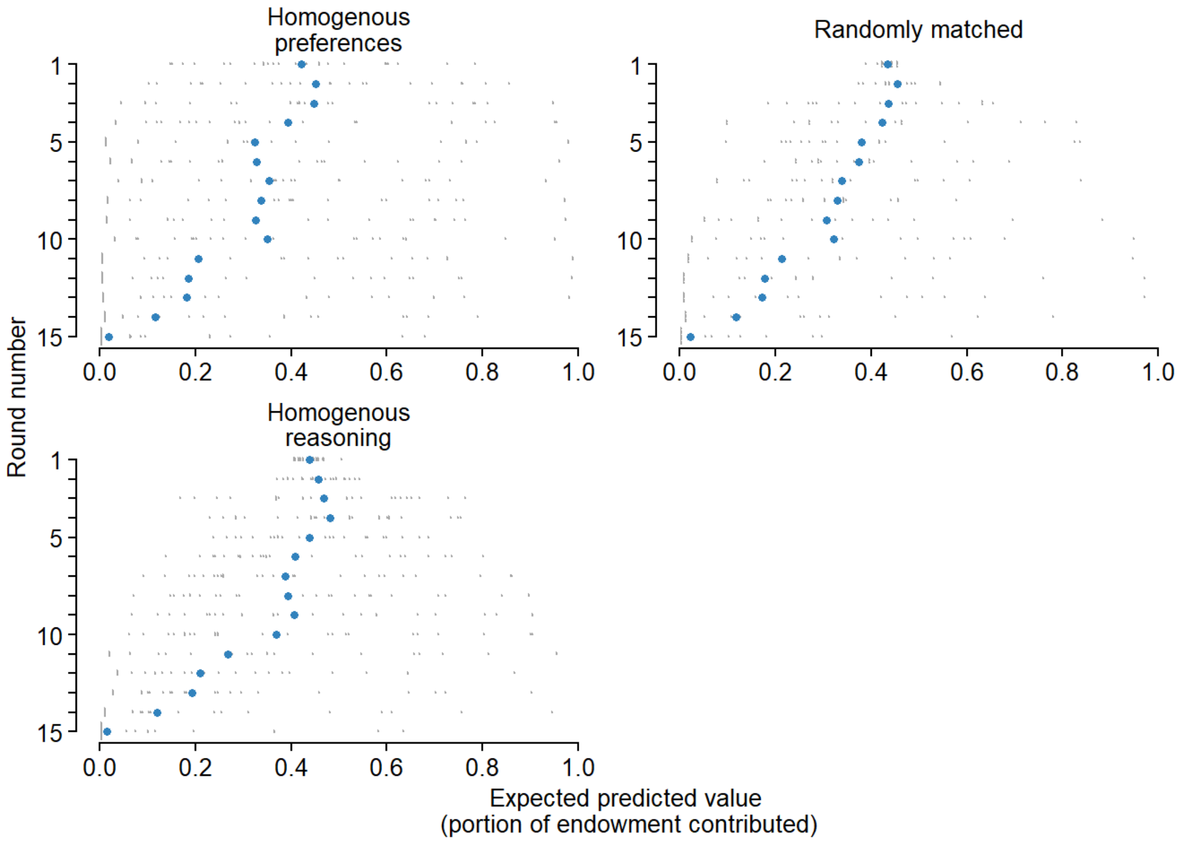

Fig. S24. Overall (blue dot) and group-level (grey dots) predictions across rounds of play in each experimental condition.

Supplementary Figures for Gächter et al. (2017)

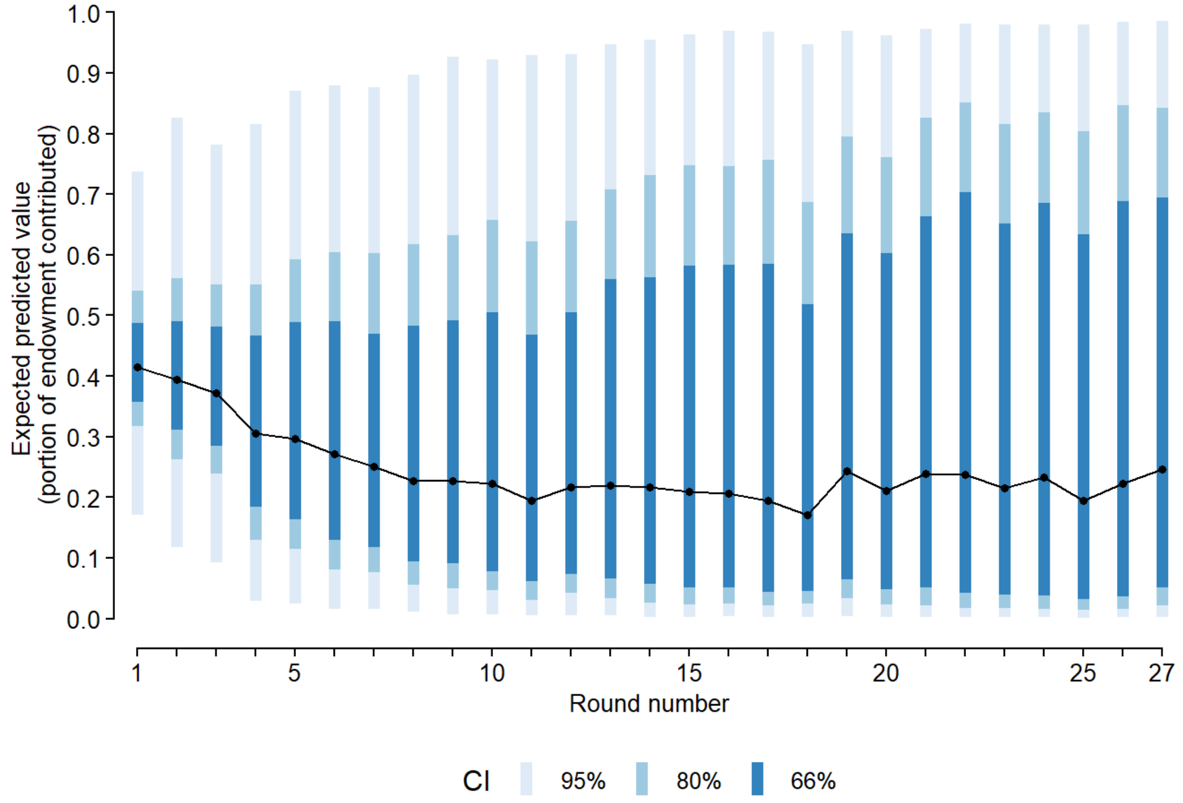

Fig. S25. Predicted level of cooperation across rounds of play.

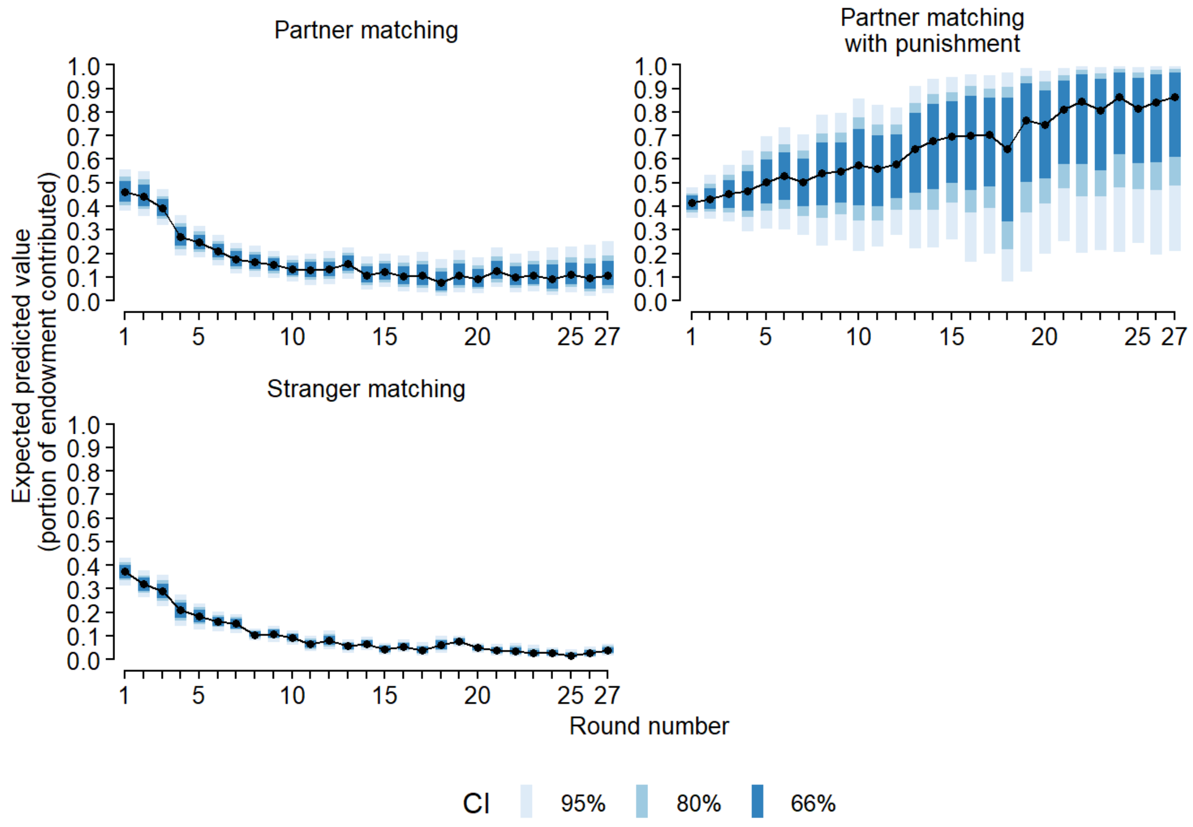

Fig. S26. Predicted level of cooperation across rounds of play in each experimental condition.

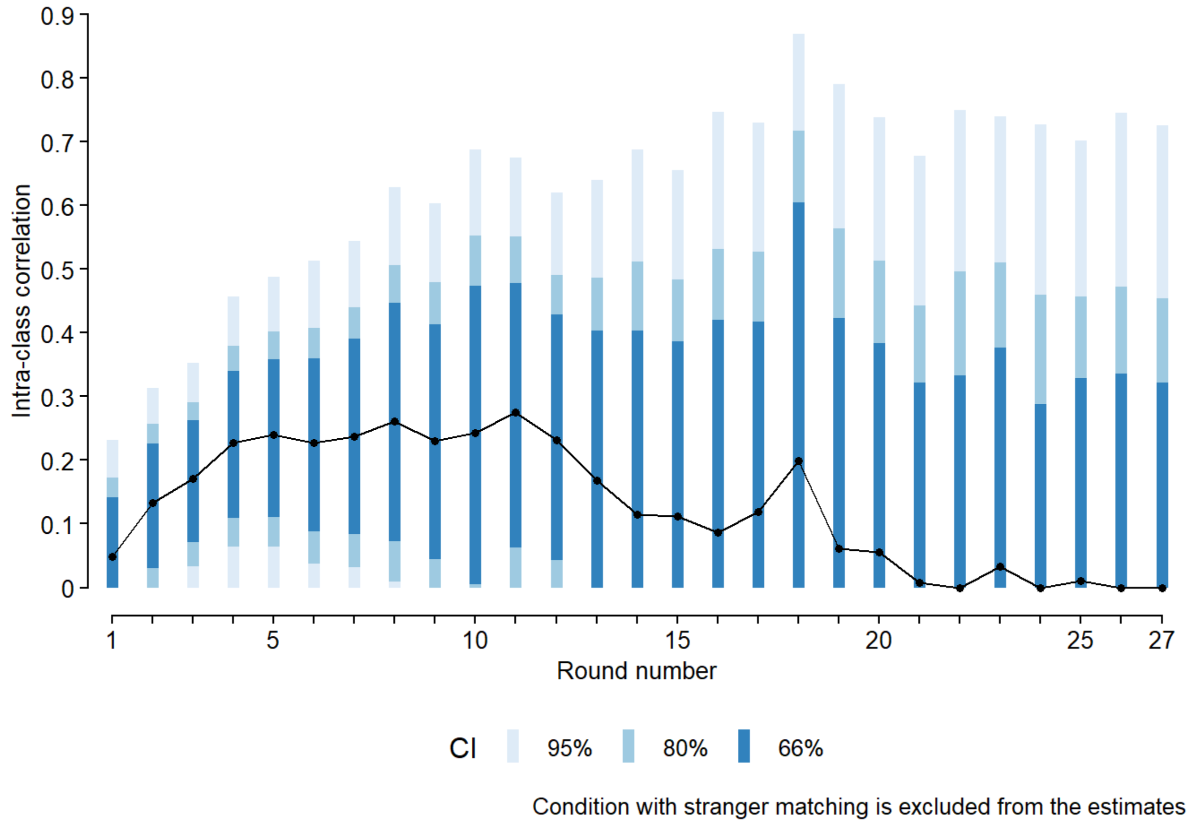

Fig. S27. Intra-class correlation across rounds of play.

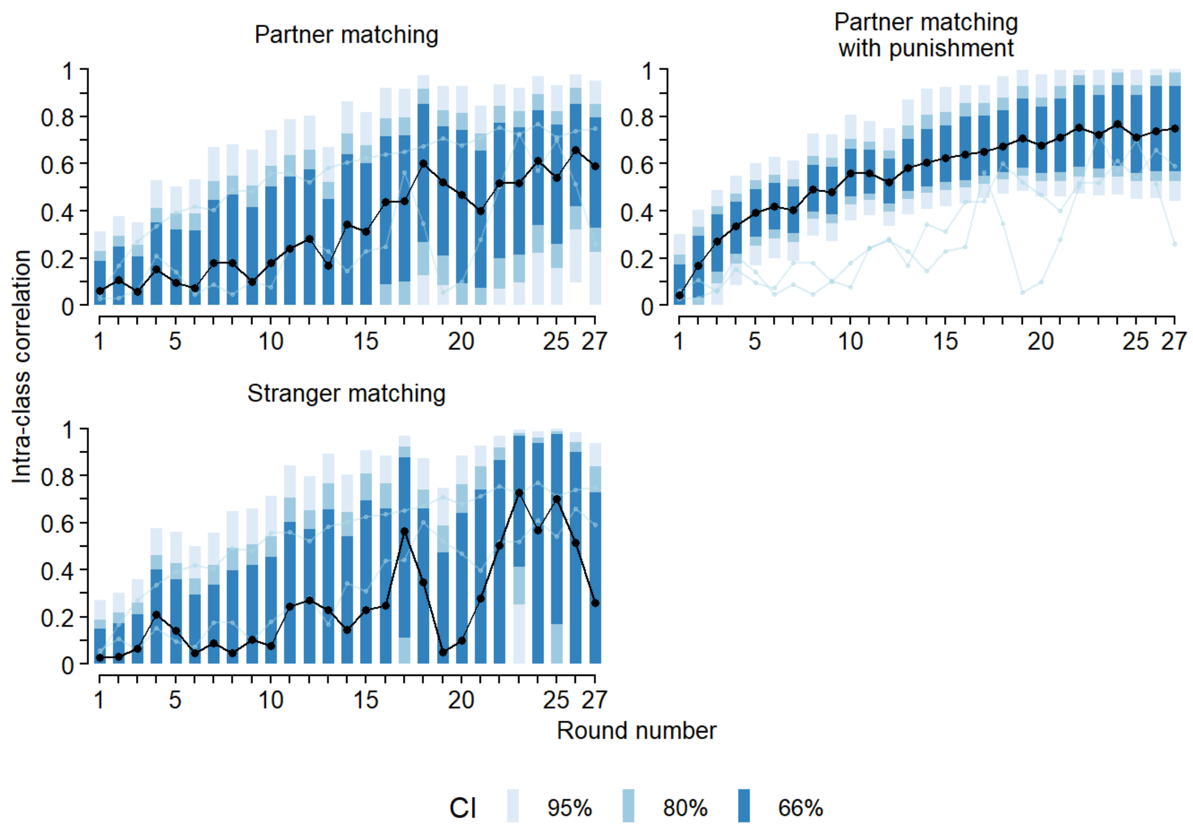

Fig. S28. Intra-class correlation across rounds of play in each experimental condition.

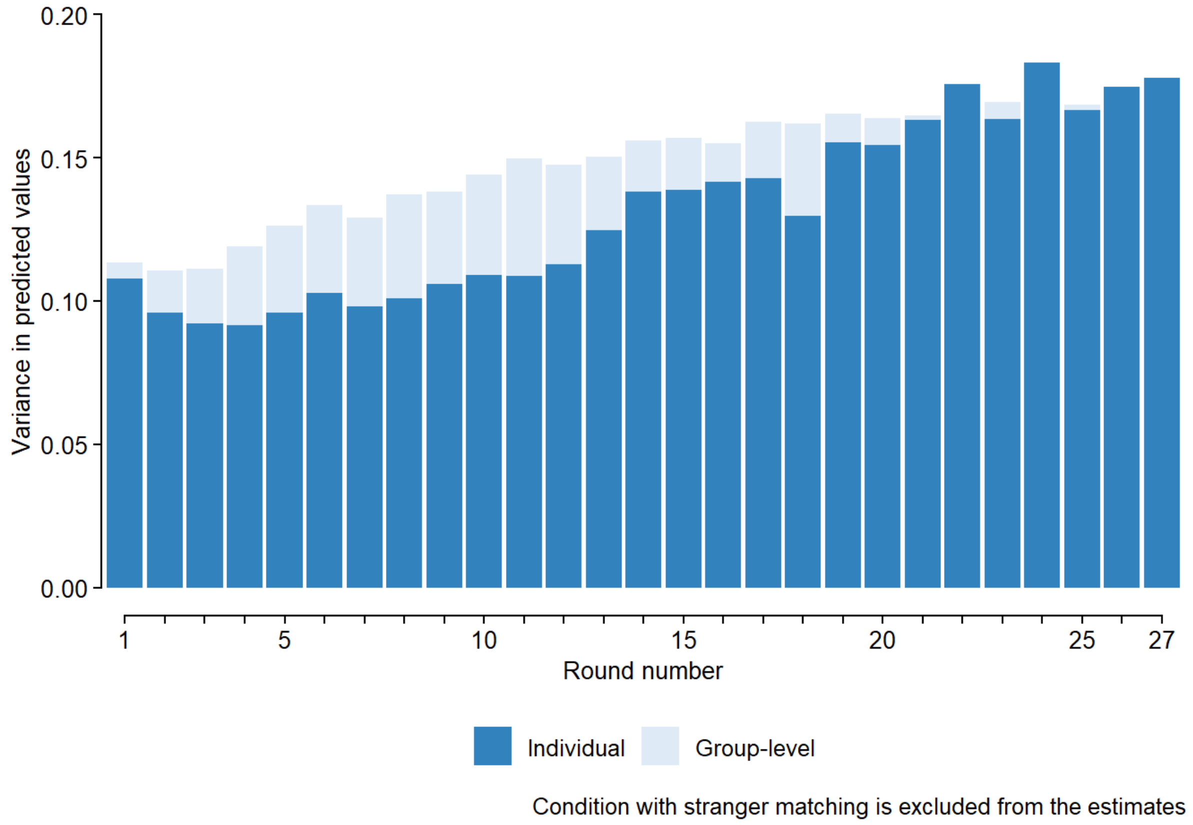

Fig. S29. Variance partitions across rounds of play.

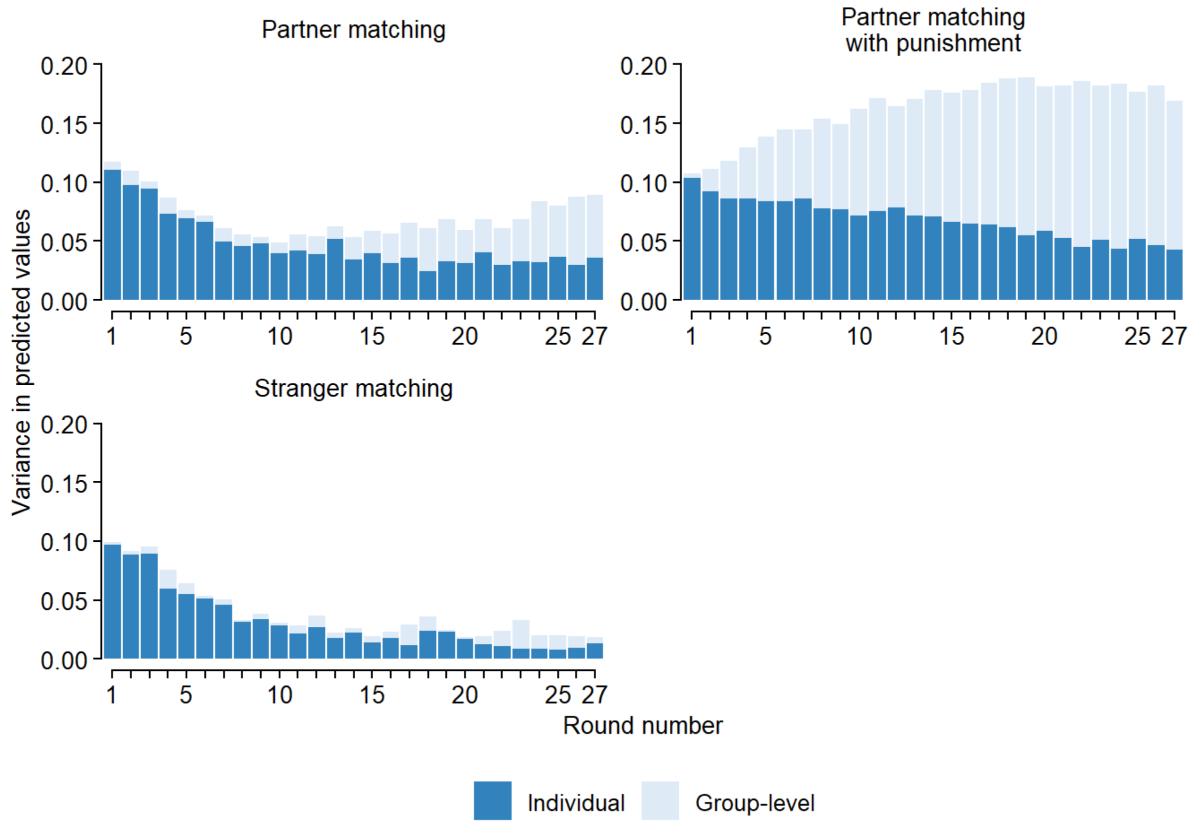

Fig. S30. Variance partitions across rounds of play in each experimental condition.

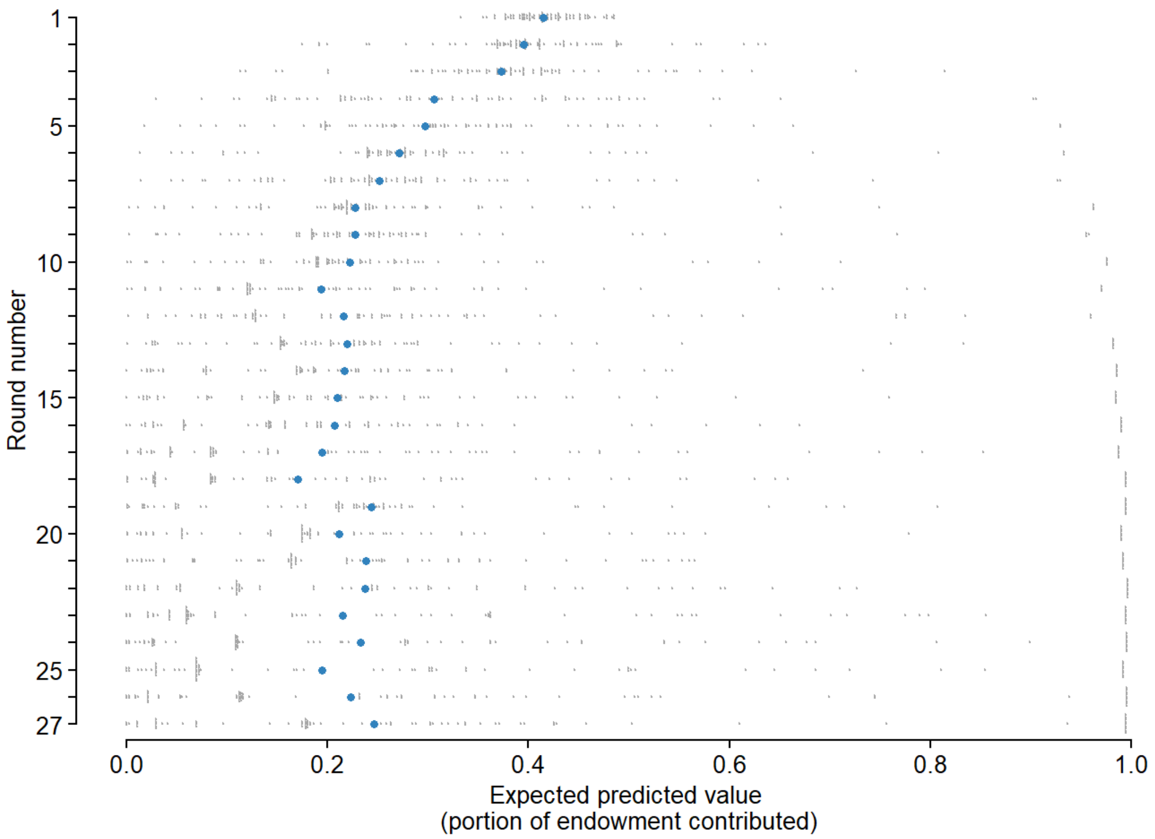

Fig. S31. Overall (blue dot) and group-level (grey dots) predictions across rounds of play.

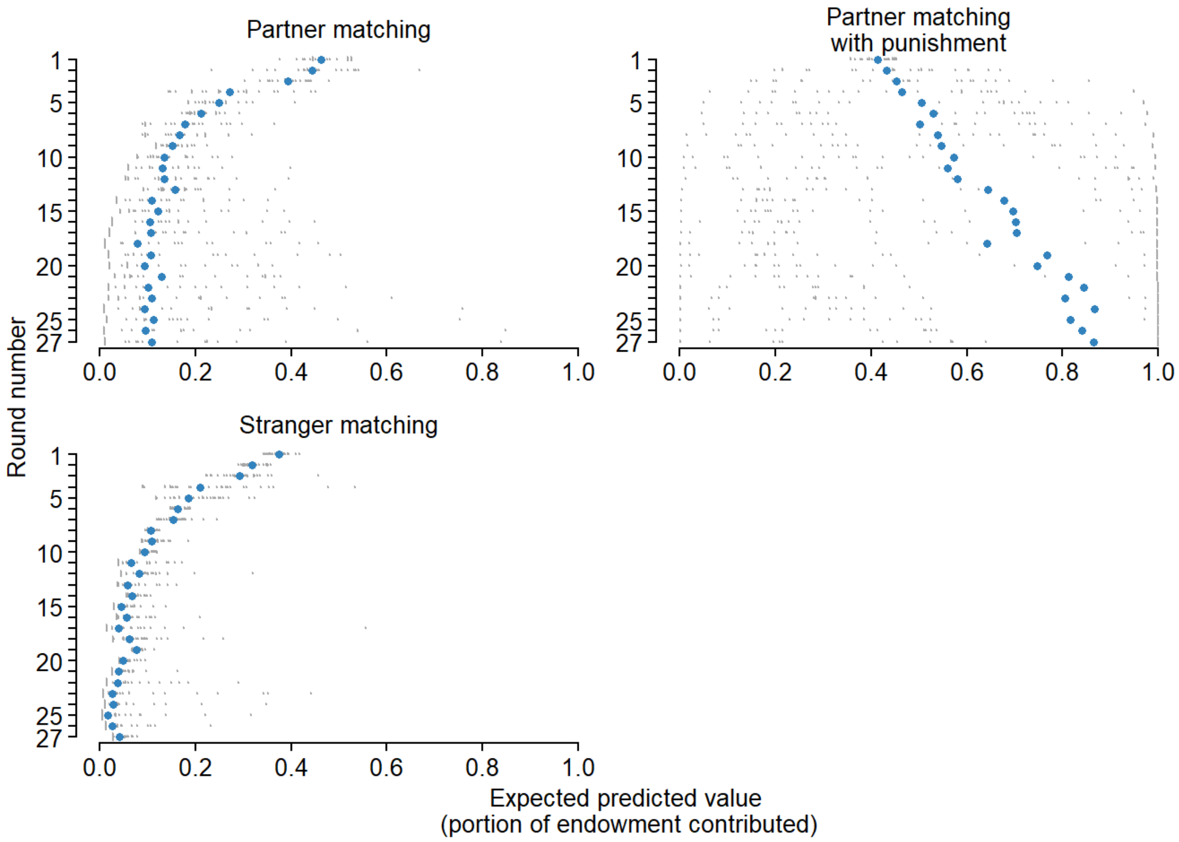

Fig. S32. Overall (blue dot) and group-level (grey dots) predictions across rounds of play in each experimental condition.

Supplementary Figures for Nosenzo et al. (2015)

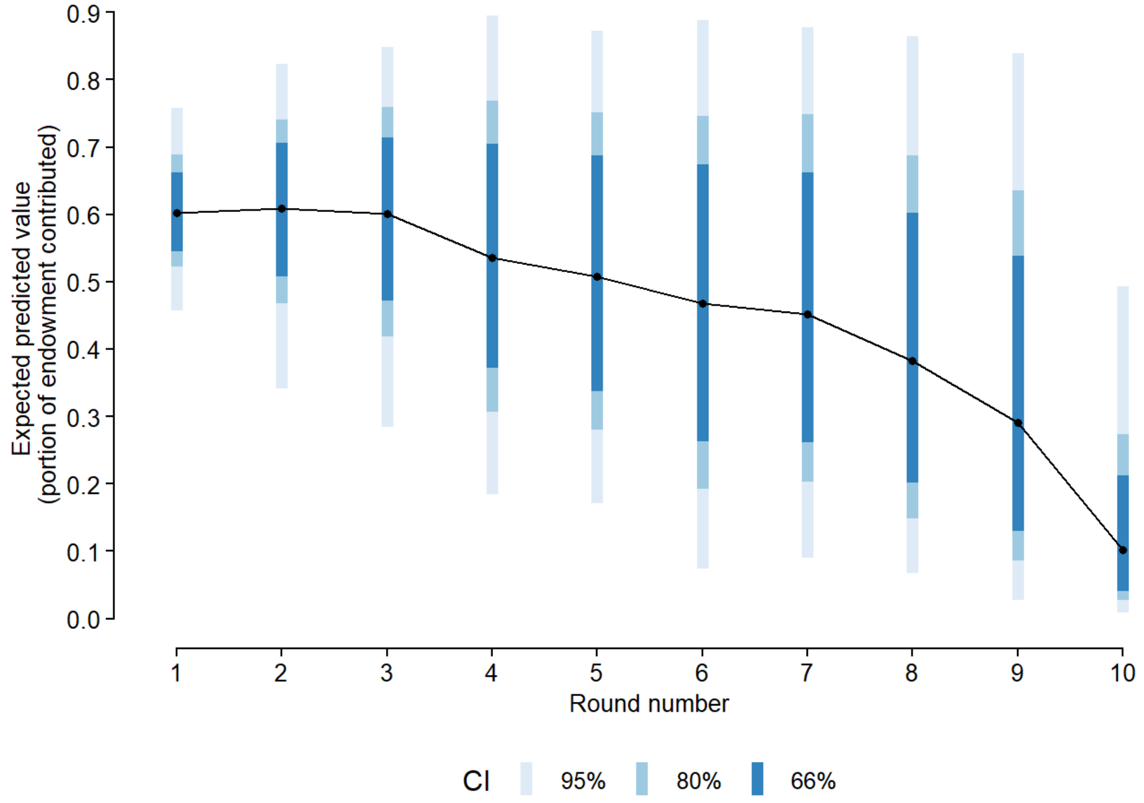

Fig. S33. Predicted level of cooperation across rounds of play.

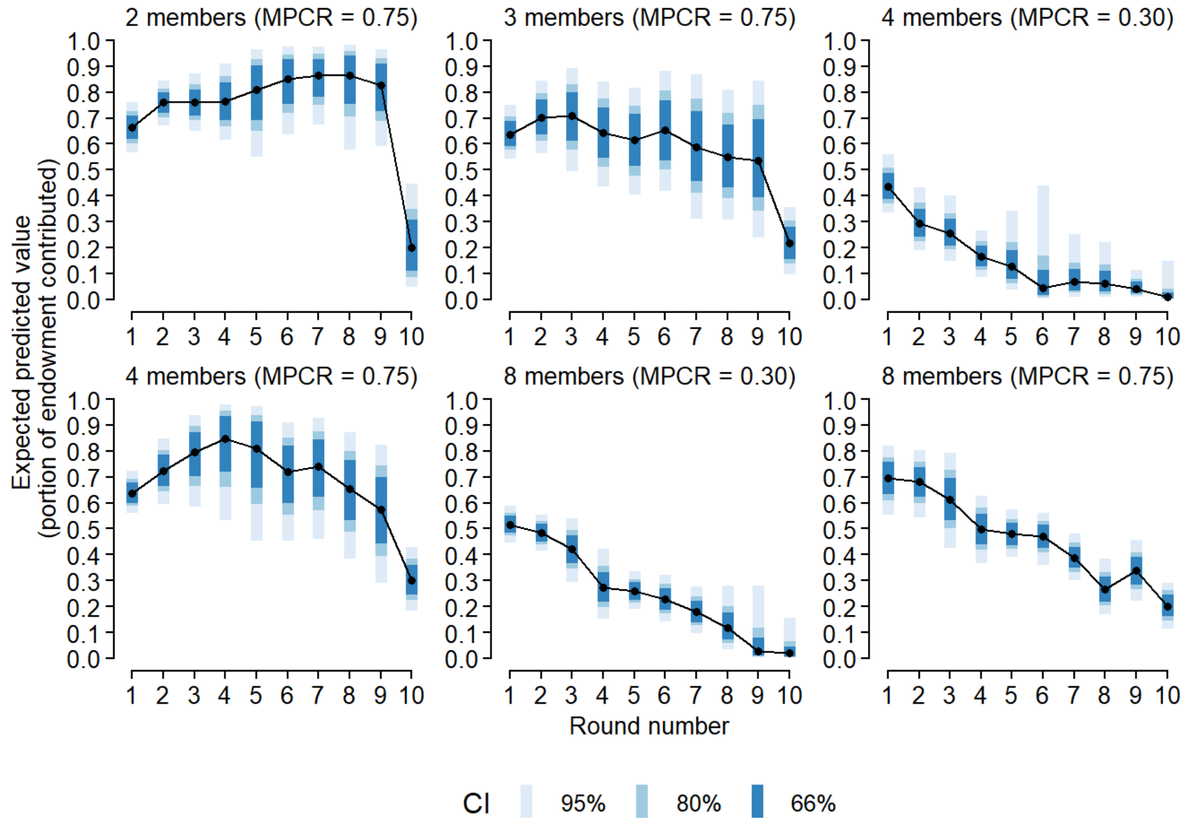

Fig. S34. Predicted level of cooperation across rounds of play in each experimental condition.

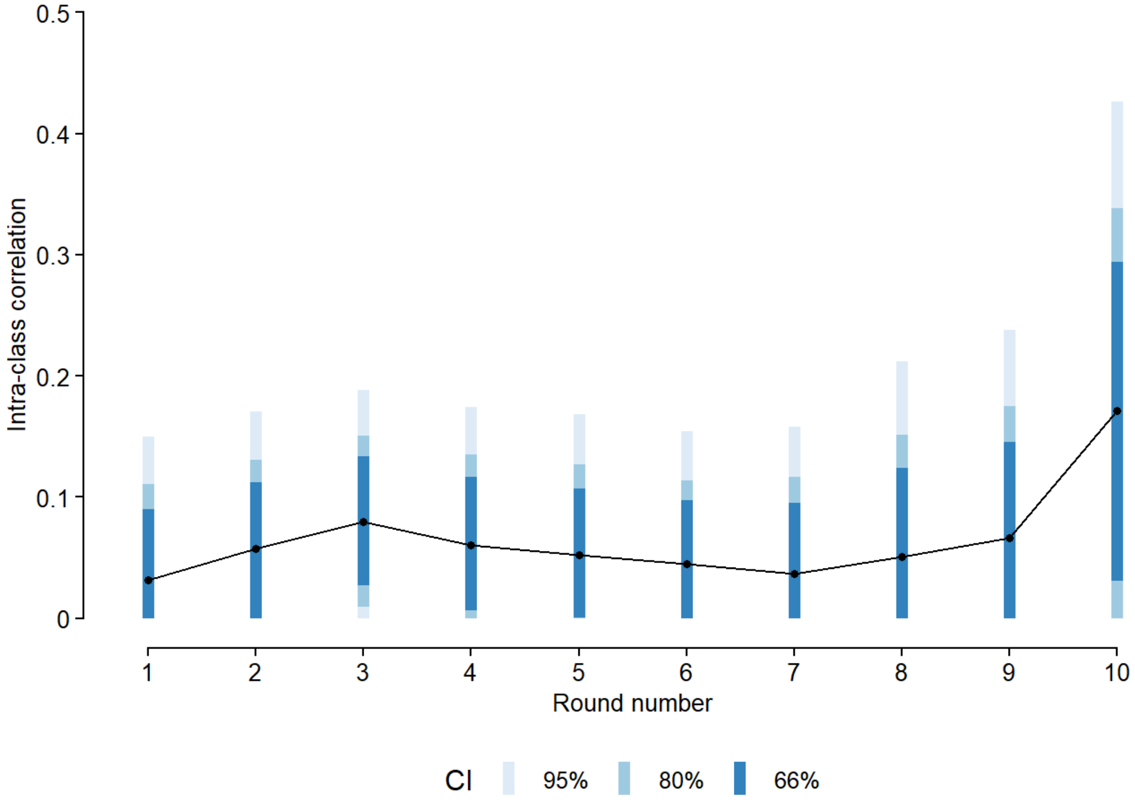

Fig. S35. Intra-class correlation across rounds of play.

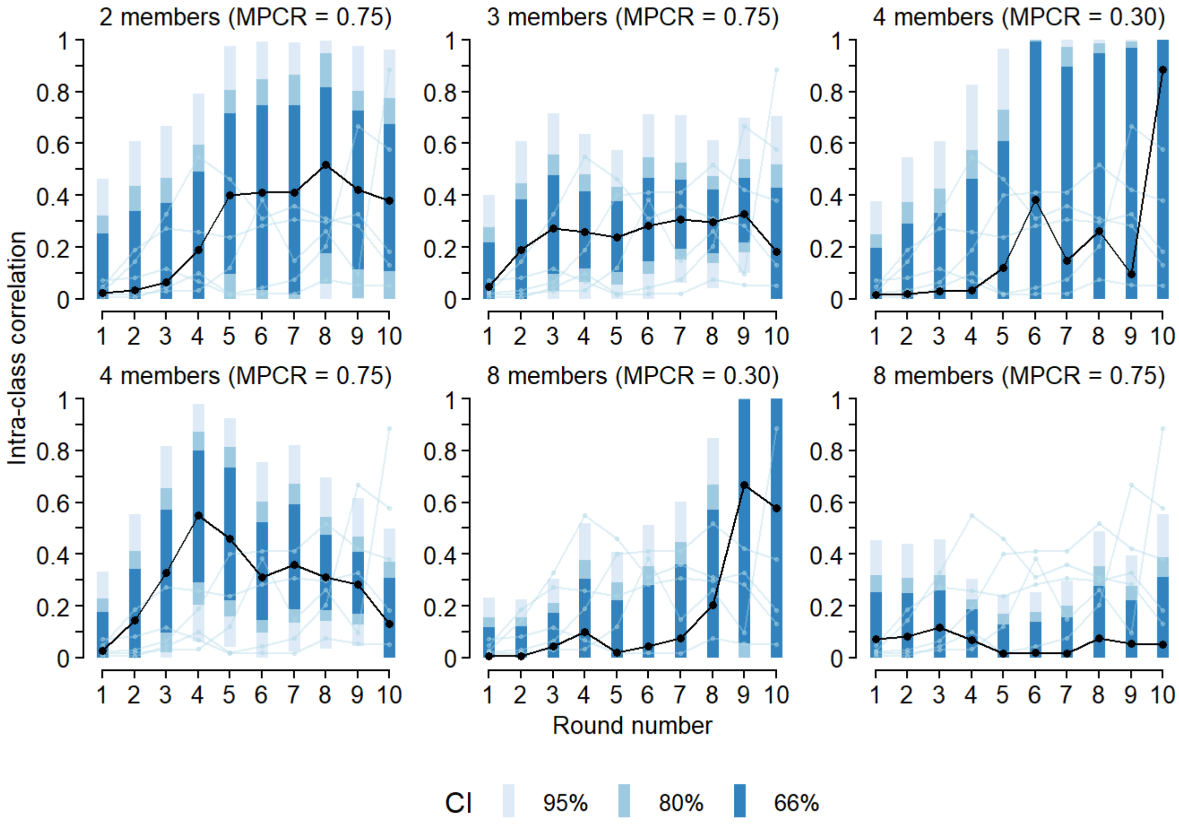

Fig. S36. Intra-class correlation across rounds of play in each experimental condition.

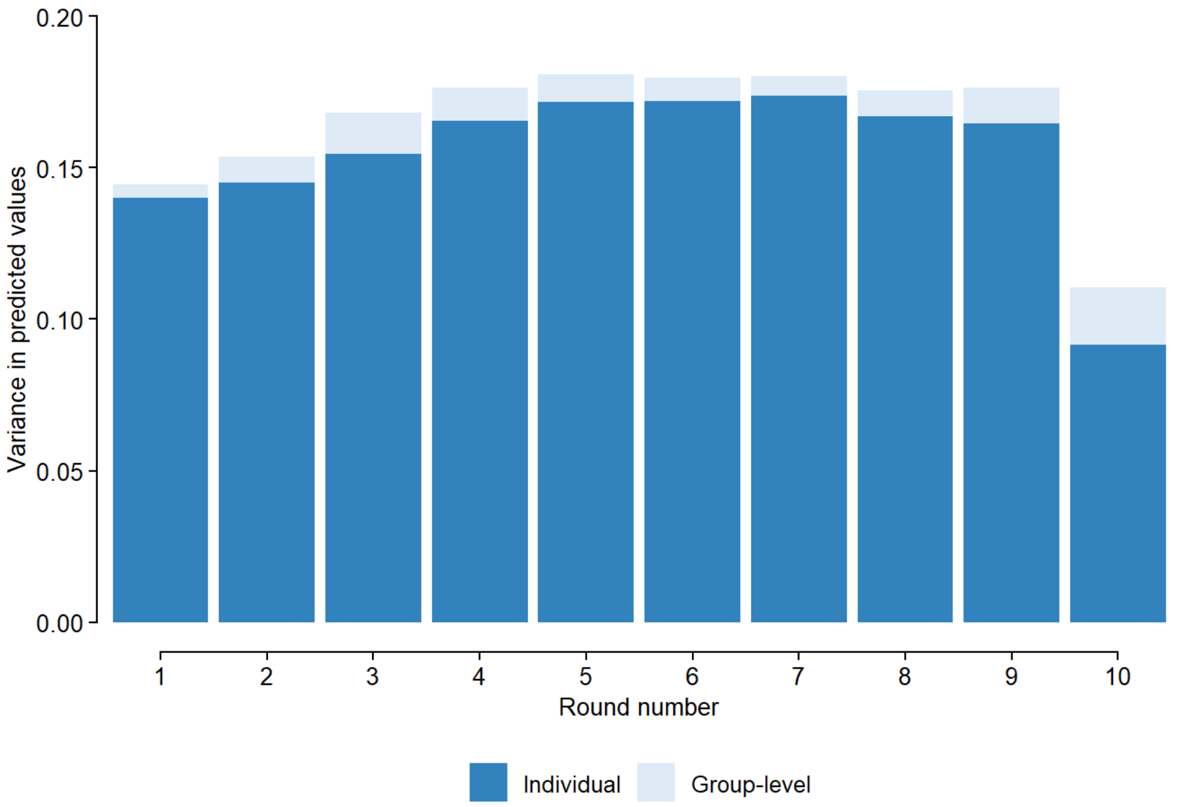

Fig. S37. Variance partitions across rounds of play.

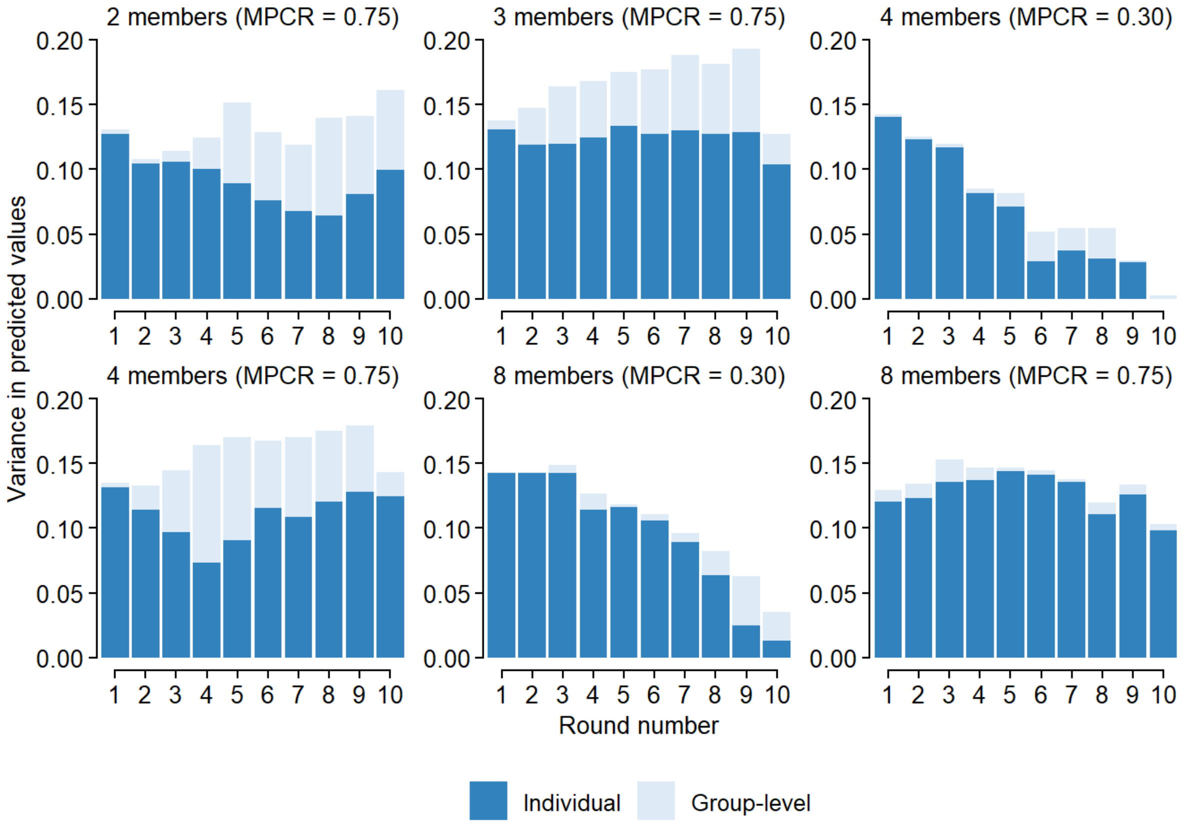

Fig. S38. Variance partitions across rounds of play in each experimental condition.

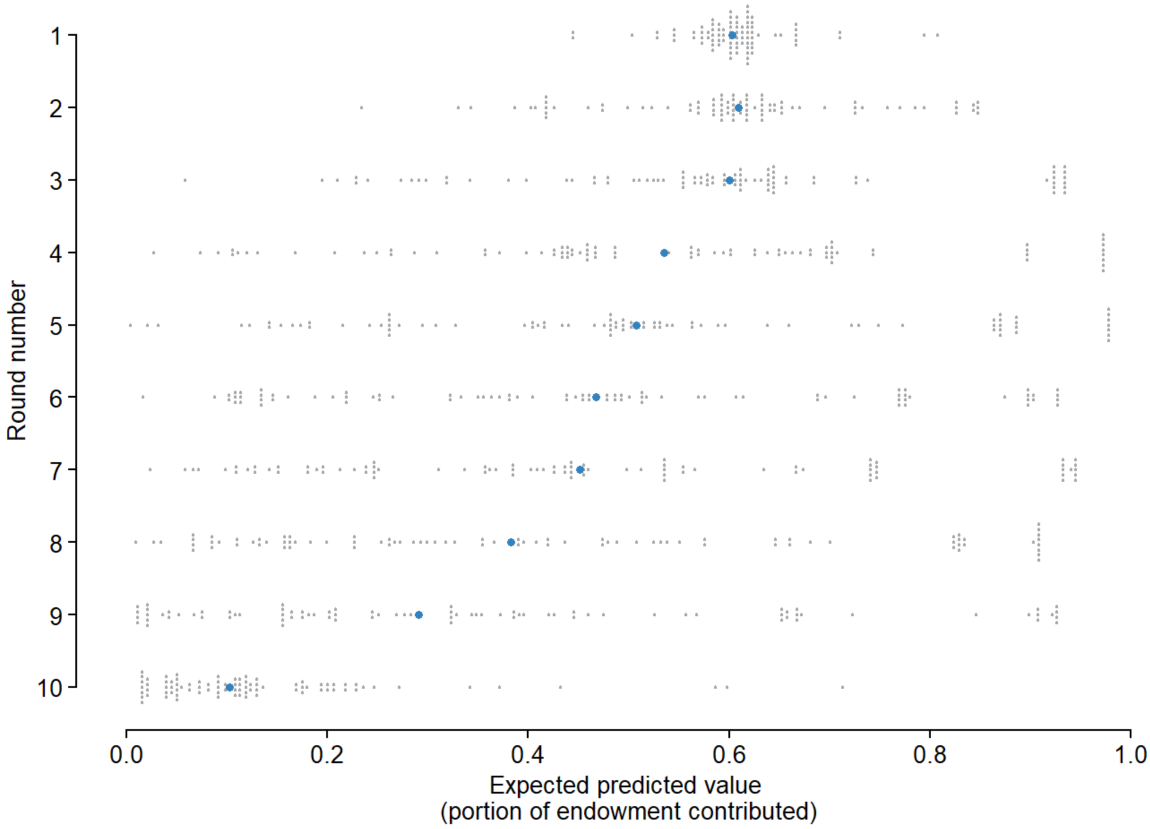

Fig. S39. Overall (blue dot) and group-level (grey dots) predictions across rounds of play.

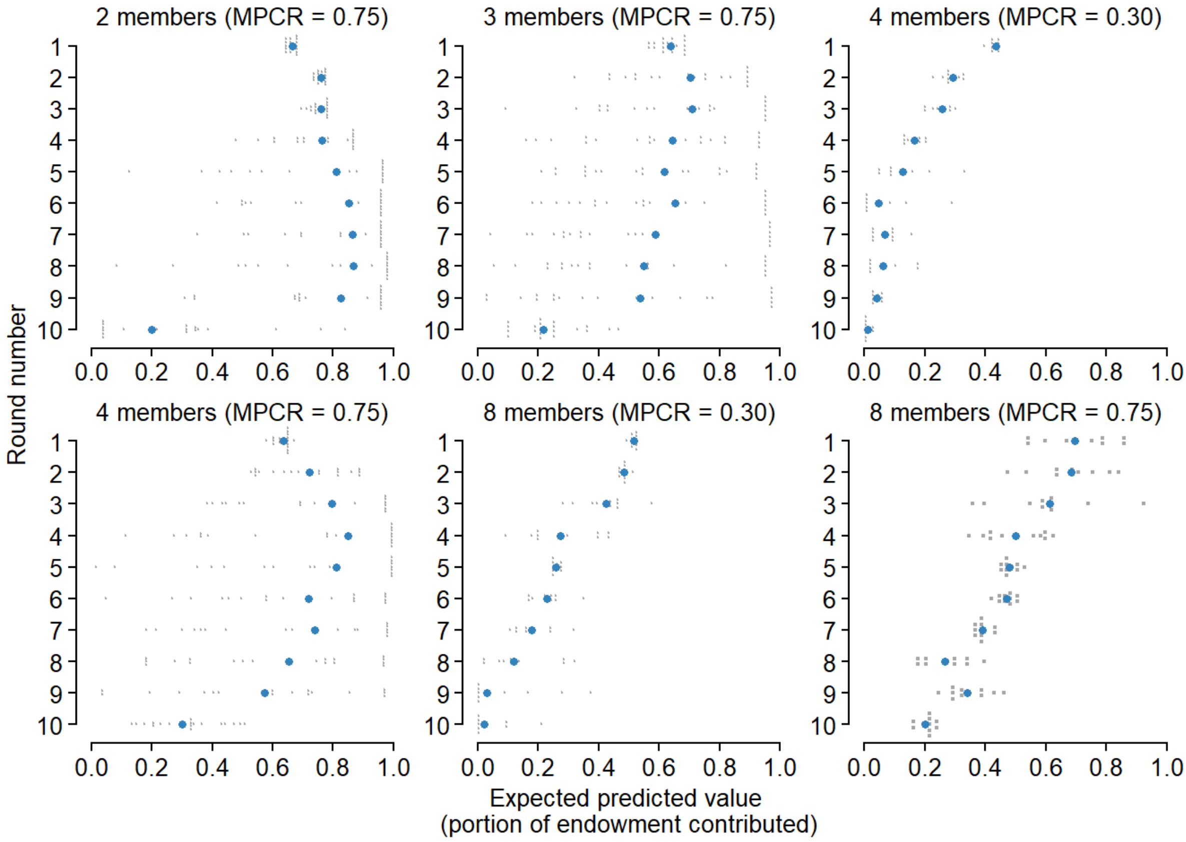

Fig. S40. Overall (blue dot) and group-level (grey dots) predictions across rounds of play in each experimental condition.

Supplementary Figures for Stagnaro et al. (2017)

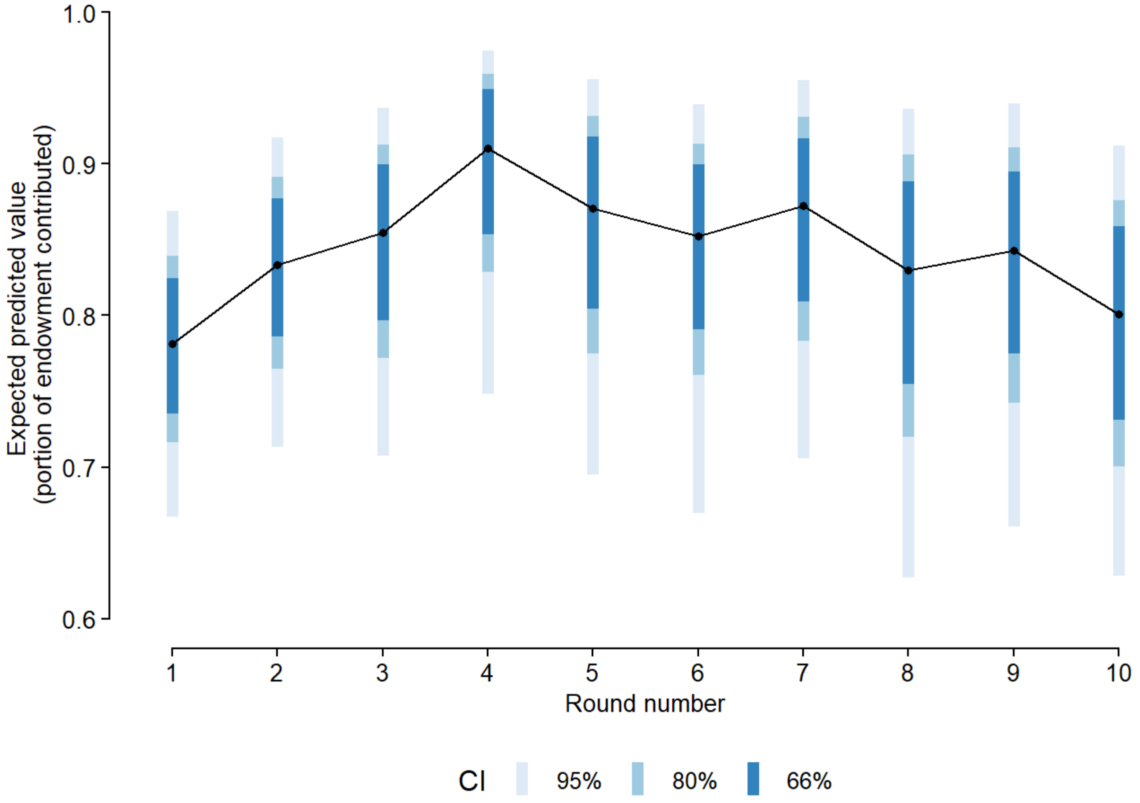

Fig. S41. Predicted level of cooperation across rounds of play.

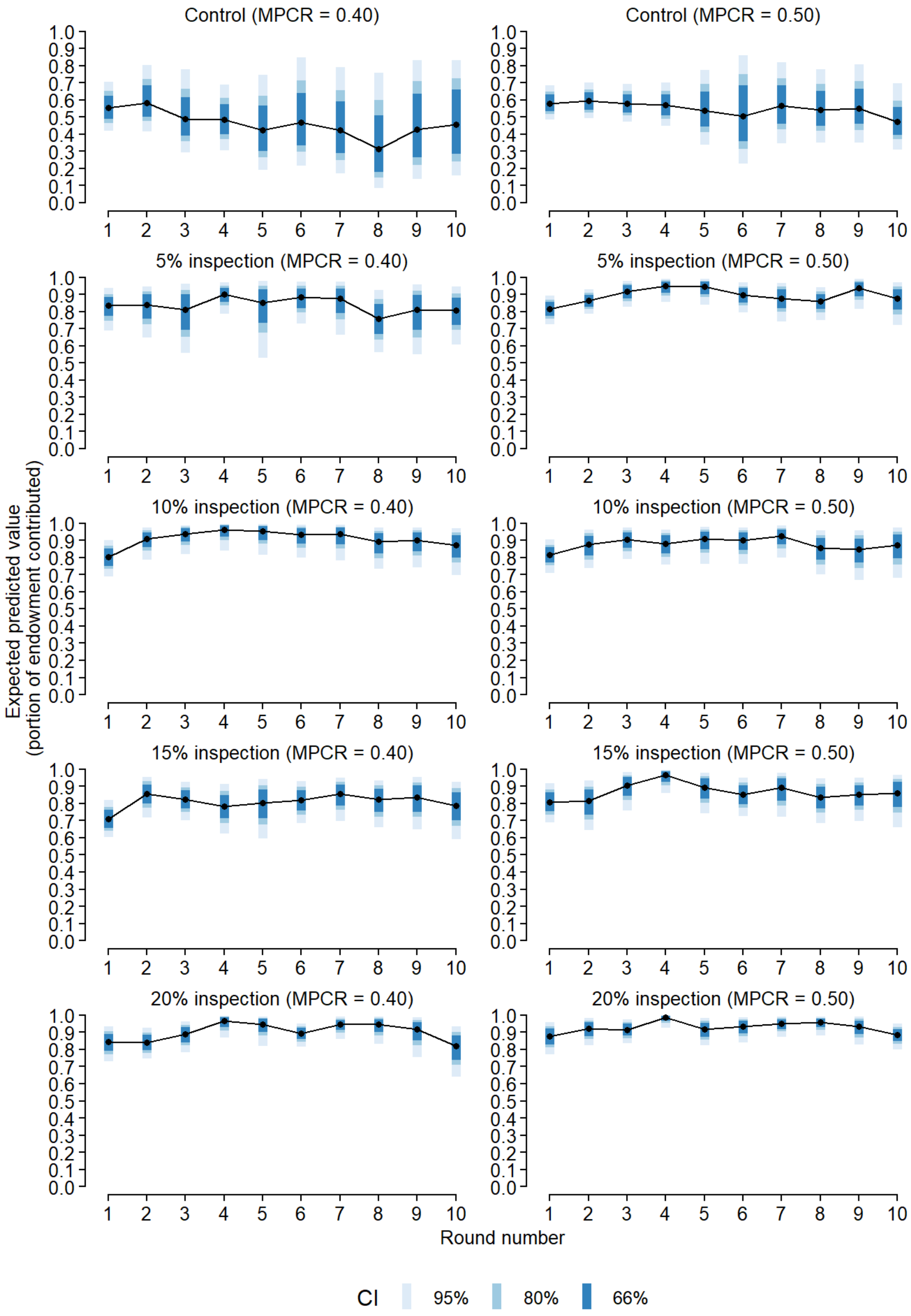

Fig. S42. Predicted level of cooperation across rounds of play in each experimental condition.

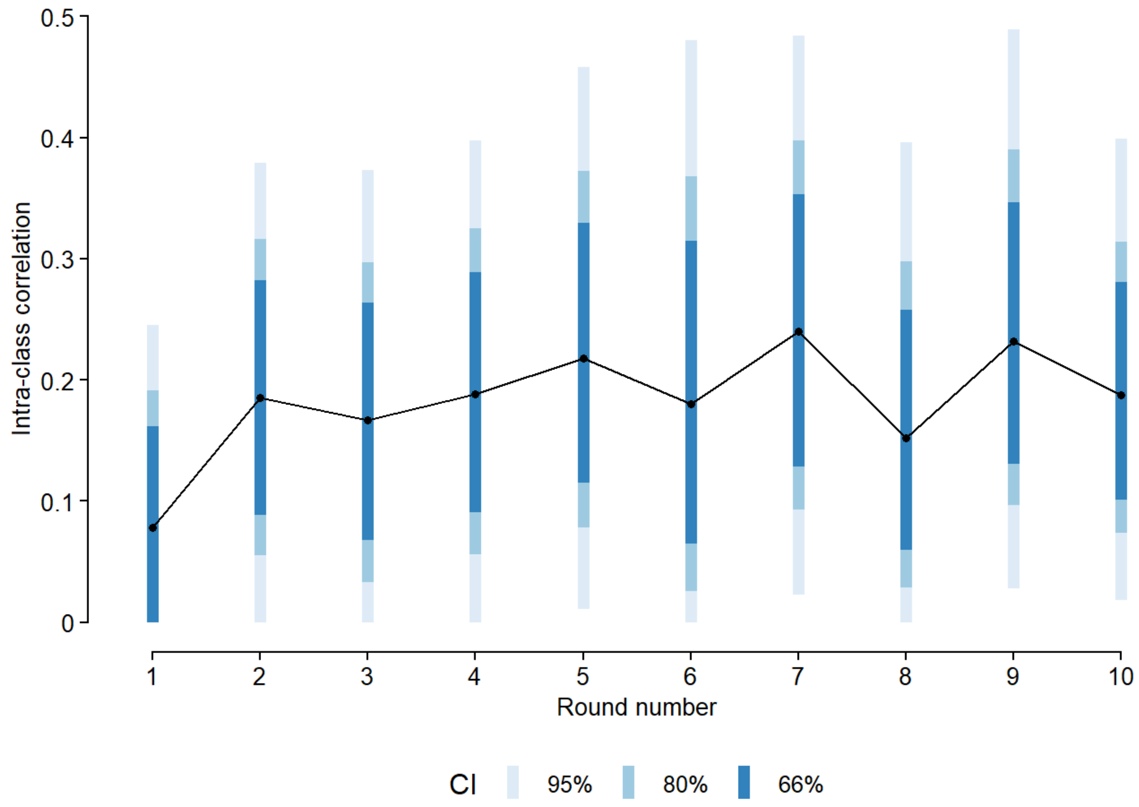

Fig. S43. Intra-class correlation across rounds of play.

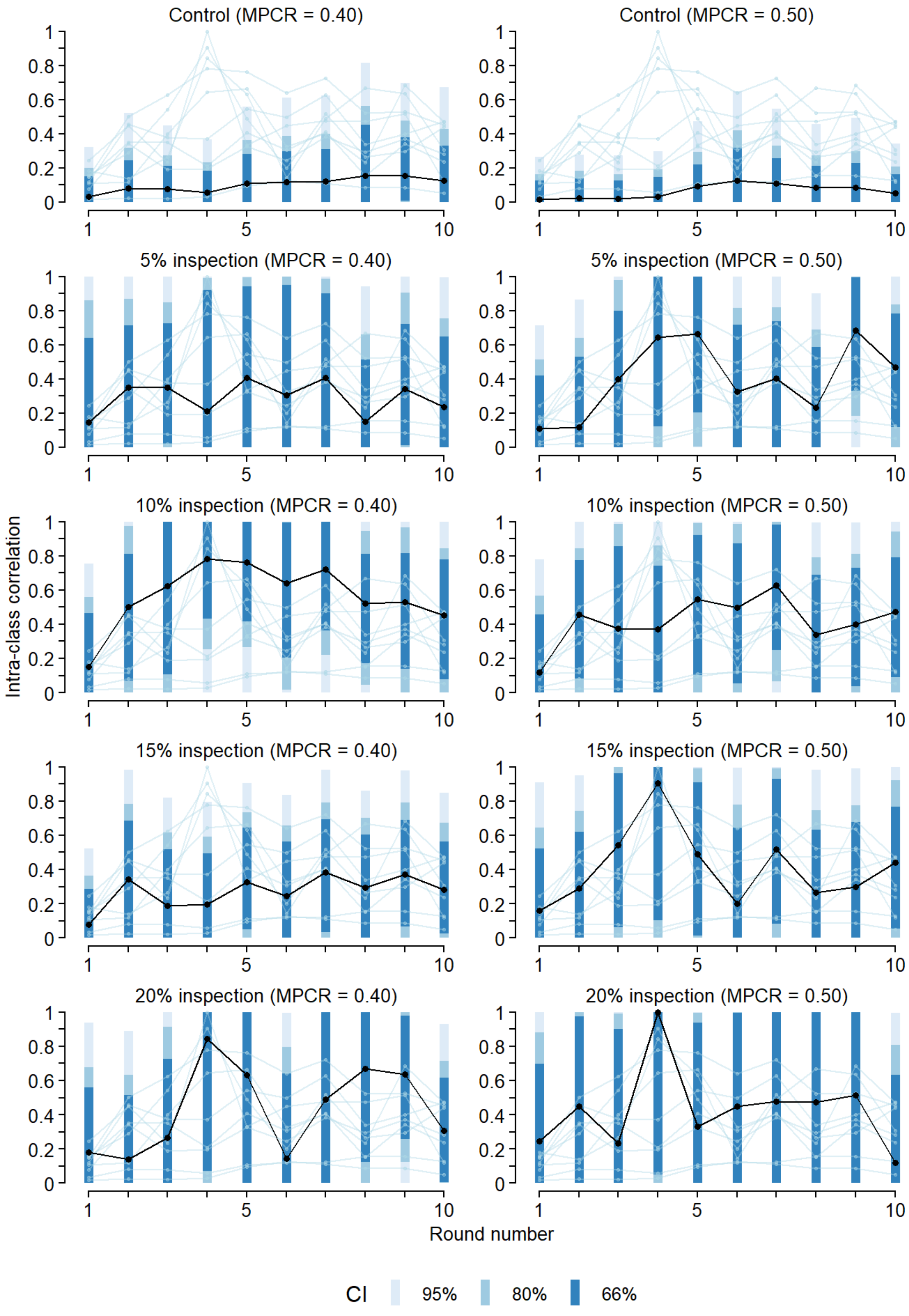

Fig. S44. Intra-class correlation across rounds of play in each experimental condition.

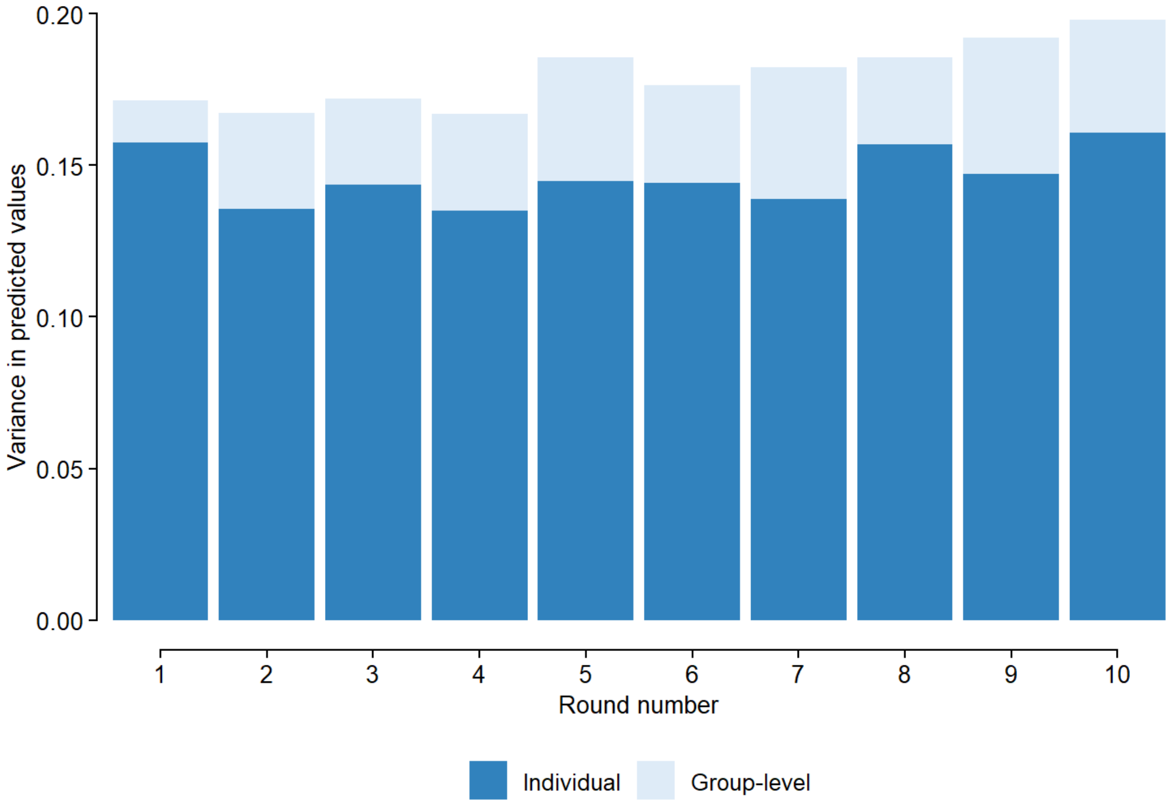

Fig. S45. Variance partitions across rounds of play.

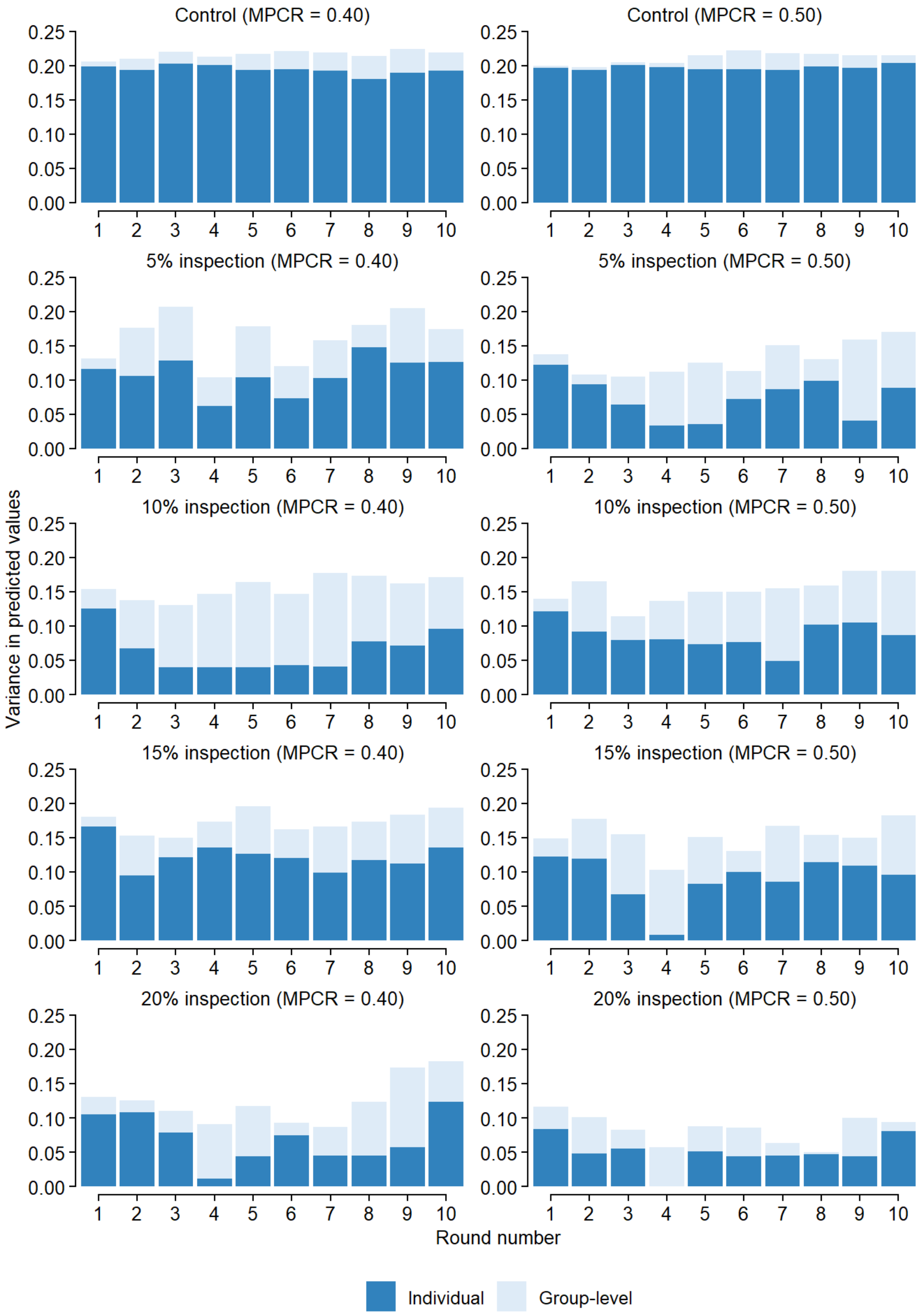

Fig. S46. Variance partitions across rounds of play in each experimental condition.

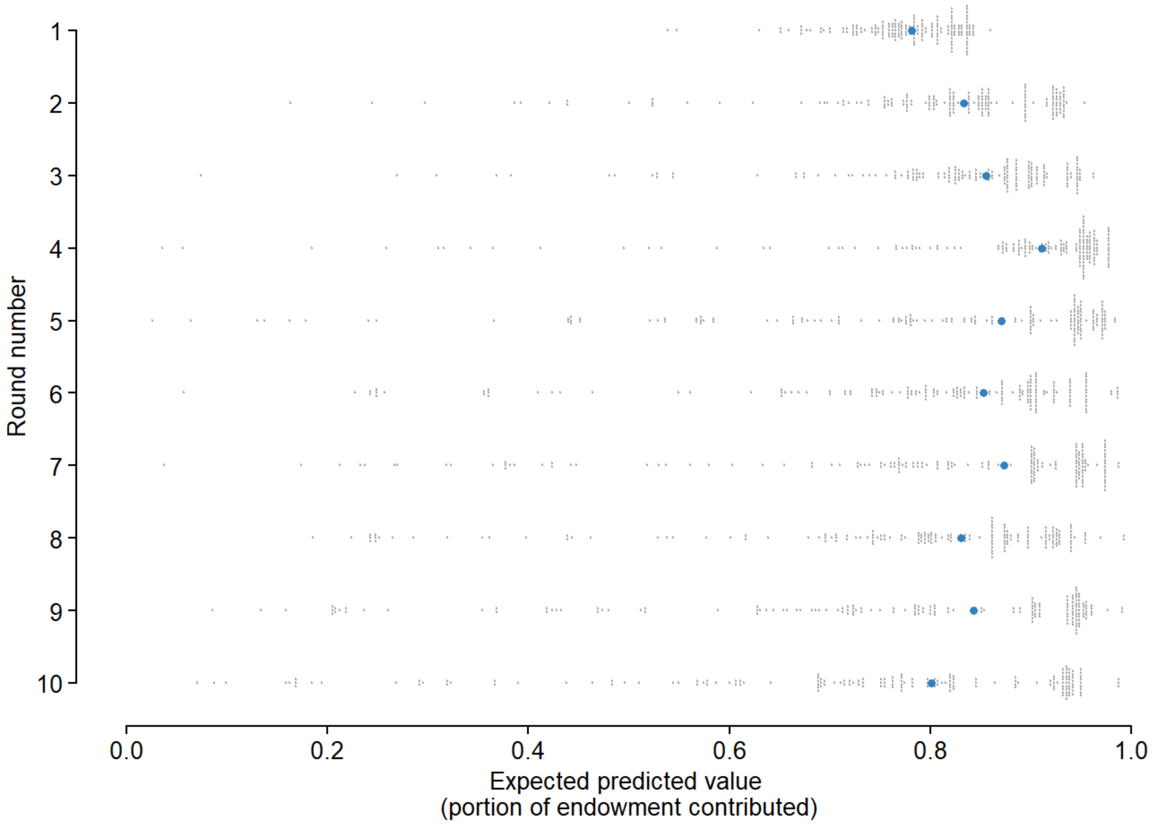

Fig. S47. Overall (blue dot) and group-level (grey dots) predictions across rounds of play.

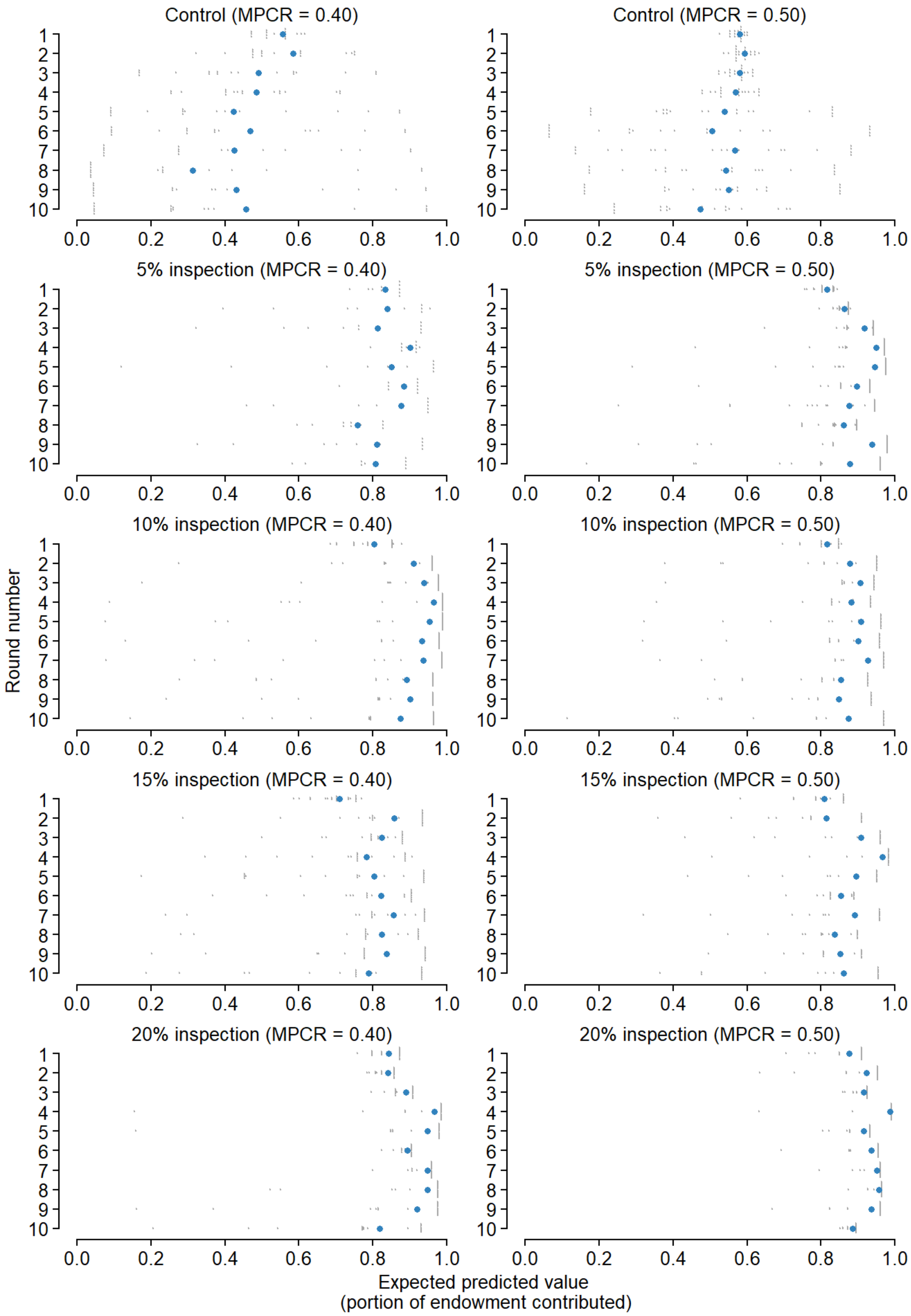

Fig. S48. Overall (blue dot) and group-level (grey dots) predictions across rounds of play in each experimental condition.

Supplementary Figures for Rand et al. (2009)

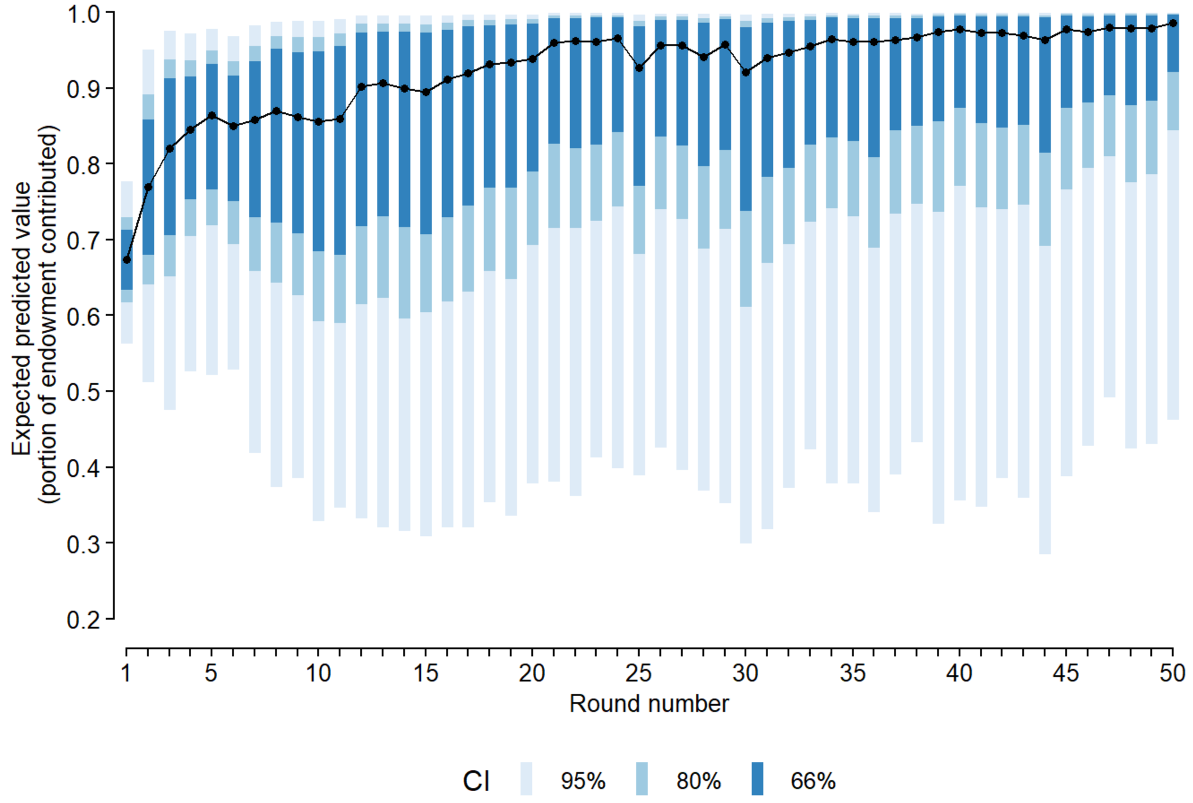

Fig. S49. Predicted level of cooperation across rounds of play.

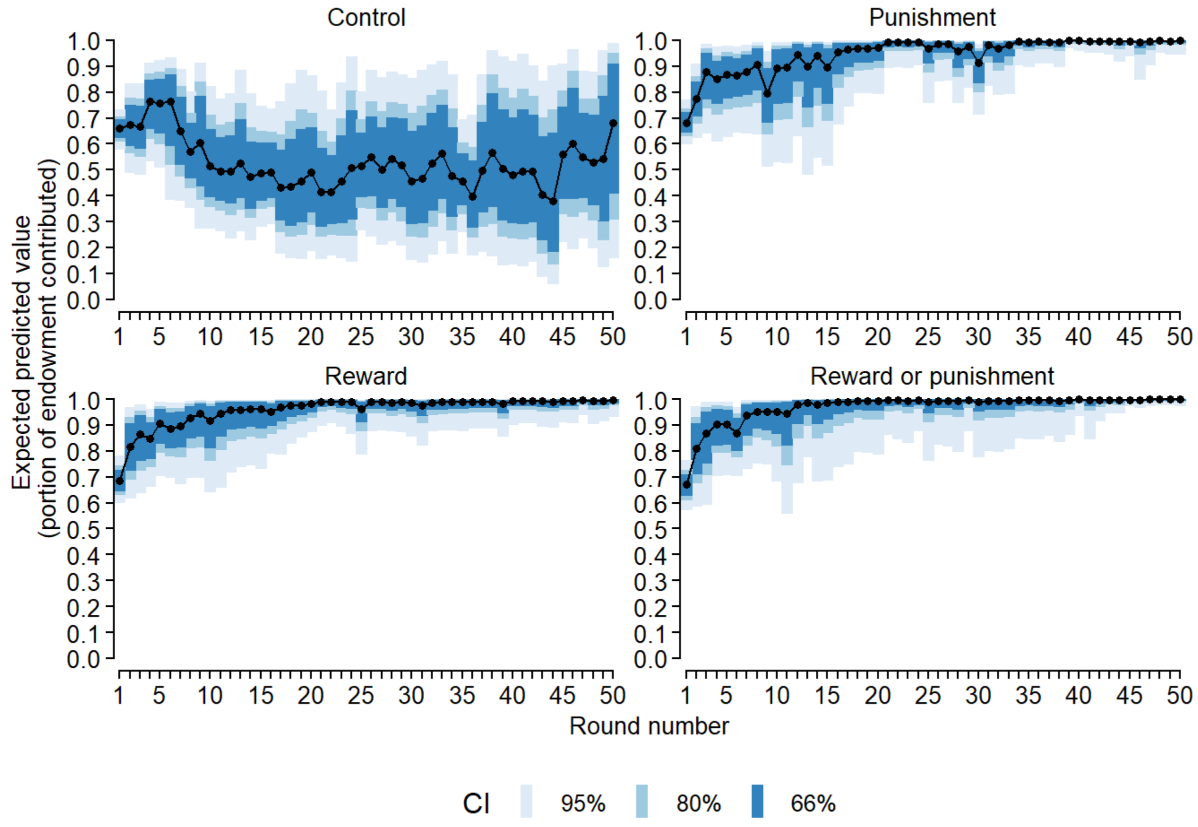

Fig. S50. Predicted level of cooperation across rounds of play in each experimental condition.

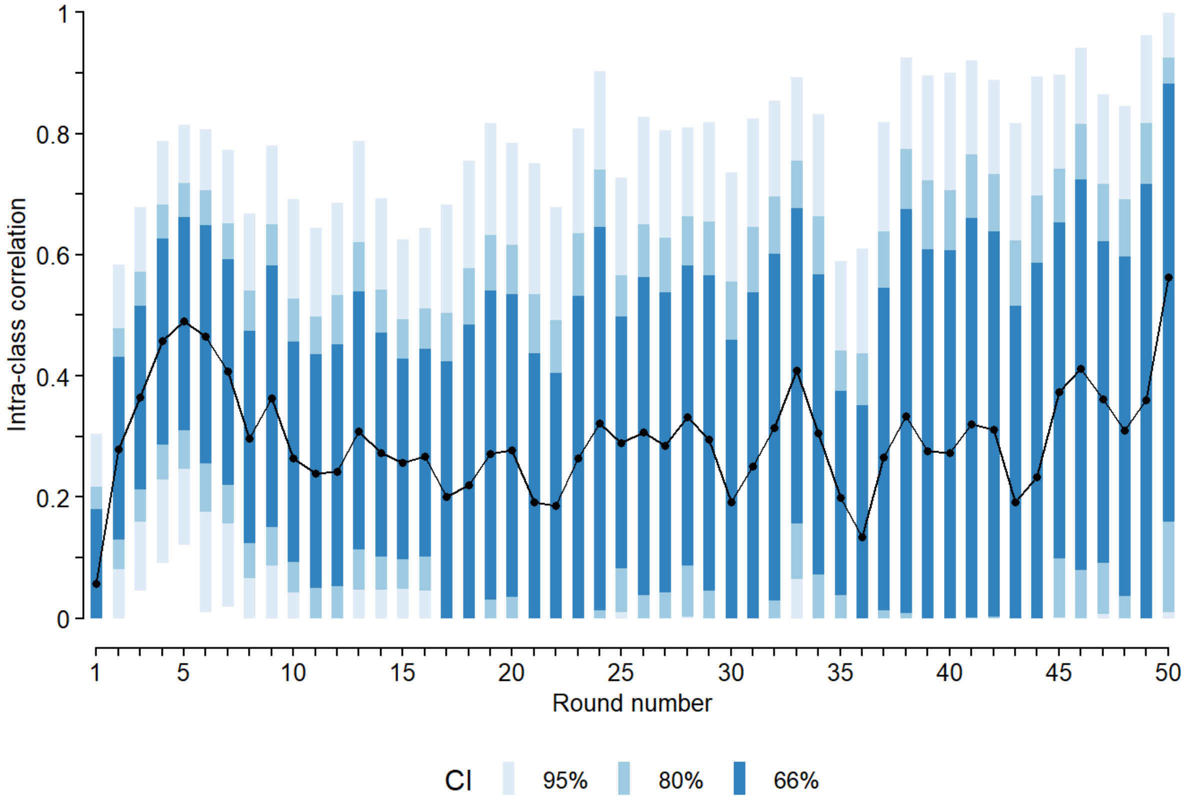

Fig. S51. Intra-class correlation across rounds of play.

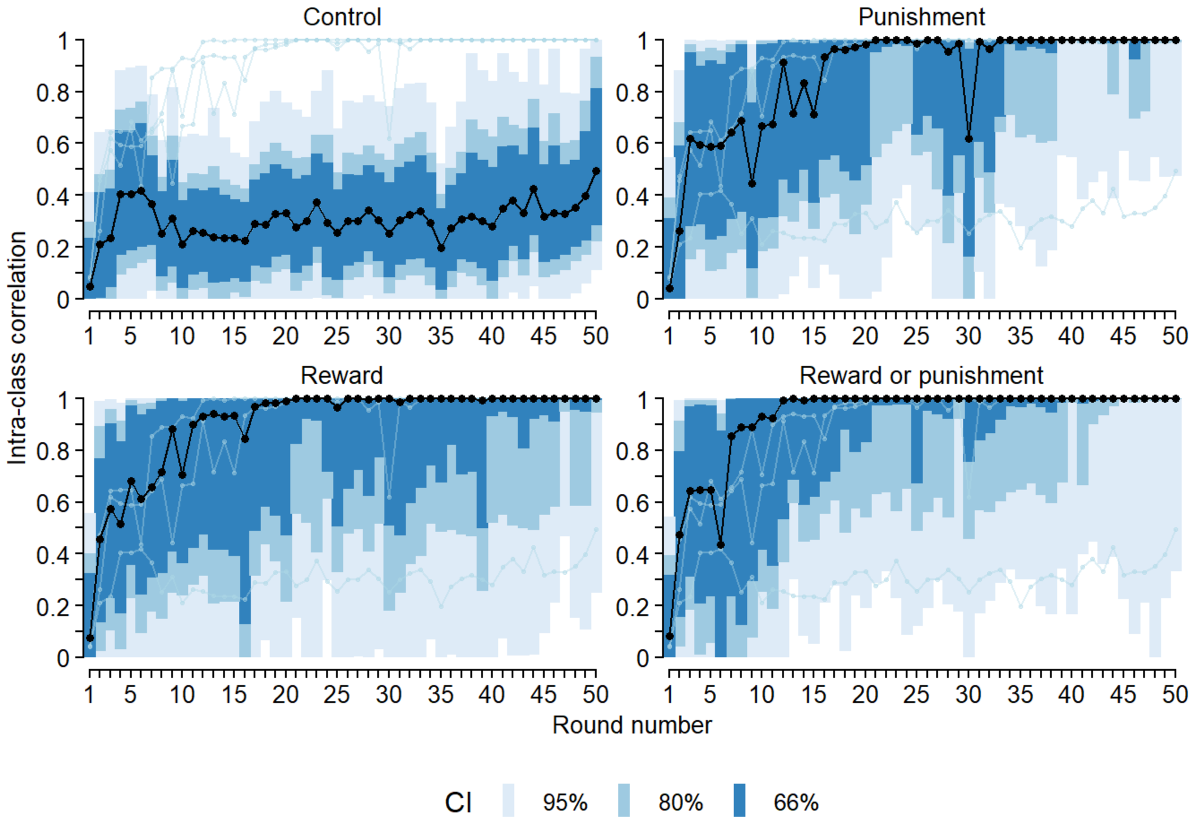

Fig. S52. Intra-class correlation across rounds of play in each experimental condition.

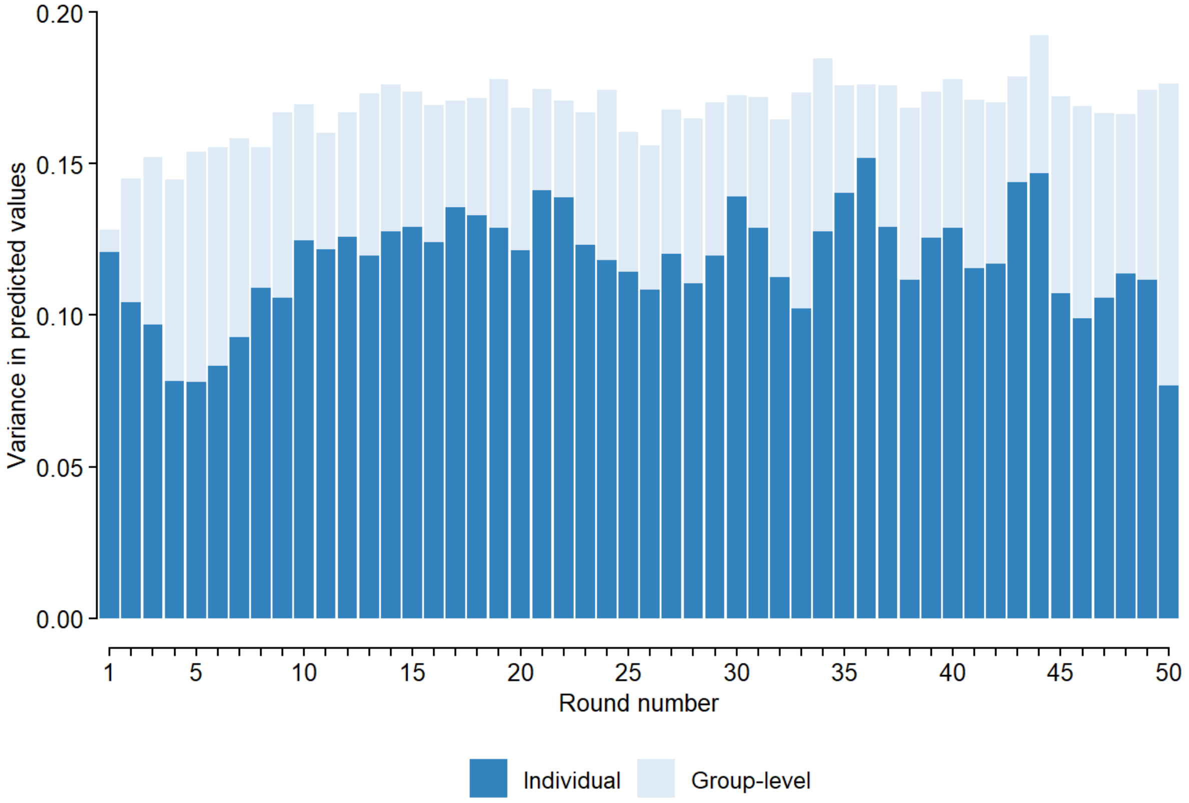

Fig. S53. Variance partitions across rounds of play.

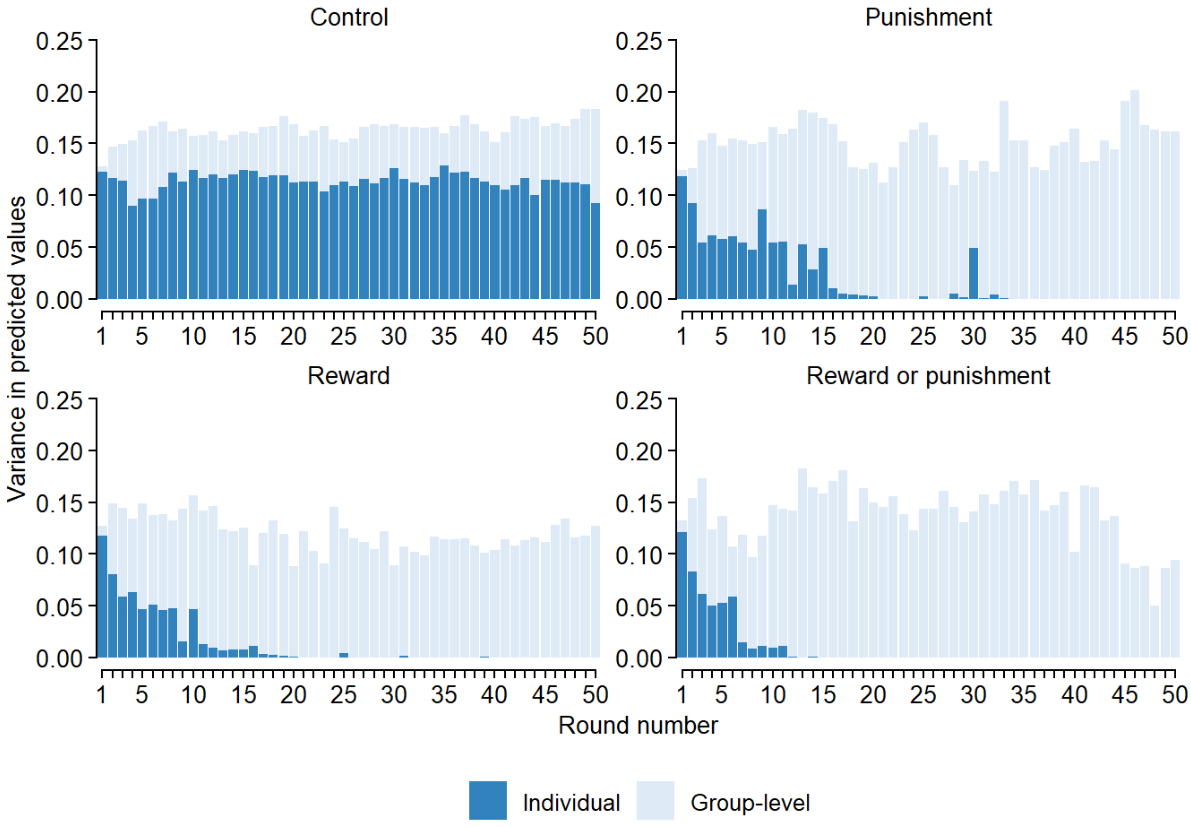

Fig. S54. Variance partitions across rounds of play in each experimental condition.

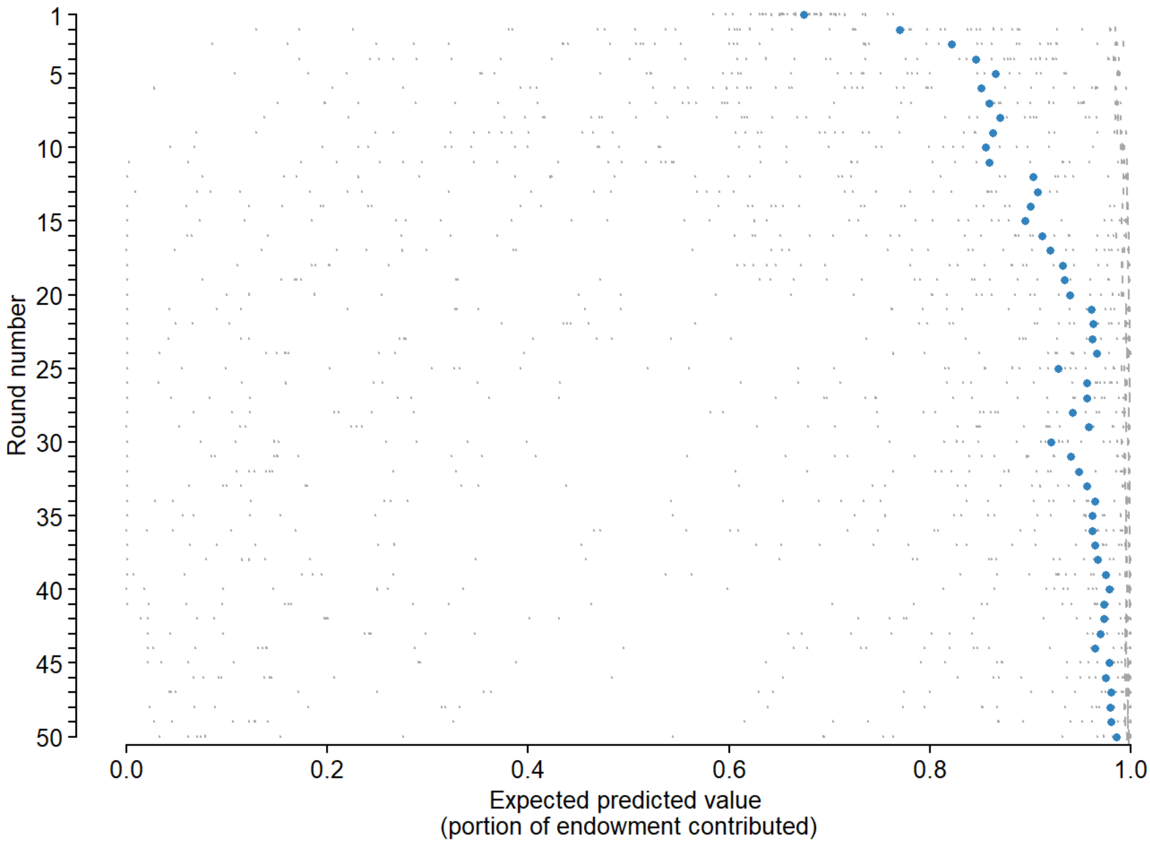

Fig. S55. Overall (blue dot) and group-level (grey dots) predictions across rounds of play.

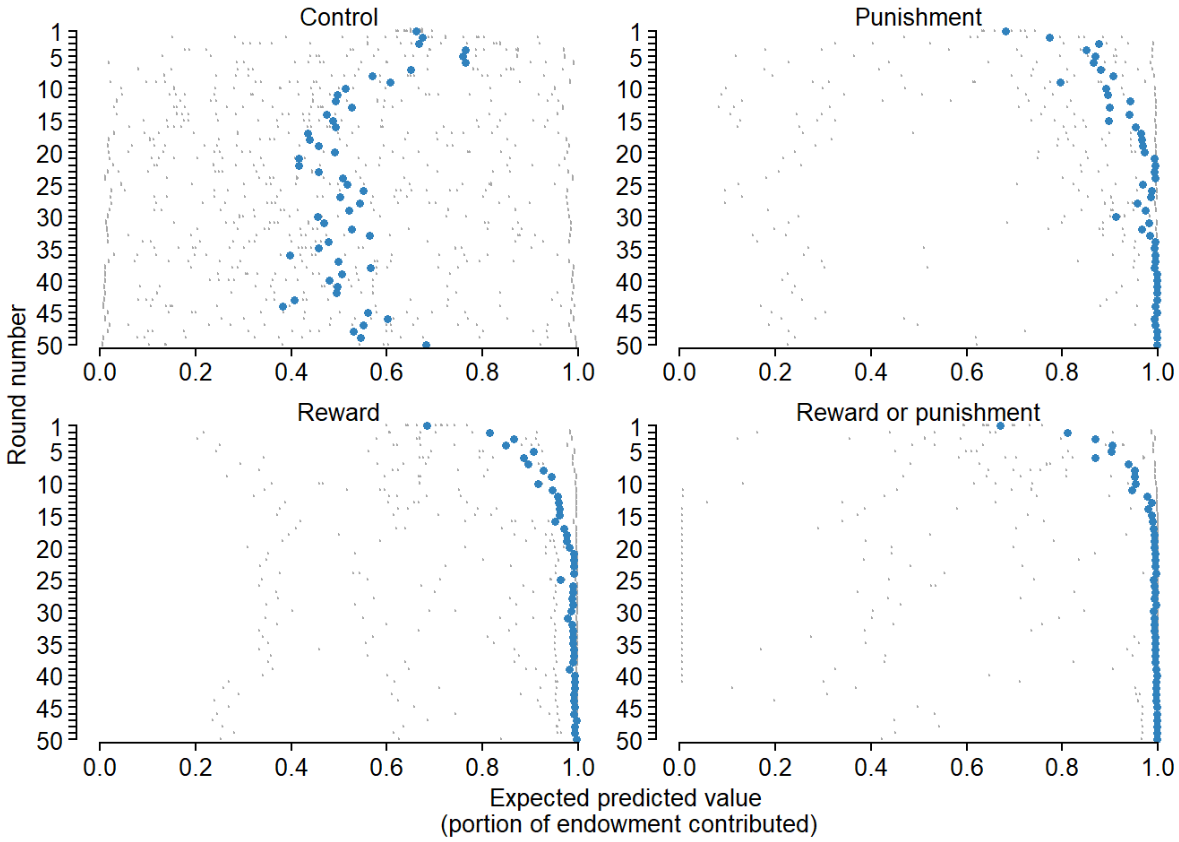

Fig. S56. Overall (blue dot) and group-level (grey dots) predictions across rounds of play in each experimental condition.

Supplementary Figures for Gross et al. (2022)

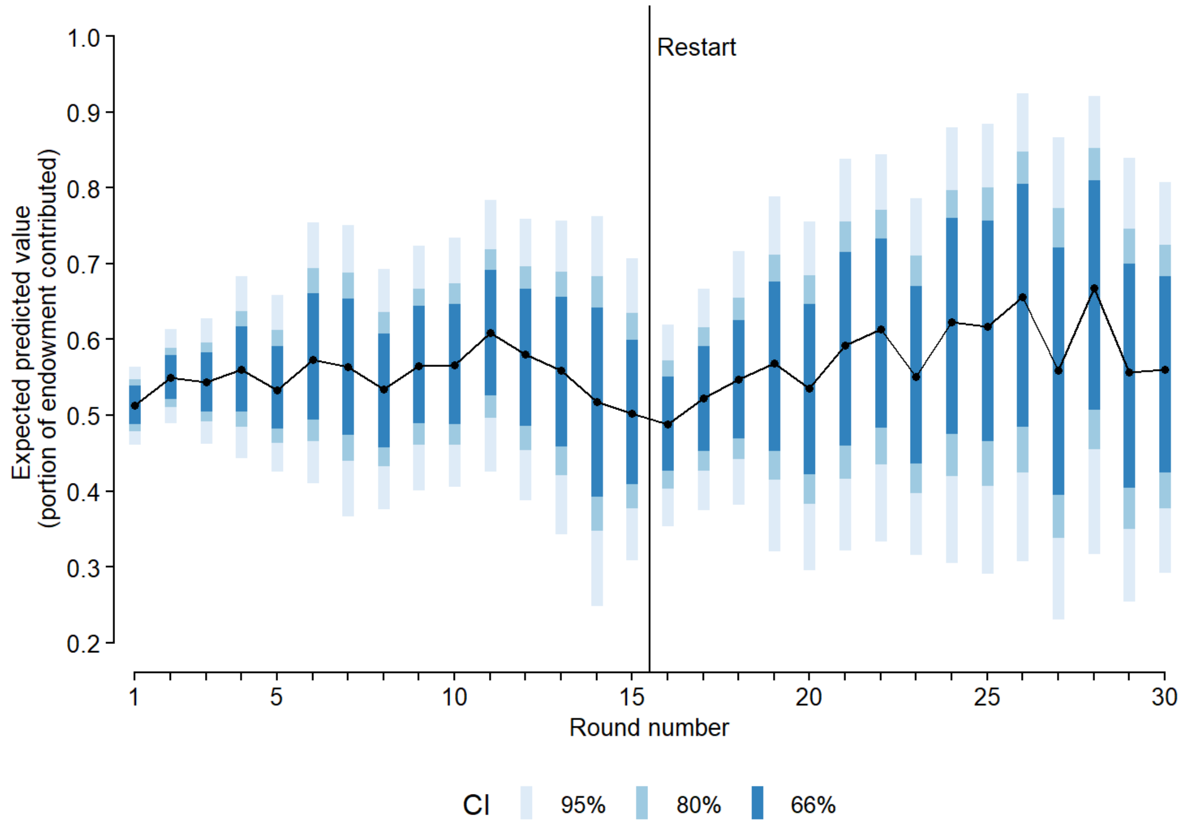

Fig. S57. Predicted level of cooperation across rounds of play.

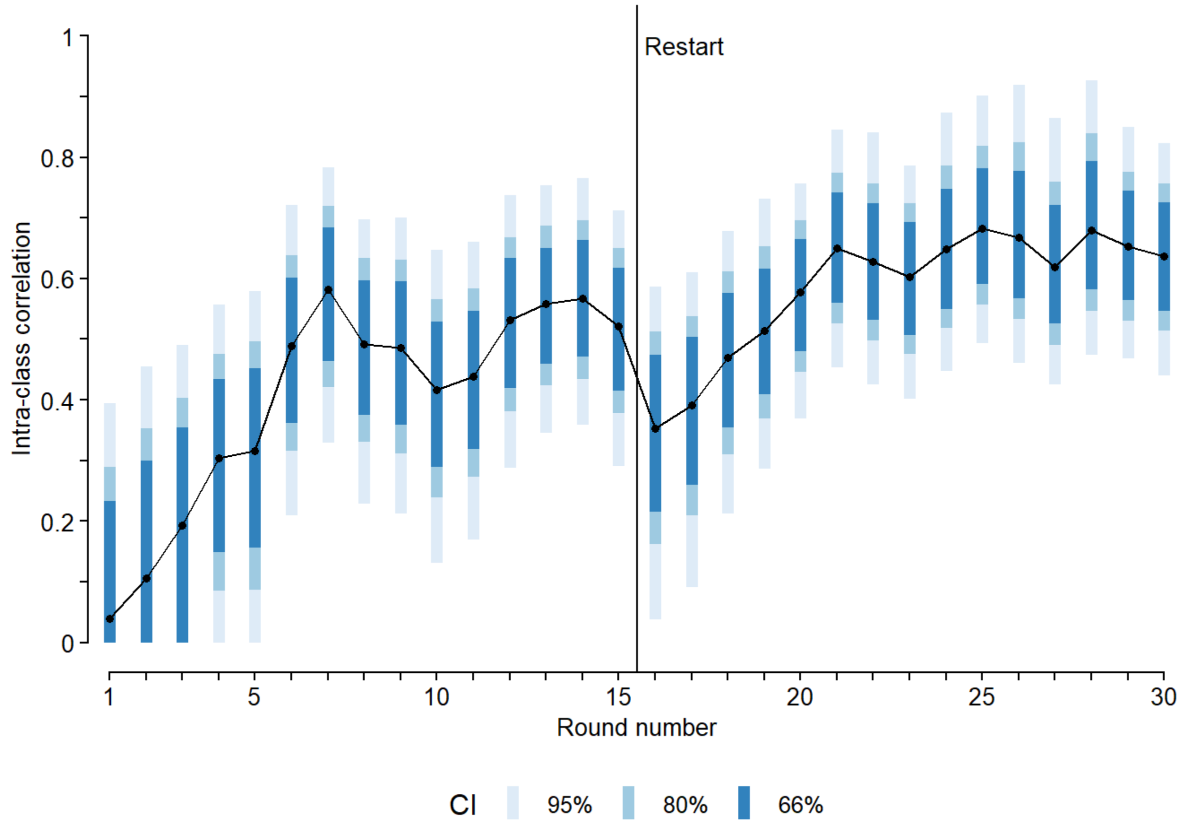

Fig. S58. Intra-class correlation across rounds of play.

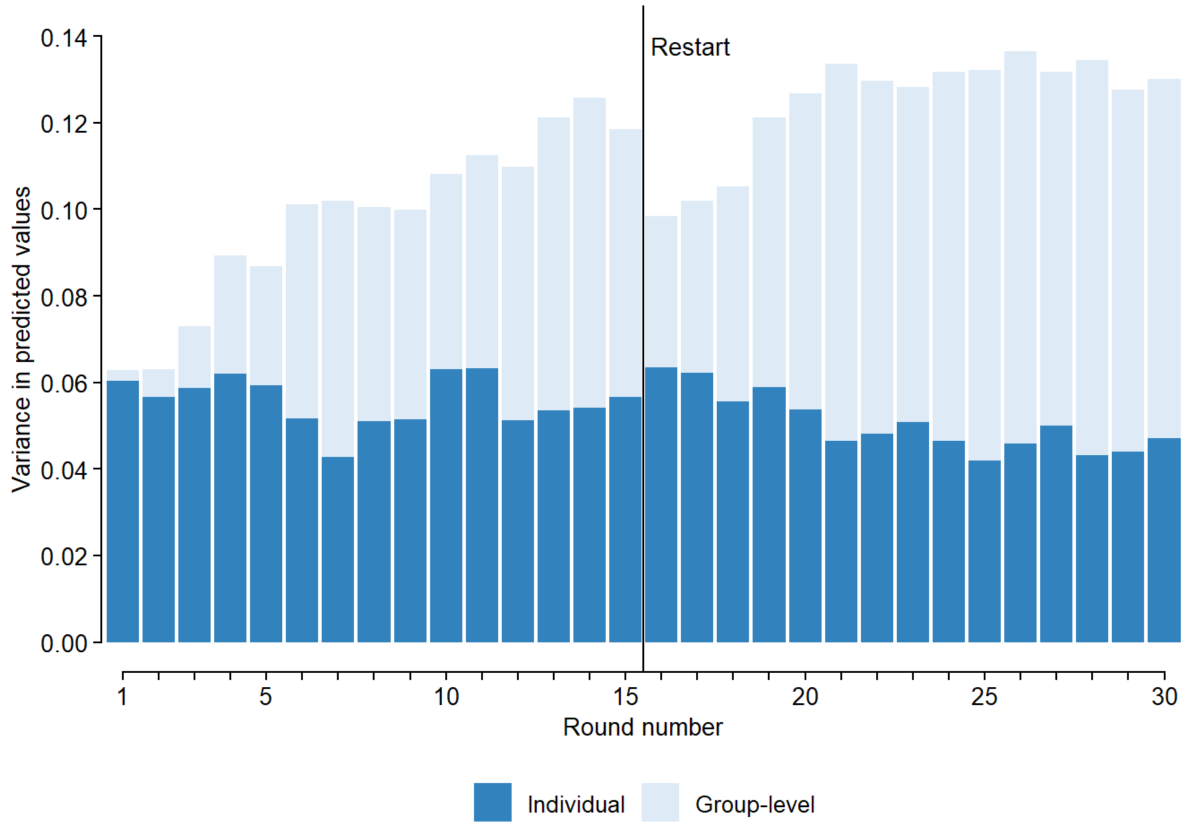

Fig. S59. Variance partitions across rounds of play.

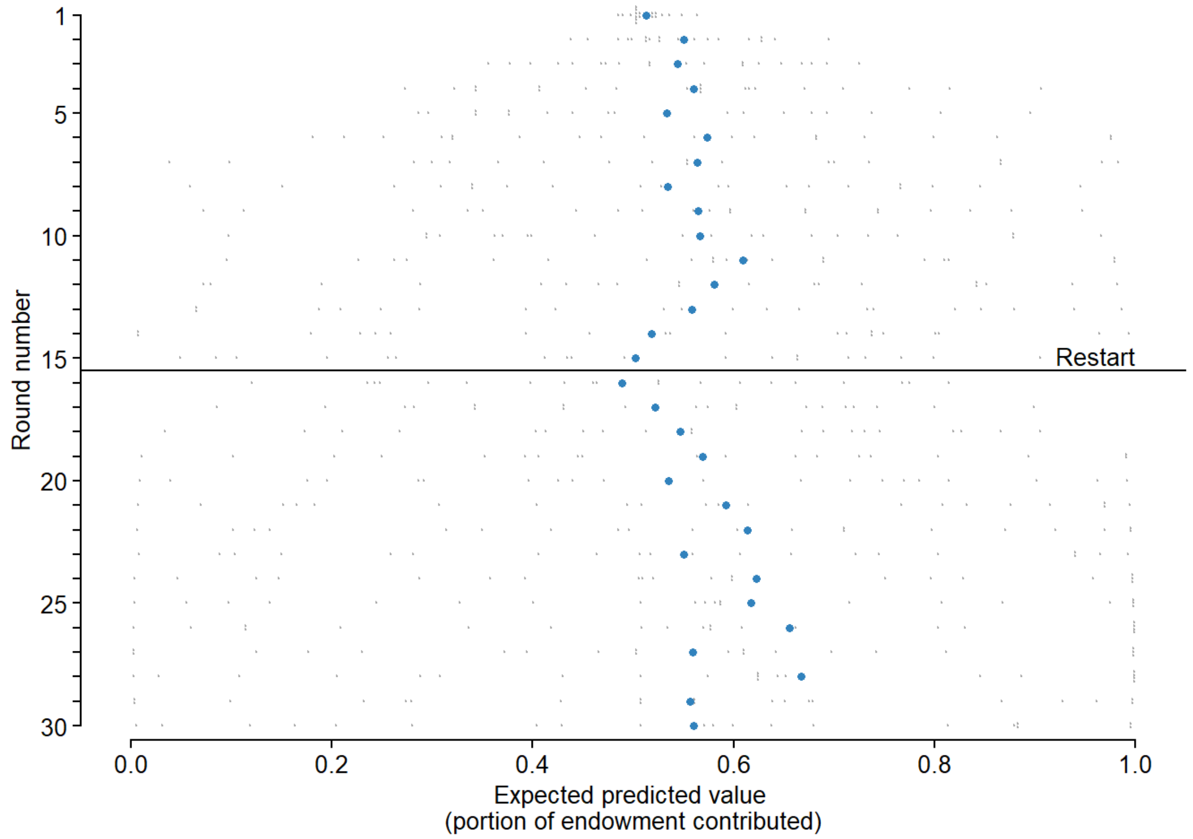

Fig. S60. Overall (blue dot) and group-level (grey dots) predictions across rounds of play.

Supplementary Figures for Herrmann et al. (2008)

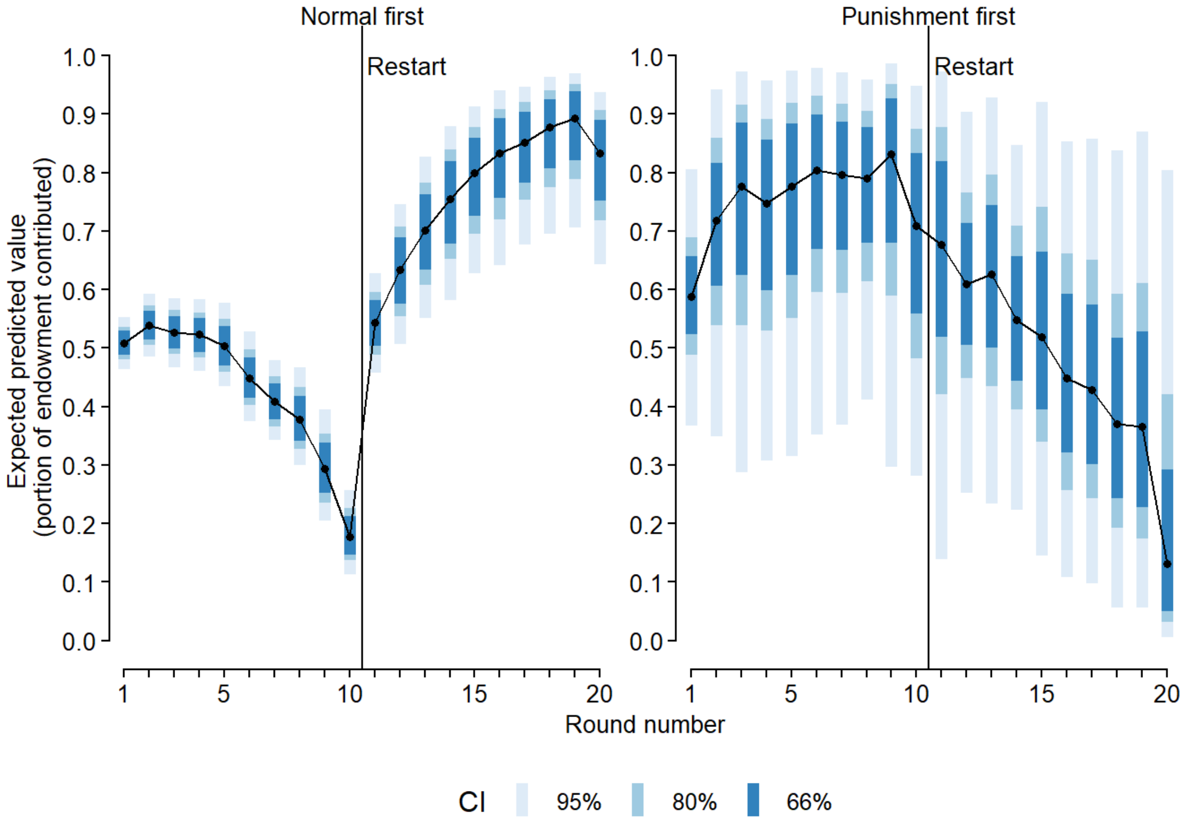

Fig. S61. Predicted level of cooperation across rounds of play in each experimental condition.

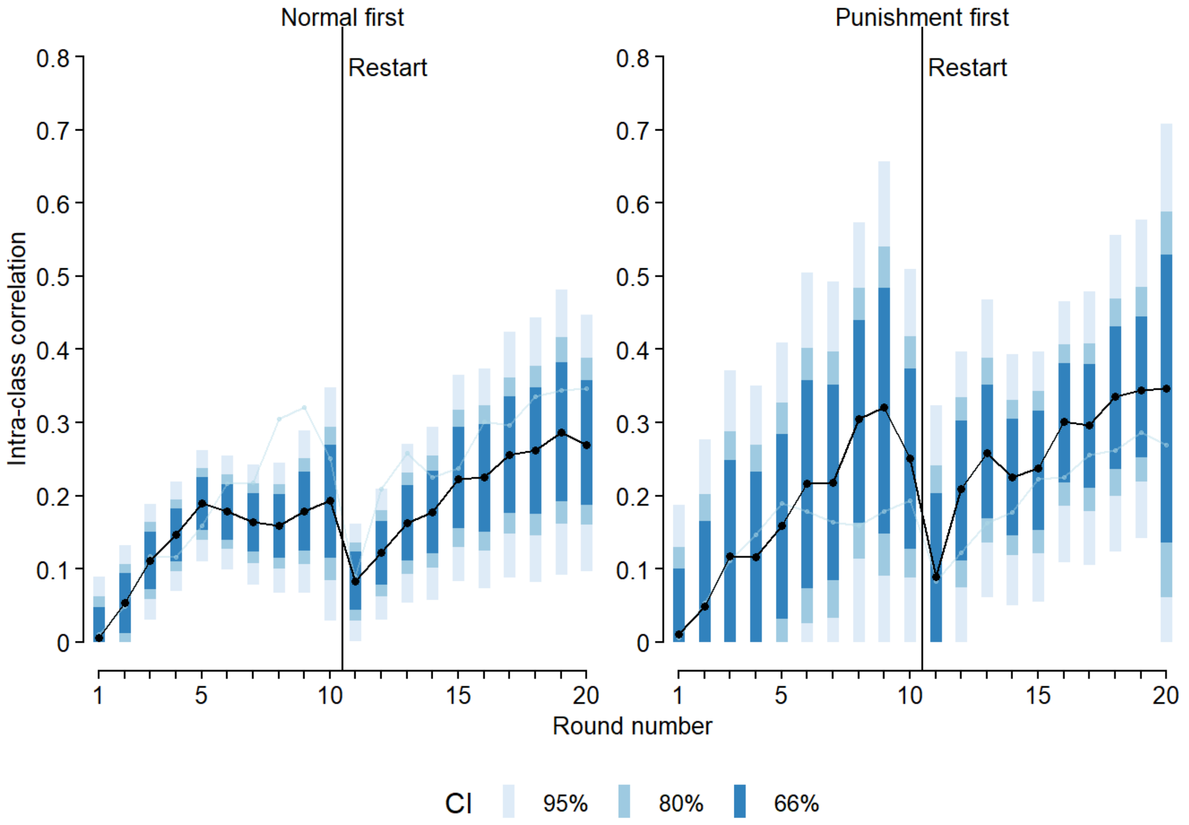

Fig. S62. Intra-class correlation across rounds of play in each experimental condition.

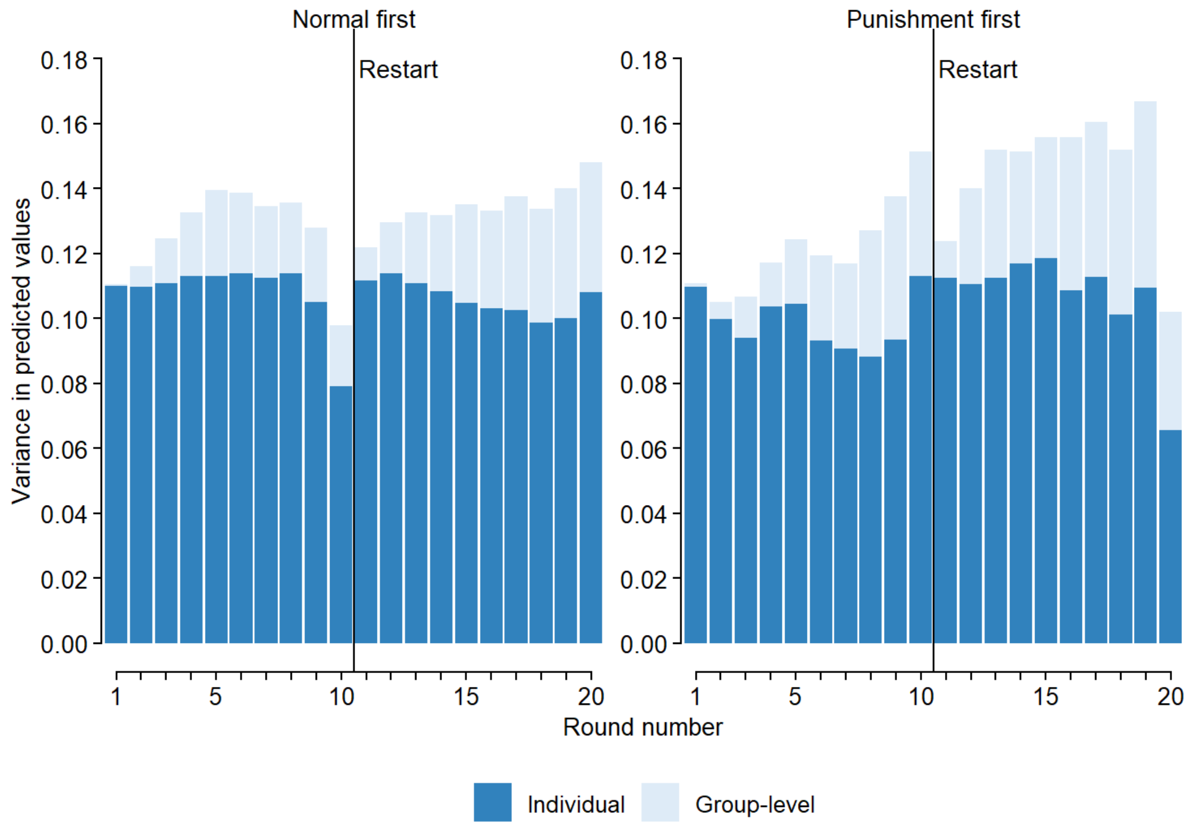

Fig. S63. Variance partitions across rounds of play in each experimental condition.

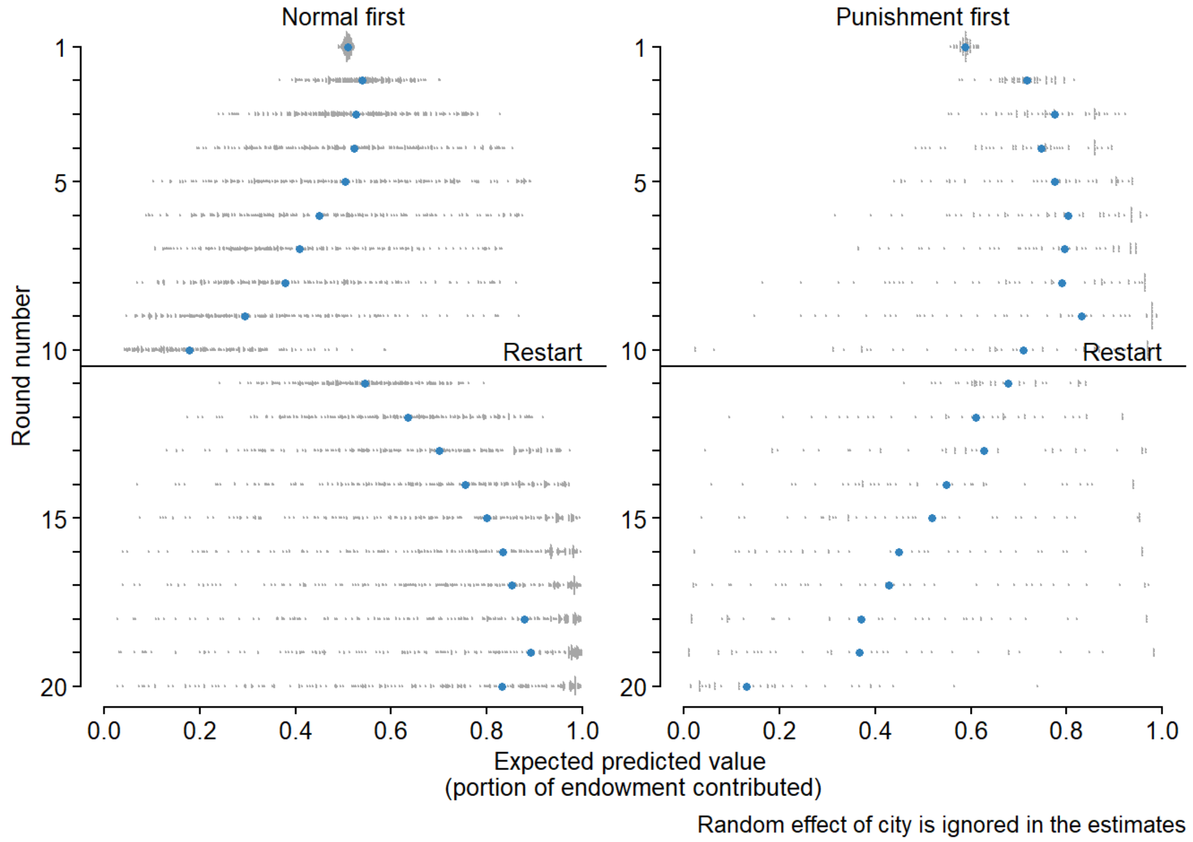

Fig. S64. Overall (blue dot) and group-level (grey dots) predictions across rounds of play in each experimental condition.

Supplementary Figures for Arechar et al. (2018)

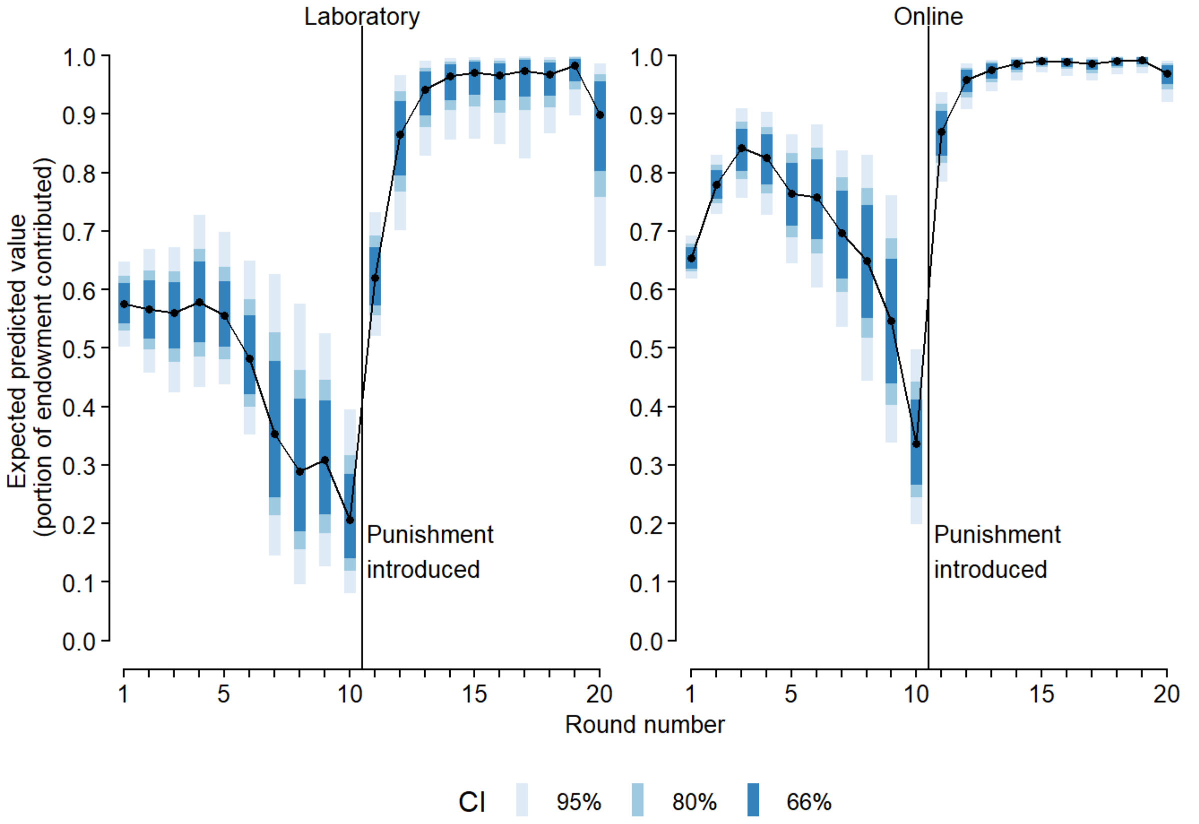

Fig. S65. Predicted level of cooperation across rounds of play in each experimental condition.

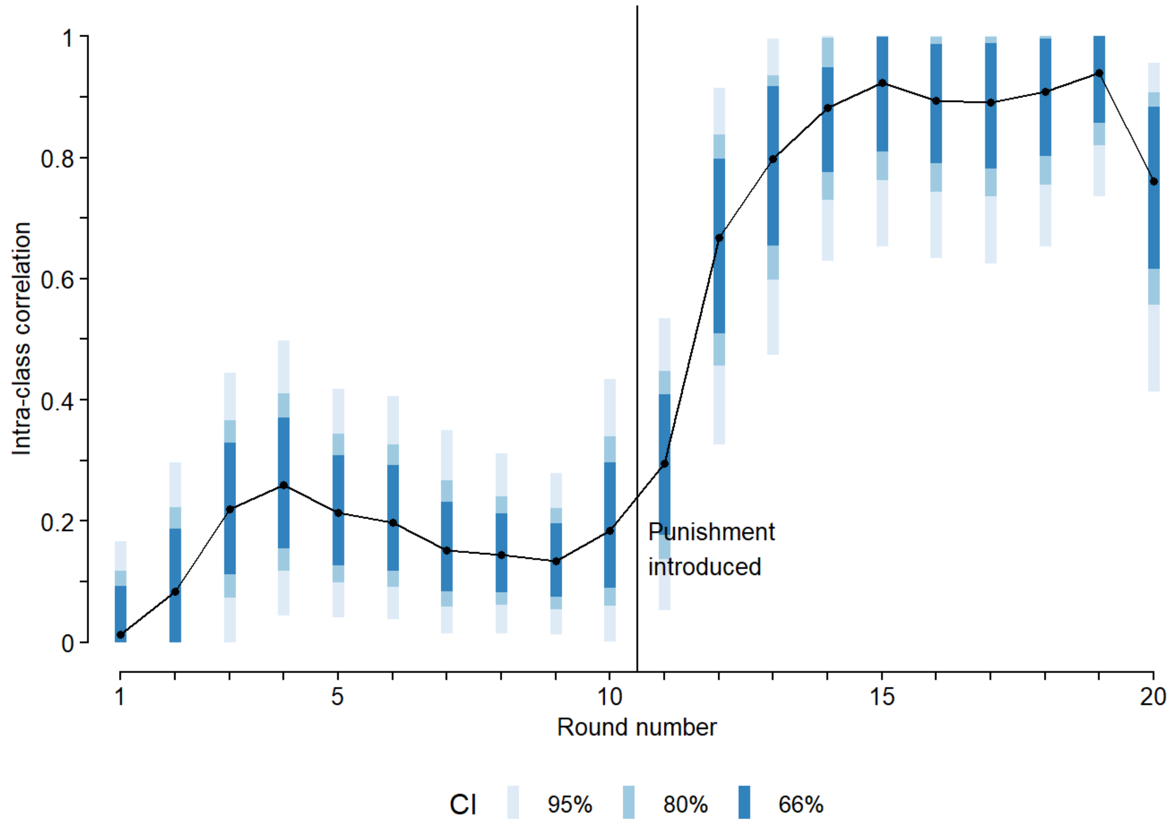

Fig. S66. Intra-class correlation across rounds of play.

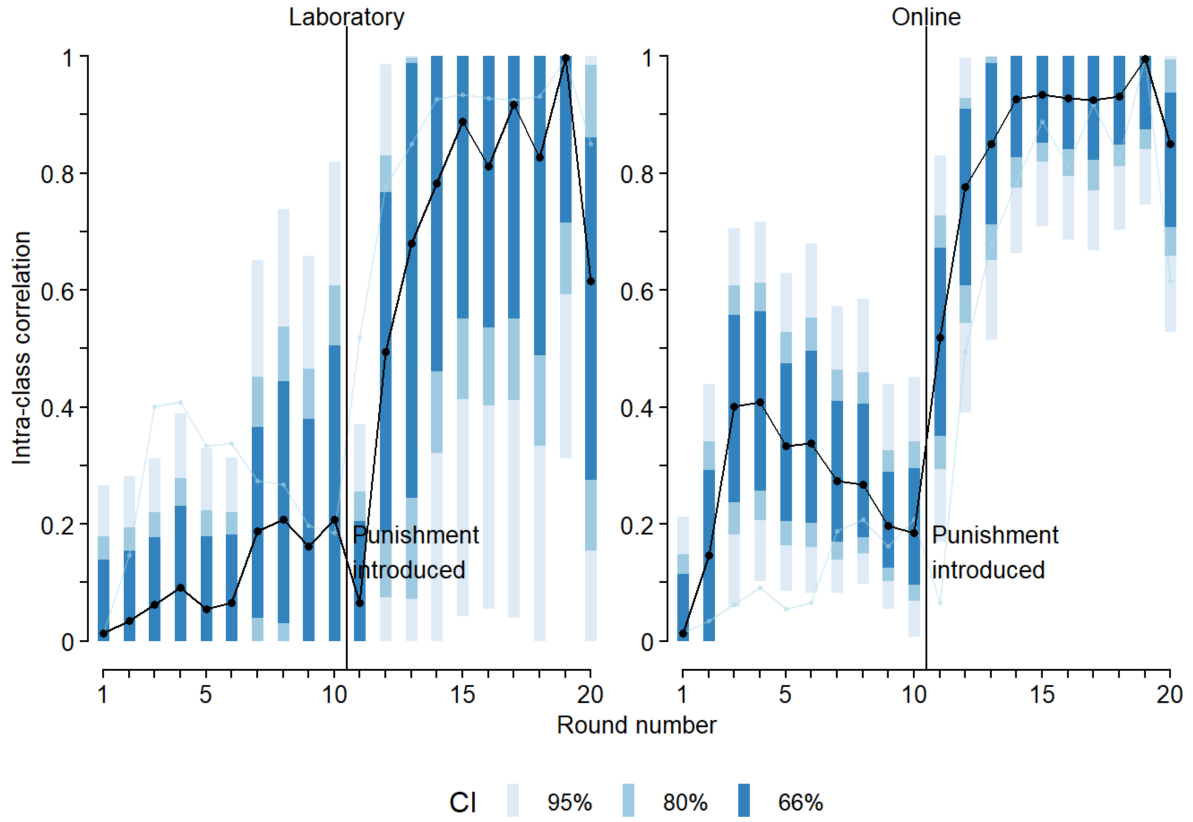

Fig. S67. Intra-class correlation across rounds of play in each experimental condition.

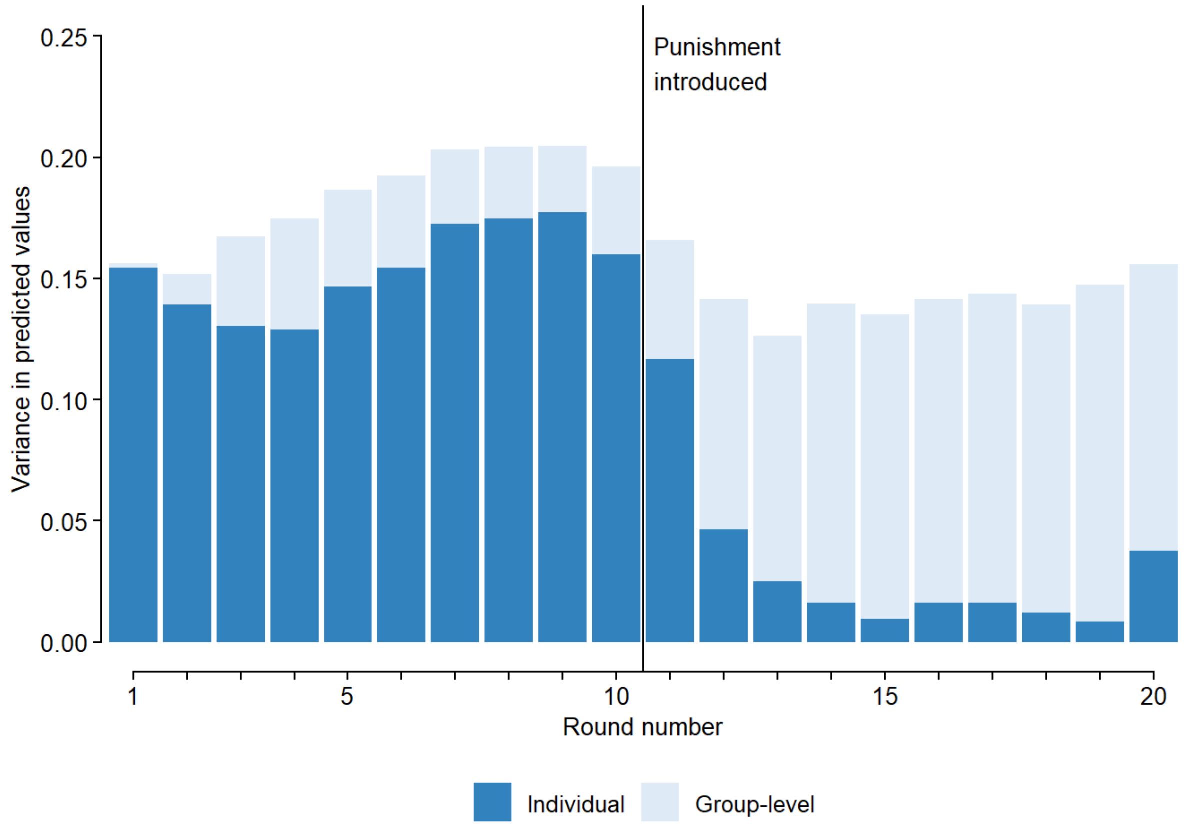

Fig. 68. Variance partitions across rounds of play.

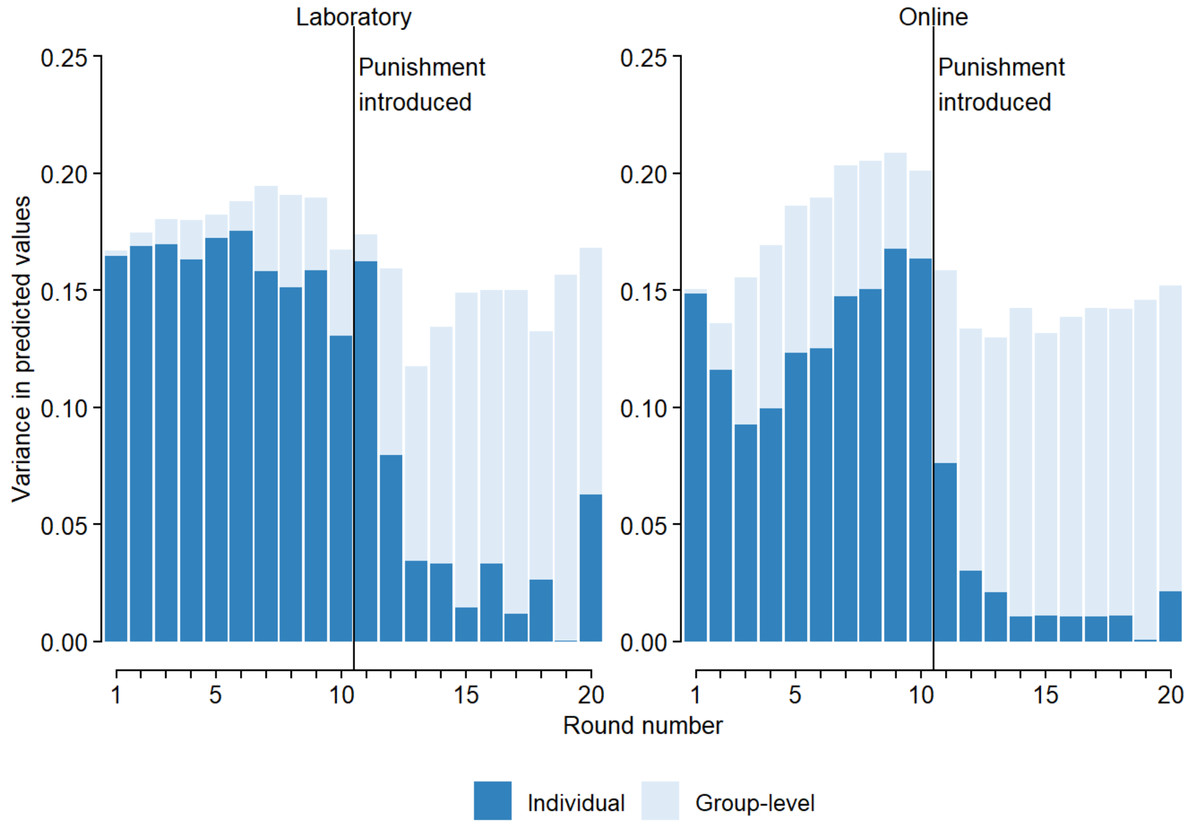

Fig. S69. Variance partitions across rounds of play in each experimental condition.

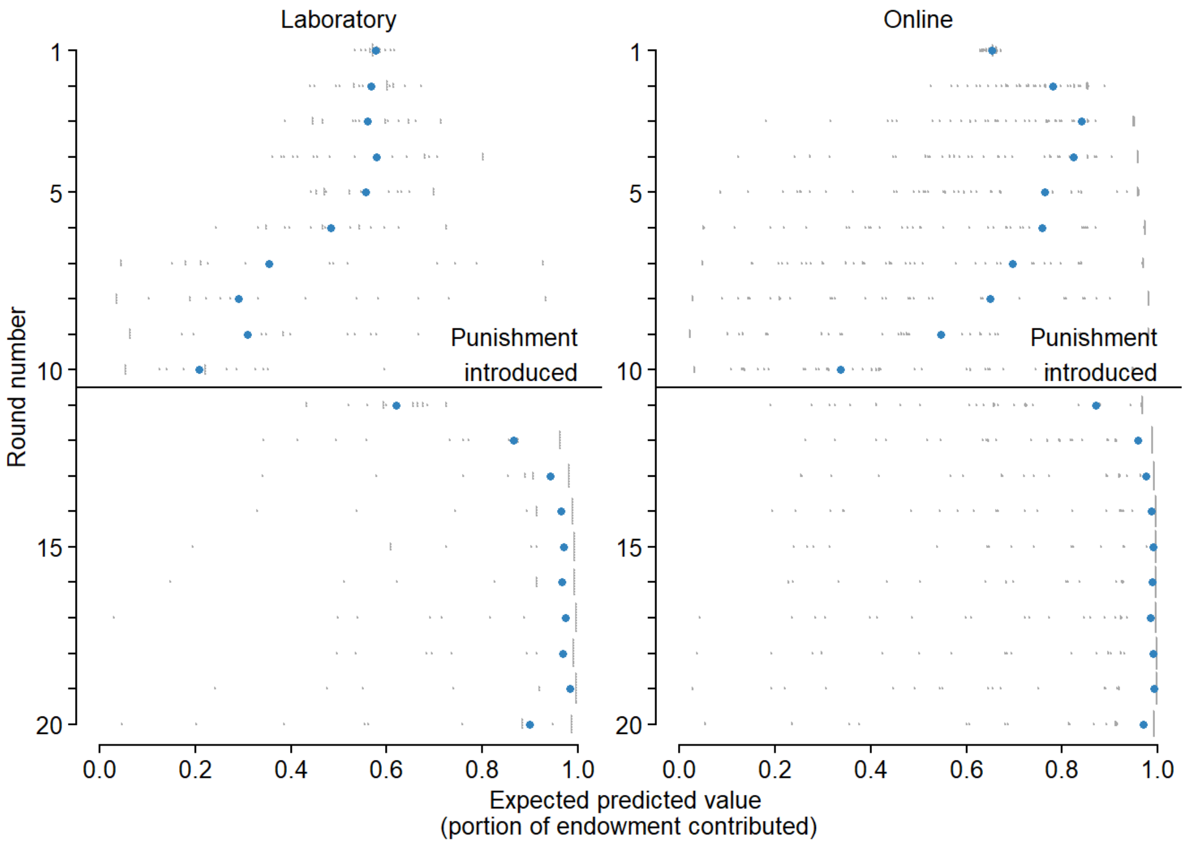

Fig. S70. Overall (blue dot) and group-level (grey dots) predictions across rounds of play in each experimental condition.
